# Total Synthesis and Biological Evaluation of Leptosphaerone B and Derivatives of Microketide A

**DOI:** 10.1021/acs.jnatprod.5c01581

**Published:** 2026-02-13

**Authors:** Martin F. Köllen, Stephan A. Sieber

**Affiliations:** TUM School of Natural Sciences, Department of Bioscience, Center for Functional Protein Assemblies (CPA), Chair of Organic Chemistry II, Technical University of Munich, 85748 Garching bei München, Germany

## Abstract

Microketide A and B are fungal polyketides reported to display potent activity against Gram-negative pathogens, yet the lack of synthetic access has prevented detailed investigation of their mode of action and structure–activity relationship (SAR). Here, we report the first total synthesis of two close analogs of microketide A, dihydro-MikA and 11-deoxy-MikA, as well as of racemic leptosphaerone B, another member of this cyclohexenone-based natural product family. Our route features a modular assembly of highly functionalized fragments and enables divergent access to analogs through selective dihydroxylation and late-stage fragment fusion. Despite extensive exploration of multiple C–C bond-forming strategies, unfavorable sterics and competing eliminations prevented successful connection of the fragments required for microketide A. The synthesized compounds leptosphaerone B, dihydro-MikA, and 11-deoxy-MikA were evaluated for antibacterial activity and human cytotoxicity but showed no effects up to 200 μM. Competitive residue-specific chemoproteomics and in vitro nucleophile-trapping experiments further revealed no covalent protein engagement, indicating that these scaffolds are intrinsically weak electrophiles. Our findings suggest that microketide A possesses a restrictive SAR, in which even subtle modifications abolish biological function. The synthetic strategy described herein provides a robust platform for in-depth structure activity relationship studies assessing the biological potential of this natural product.

## Graphical abstract

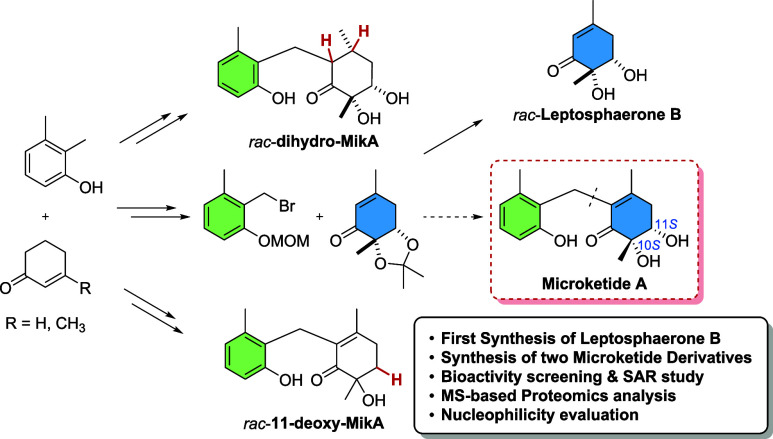

Despite the progress in rational and AI-guided drug design, natural products remain an important source for lead structures in the development of novel antibiotics. In 2020, Liu et al. isolated Microketide A and B (**1** and **2**), two C-11 epimers of a methylene-bridged bicyclic scaffold, from gorgonian-derived sea fungus *Microsphaeropsis sp.* RA10–14.^1^ They are reported to have submicromolar antibiotic activity against multiple clinically relevant Gram-negative pathogens like *P. aeruginosa* ((**1**) 0.69 μM, (**2**) 5.7 μM) and *Salmonella Typhi* (both 0.69 μM). They share their core motif, a cyclohexenone decorated with two methyl groups and two hydroxy groups, with three other fungal polyketides, leptosphaerone A and B, and phomopine (**3–5**). The leptosphaerones were first isolated from grass pathogenic fungus *Leptosphaeria herpotrichoides* in 1993 by Ayer et al. and have since then been reisolated from multiple other fungi of the genera *Penicillium* and *Polycephalomyces*.^2–5^ (−)-Leptosphaerone A ((−)-**3**), sharing the *anti*-configuration of its diol with microketide B (**2**), was found to be cytotoxic against A-549 cells,^3^ while another study did not observe any toxicity in MCF-7, KB, and Vero cells.^4^ However, there is no data on the antibiotic properties of leptosphaerone A (**3**), and for leptosphaerone B (**4**), which shares the *syn*-configuration of its diol with microketide A (**1**), there is no data on biological activity at all. The last member of the class, phomopine (**5**), was isolated from plant pathogenic fungus *Phomopsis* sp. XM-01 in 2024 by Wang et al. and is reported to have moderate antifungal activity against *Candida albicans* , but no activity against *Staphylococcus aureus*.^6^ 
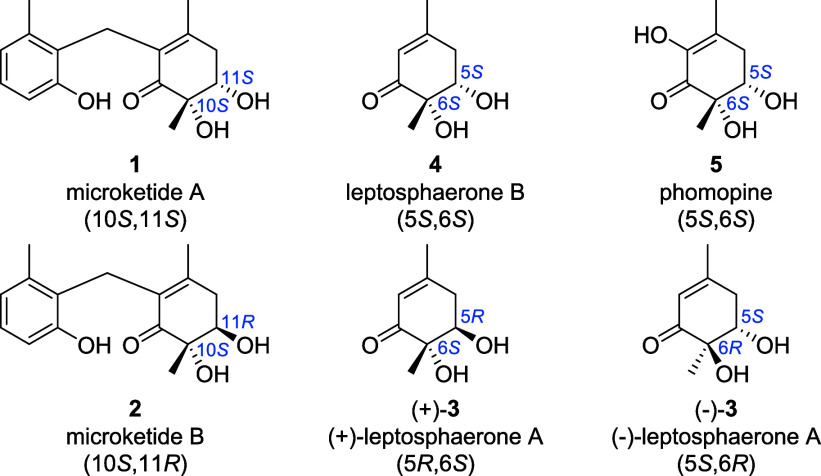


Due to the strong antibiotic activity of the microketides, the highly conserved cyclohexenone motif with its potential for covalent reactivity, and the structural dissimilarity of this family of natural products to any known antibiotics, we became interested in developing their first total synthesis and elucidating their antibiotic mode of action. We decided to focus on microketide A (**1**) as the main synthetic target, as it showed the highest antibiotic potency in the studies of Liu et al. A theoretical route for a total synthesis of microketide A was proposed by Wu in 2021; however this proposal was never followed up.^7^ We, therefore, designed a robust synthesis route which conveniently provides leptosphaerone B (**4**) as an intermediate and two close analogs of microketide A, dihydro-MikA (**38**), and 11-deoxy-MikA (**46**) as starting points for bioactivity studies.

## Results and Discussion

### Retrosynthetic Planning

From a retrosynthetic aspect, we envisioned a late-stage fusion of the two already functionalized fragments **6** and **7** as the key step (Scheme 1A). If this strategy fails, the connection of ketone **8** to benzyl bromide **6** via enolate alkylation could be pursued, followed by selective introduction of the α,β-double bond in the final scaffold (Scheme 1B). Prior to the coupling, enone **7** could be retrosynthetically obtained from ketone **8** by dehydrogenation, while ketone **8** could be accessed from cyclohex-2-en-1-one (**10**) by α-methylation, *syn*-dihydroxylation, and acetal protection to dihydroxyketone **9**, followed by α,β-dehydrogenation and conjugate addition of a cuprate (Scheme 1C). Benzyl bromide **6** could be obtained from dimethylphenol **12** by MOM-protection and regioselective Wohl-Ziegler bromination (Scheme 1D).

**Scheme 1:**
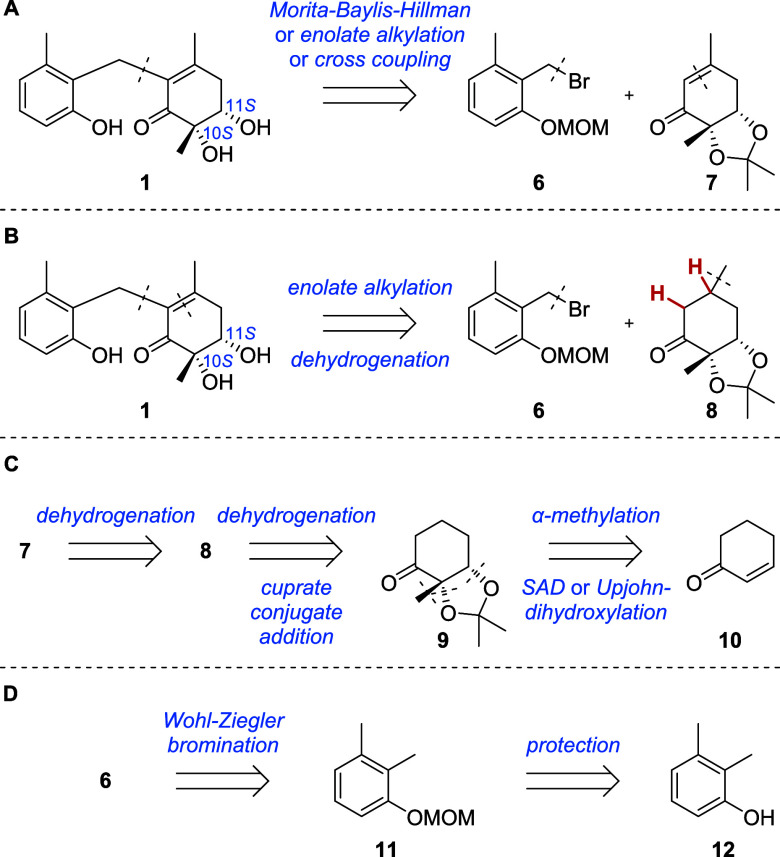
Retrosynthetic Analyses for Microketide A (**1**)

### Synthesis of Microketide A Fragments and *rac*-Leptosphaerone B

Following the retrosynthetic considerations, our synthesis started with the MOM-protection of 2,3-dimethylphenol (**12**, Scheme 2A) using sodium hydride and methoxymethyl chloride to give protected phenol **11** in good yield. Subsequent Wohl-Ziegler bromination afforded benzyl bromide **6** with complete regioselectivity.^8,9^ Notably, efforts to replace CCl_4_ by common alternatives like CHCl_3_ or DCE led to complex mixtures of multibromination products, while an alternative route via the regioselective oxidation of the 2-methyl group to the corresponding benzaldehyde with peroxosulfate,^10^ DDQ^11^ or CAN^12^ did not work under any of the tested conditions.

**Scheme 2:**
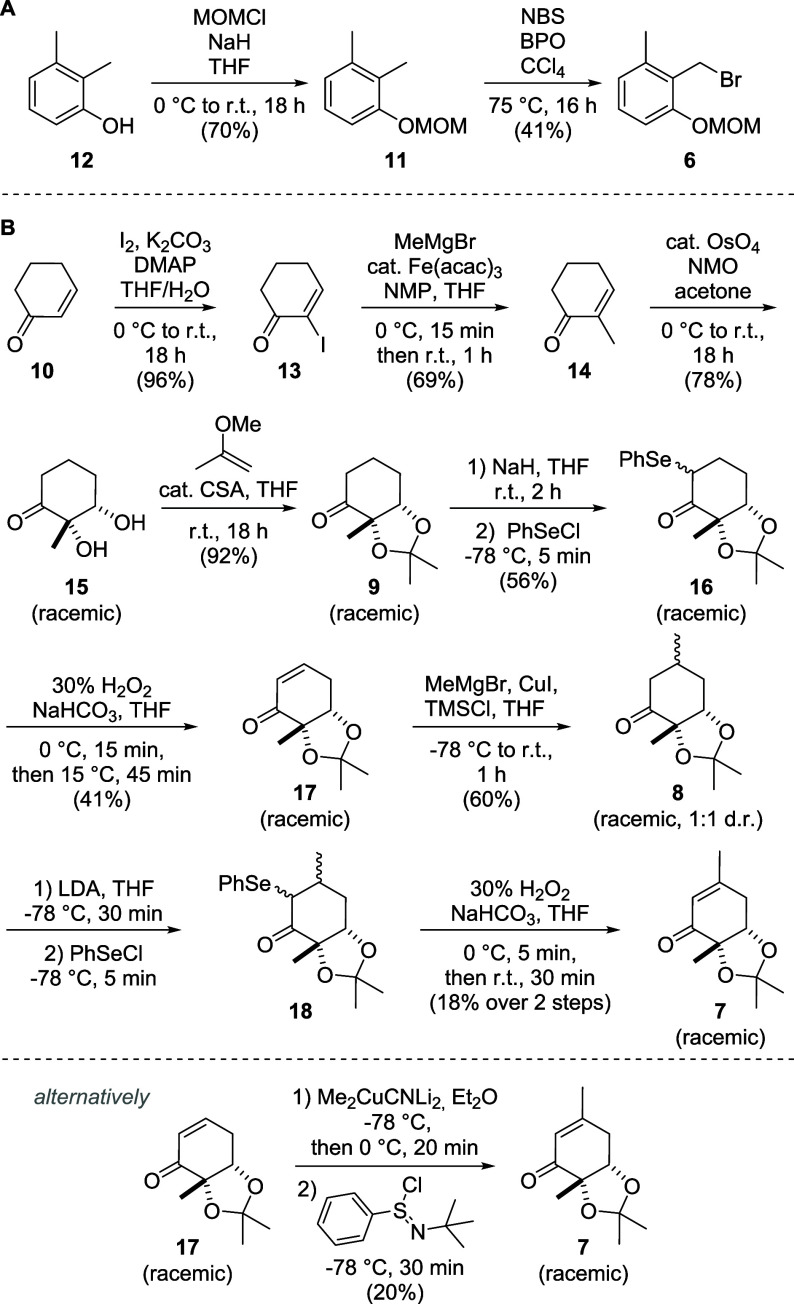
Synthesis of Fragments **6** (A) and **7** (B)

The synthesis of fragment **7** (Scheme 2B) commenced with the α-iodination of cyclohex-2-en-1-one (**10**) through a Morita-Baylis-Hillman-like mechanism using DMAP as the nucleophilic catalyst, affording α-iodoenone **13** in almost quantitative yield.^13–15^ A subsequent iron-catalyzed cross-coupling with methylmagnesium bromide yielded the methylated cyclohexenone **14**.^15–17^ In the next step, the differentiation between microketide A and B can be achieved by choosing a *syn*- or *anti*-selective dihydroxylation reaction. For microketide A, an osmium-catalyzed Upjohn-dihydroxylation was employed, while for microketide B a chiral epoxidation, followed by epoxide hydrolysis can be conceived.^18^ However, our efforts to achieve enantioselectivity remained unsuccessful. While the classic Upjohn-conditions using catalytic amounts of OsO_4_ in combination with NMO as the reoxidant gave good yields of racemic diol **15**,^13,14,19,20^ we did not obtain any product when using commercial AD-mix for Sharpless asymmetric dihydroxylation.^21–23^ Using a reversibly covalent sulfoximine as chiral auxiliary for an enantioselective dihydroxylation failed, as the thermolysis to cleave off the auxiliary led to decomposition of the product (Supplementary Scheme S1).^24–27^ We, therefore, decided to continue with the racemic diol to produce racemic microketide A and then try a chiral resolution by chiral HPLC. Diol **15** was protected as an acetonide (**9**) by excess 2-methoxypropene in the presence of catalytic amounts of camphorsulfonic acid (CSA) in very high yields.^13,14^ To dehydrogenate ketone **9** to enone **17**, a selenoether was introduced in the α-position of the ketone by a reaction of phenylselenyl chloride with the enolate of **9**, giving α-selenoether **16** as a single diastereomer in moderate yield. As the stereocenter is lost in the next step, the relative configuration was not determined. Oxidation of the selenoether to the selenoxide by hydrogen peroxide led to its spontaneous elimination, furnishing the α,β-unsaturated cyclohexenone **17** in moderate yield.^28,29^ While the use of alternative oxidants like *m*-CPBA or the addition of an amine base are described in the literature to suppress side reactions of the elimination,^28^ both did not improve the yield. Conjugate addition of a cuprate led to the methylated cyclohexanone **8** in good yield.^14,30,31^ Interestingly, when methylmagnesium bromide was used in combination with catalytic amounts of copper­(I) iodide, a mixture of diastereomers (*syn*-**8** and *anti*-**8**, Scheme 3) formed, with *syn*:*anti*-ratios between 2:1 and 1:1. When a Gilman cuprate was preformed from methyllithium and copper­(I) cyanide before the addition of the enone, almost exclusively *anti*-**8** was obtained (ratio 1:10). The relative configuration at the β-carbon in *syn*-**8** was determined by NOESY NMR in *syn*-**35** after a subsequent coupling step (Scheme 8).

**Scheme 3:**
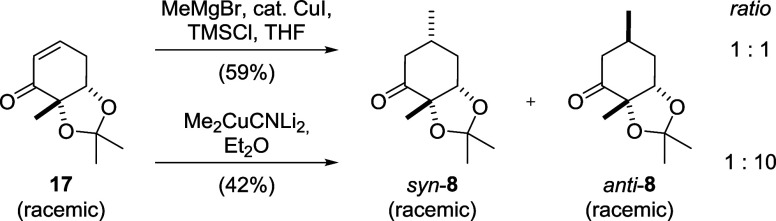
Cuprate Dependence of the s*y* *n*-*anti*-Selectivity of the Conjugate Addition to Cyclohexenone **17**

Both diastereomers were dehydrogenated via α-selenoether **18** resulting in fragment **7** with a yield of 18% over two steps (Scheme 2). Alternatively, the last three steps from enone **17** to enone **7** were combined into a one-pot transformation by using a Gilman cuprate and treating the resulting methylated enolate in situ with *N*-*tert*-butyl phenylsulfinimidoyl chloride, a reagent developed by Mukaiyama et al. in 2000.^32–34^ However, as the yield was only slightly higher than the combined yield of the three-step procedure, and as the reagent needed to be prepared in situ over two steps (Supplementary Scheme S2), this route does not represent a significant improvement over the three-step procedure. With fragment **7** in hand, racemic leptosphaerone B (*rac*-**4**) was synthesized by deprotecting the diol using hydrogen chloride in MeOH (Scheme 4).

**Scheme 4:**
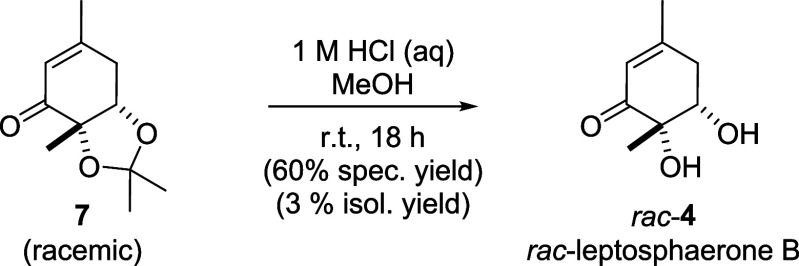
Synthesis of *rac*-Leptosphaerone B (*rac*-**4**) through Deprotection of Fragment **7**

### Exploiting Coupling Strategies for the Fusion of the Two Microketide A Fragments

In order to fuse fragments **6** and **7** into the final scaffold of microketide A (**21**), we established working conditions for multiple different coupling strategies, using simplified analogs like verbenone (**19a**) or 3-methylcyclohexenone (**19b**) and then transferred the optimized procedures to the real scaffolds (Scheme 5). First we tried a base mediated coupling via a dienolate.^35–38^ While we managed to achieve good yields of **20a,b** when coupling benzyl bromide or fragment **6** to verbenone (**19a**), the original fragment **7** decomposed under the strong alkaline conditions, probably eliminating the acetonide (Scheme 5A and Supplementary Scheme S3). Gradillas et al. developed a procedure for a Morita-Baylis-Hillman (MBH) alkylation catalyzed by phenylthioethanol, expanding the scope of electrophiles of the classical MBH reaction from aldehydes to alkyl and benzyl halides.^39^ However, we did not observe any conversion of our enones (**19a,b**) under the described conditions, nor with the conditions used for the preparation of α-iodoenone **13** with DMAP as catalyst (Scheme 5B). The use of benzaldehyde **22** as electrophile in combination with classical MBH catalysts like DABCO and imidazole on verbenone (**19a**) did not afford benzylic alcohol **23** either. We suspect that this is due to the increased steric hindrance and electron-donating properties of the β-methyl group. This is corroborated by studies of Mayer et al., who showed that β-substituted cyclic enones are less electrophilic than unsubstituted cyclic enones by several orders of magnitude.^40^

**Scheme 5:**
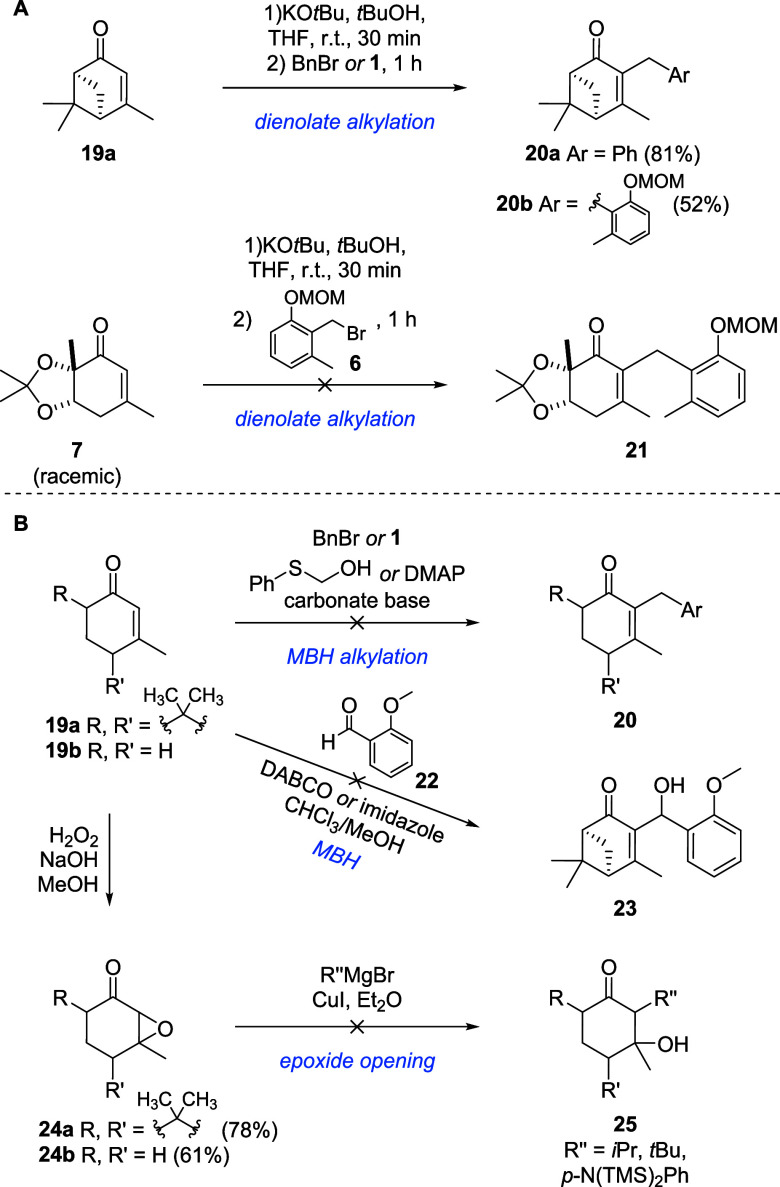
Coupling Efforts of Fragments **6** and **7** and Their Simplified Analogs via (A) Base-Mediated Dienolate Alkylation or (B) Morita-Baylis-Hillman Variations or Epoxide Opening

Next, we tried to react α,β-epoxy ketones (**24a,b**) with cuprates to obtain α-alkyl-β-hydroxycyclohexanones (**25**),^41,42^ from which the desired α-alkylcyclohexenones could be synthesized by elimination of the hydroxy group. While the epoxidation worked well,^43^ epoxides **24a,b** did not react with any of the provided cuprates.

An alternative approach represents the coupling of the fragments via Negishi cross-coupling, for which cyclohexenone **7** was α-iodinated. As the standard MBH iodination conditions using iodine and DMAP or pyridine did not work with the highly substituted and electron deficient enone **7**, the reactivity was increased by adding phenyliodine bis­(trifluoroacetate) (PIFA) to oxidatively generate trifluoroacetyl hypoiodite in situ,^44,45^ while radical side reactions were inhibited by the addition of butylated hydroxytoluene (BHT).^46,47^ This way, we could obtain **26**, albeit in low yield (Scheme 6A). However, the zincation of benzyl bromide **6** and subsequent Negishi reaction of benzyl zinc species **27** with iodoenone **26** only led to formation of homodimer **28** through Wurtz coupling.^48^ To prevent Wurtz coupling, the complete conversion of the benzyl bromide into the zinc species is crucial, and for optimal results of the Negishi coupling, it is important to add the zinc species at a high concentration. Using benzyl bromide (**29a**), we optimized the zinc insertion according to a protocol from Metzger et al.,^49^ achieving a concentration of 2.0 M determined by iodometric titration,^50^ which corresponds to a yield of 99% (Scheme 6B). Using this zinc species (**30a**), the Negishi coupling with iodoenone **13** was successful and gave **31a** with a 52% yield. However, while the exchange of the MOM-protection group on the phenol for acetate (**29b**) helped to improve the zinc insertion yields, we could not improve the yield of **30b** to exceed 20%, resulting in benzyl zinc concentrations of 0.20 M. Even after additional concentration of the benzyl zinc species by partial evaporation of the solvent in vacuo, the Negishi coupling with iodoenone **13** toward **31b** did not work.

**Scheme 6:**
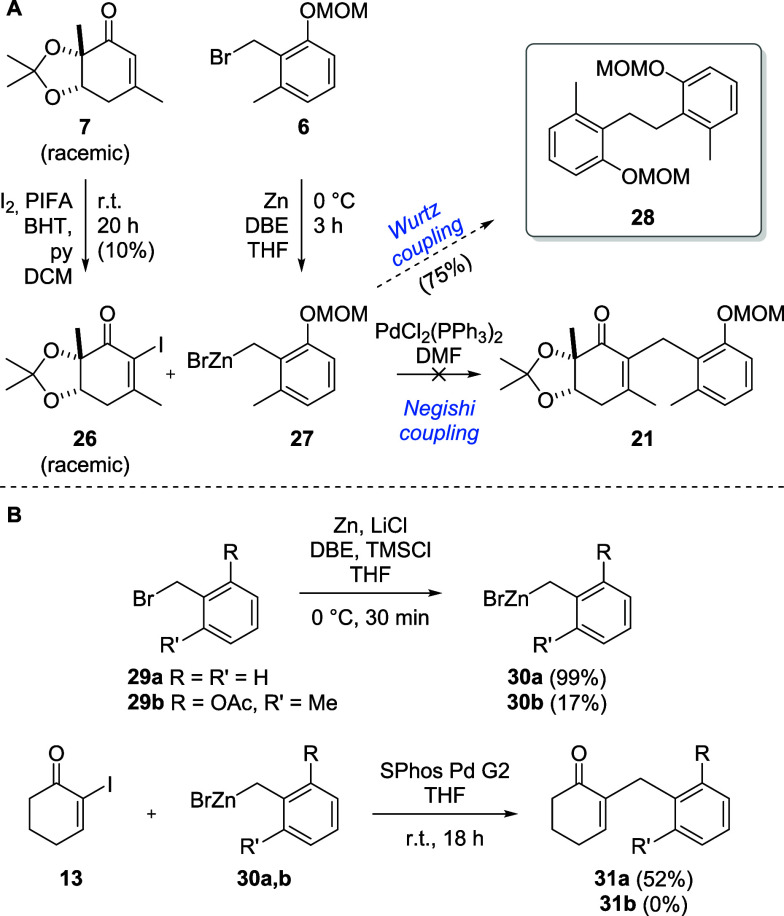
Efforts to Couple Fragments **6** and **7** via Negishi Cross-Coupling (A) and Optimization of Metalation and Negishi Cross-Coupling Conditions Using Simplified Analogs (B)

Similar to the Negishi coupling, we tried to establish a route via Stille coupling using simplified fragments (Scheme 7). While the stannylation of iodoenone 3**2** furnished α-stannylenone **33** in moderate yield,^51^ and Stille coupling of benzyl bromide (**29a**) to **33** was successful,^52^ the reactions of the functionalized benzyl bromides (**6**, **29b**) with α-stannylenone **33** did not give the desired products (**34b,c**).

**Scheme 7:**
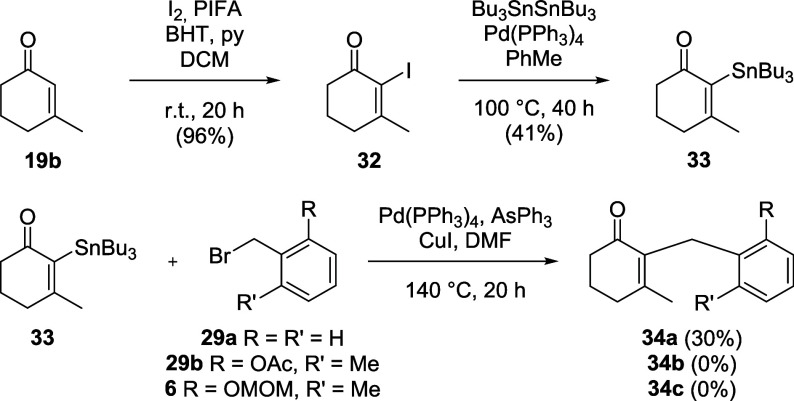
Efforts to Establish Conditions to Couple the Fragments via Stille Coupling

As all our efforts to couple the enone to a benzylic position were unsuccessful, we took a step back and decided to try to form the crucial C–C-bond between both fragments before the introduction of the α,β-double bond, as the coupling of the enolate of **8** with phenylselenyl chloride worked (Scheme 2B). When we subjected a *syn*- and *anti*-mixture of methylcyclohexanone **8** to LDA or sodium hydride and benzyl bromide **6**, we observed that only the *syn*-diastereomer was converted into the α-benzylated product *syn*-**35**, while the *anti*-diastereomer did not react and was reisolated (Scheme 8). *Syn*-**35** was obtained as a single diastereomer and the configuration at the newly formed stereocenter was determined to be *anti* to both the methyl group in the β-position and the protected diol by NOESY NMR.

**Scheme 8:**
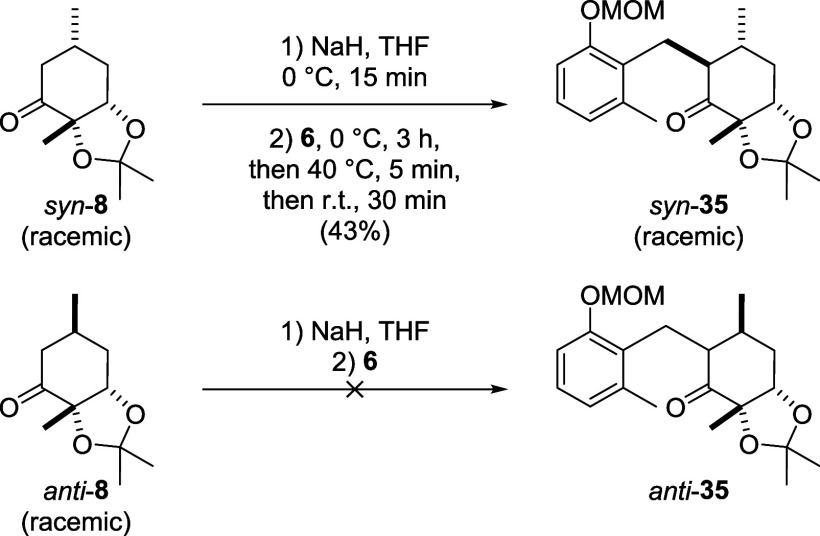
Diastereoselective Enolate Coupling of Cyclohexanone **8** and Benzyl Bromide **6**

We proceeded to test several methods to dehydrogenate the α-benzylated ketone *syn*-**35**. The selenylation that we used before did not work on this substrate, probably due to steric hindrance. α-Bromination using copper­(II) bromide remained unsuccessful, too.^53^ We further tried direct oxidations with 2-iodoxybenzoic acid (IBX)^54,55^ or Pd­(TFA)_2_(DMSO)_2_ and O_2_,^56^ as well as preparing the silyl enol ether of the ketone and subsequently dehydrosilylating it through a Saegusa-Ito oxidation,^57,58^ neither of which yielded the desired product (Scheme 9).

**Scheme 9:**
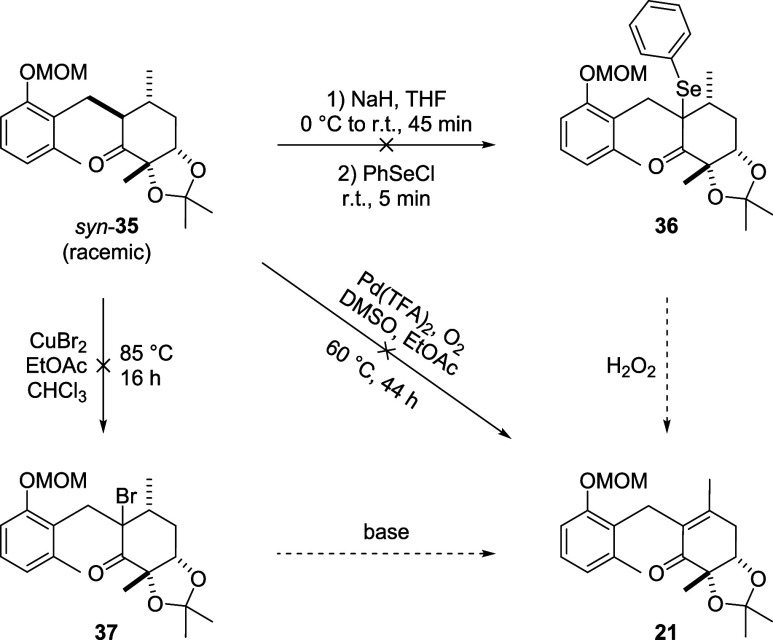
Unsuccessful Dehydrogenation of Cyclohexenone **35** towards Protected Microketide A (2**1**)

### Synthesis of Microketide A Analogs

Given the challenges associated with coupling of the original fragments, we decided to synthesize close analogs of microketide A instead, utilizing the knowledge we gained on the reactivity of the precursors. This way, we envisioned to obtain insights into the structure–activity-relationship and possibly the mode of action of the microketides even though the original natural product remains elusive. By deprotecting cyclohexanone **35**, we obtained a saturated dihydromicroketide analog as a racemate (*rac*-dihydro-MikA, **38** Scheme 10A). As the base-mediated dienolate alkylation of cyclohexenones generally worked, but led to decomposition of the protected diol (vide supra, Scheme 5A), we hypothesized that a cyclohexenone scaffold lacking the oxygen substituent in position 11, which is prone to elimination, should be compatible with the optimized conditions. We synthesized MOM-protected hydroxy cyclohexenone **43** starting from 3-methylcyclohexenone (**19b**, Scheme 10B). α-Methylation of **19b** gave **39**, which was hydroxylated to α-hydroxy ketone **42** by Rubottom oxidation via silyl enol ether **40** and epoxy silyl ether **41**. Subsequent MOM-protection furnished **43** in a high yield of 60% over four steps. Dienolate alkylation with benzyl bromide **6** proceeded quantitatively to give **44**, which was deprotected under acidic conditions, furnishing racemic 11-deoxy-microketide A (11-deoxy-MikA, **46**). The yield of the deprotection was very low due to formation of large quantities of the concurring elimination product **45**, which can be explained by the gain of aromaticity through the elimination.

**Scheme 10:**
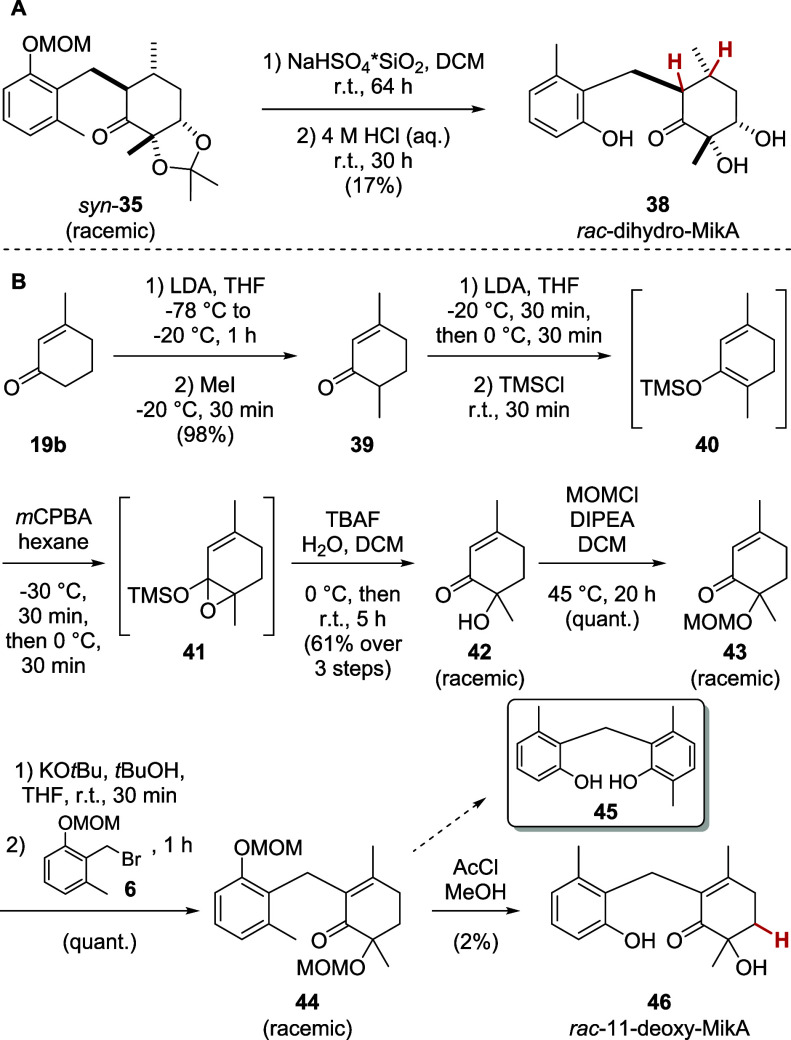
Synthesis of Microketide A Analogs: (A) *rac*-dihydro-MikA (**38**) and (B) *rac*-11-deoxy-MikA (**46**)

The differences of the analogs compared to the original natural product are highlighted in red.

### Biological Testing of Leptosphaerone and Microketide A Analogs

With *rac*-leptosphaerone B (*rac*-**4**), *rac*-dihydro-MikA (**38**) and *rac*-11-deoxy-MikA (**46**) in hand, we set out to determine their bioactivity. To our surprise, none of them showed any antibiotic activity up to a concentration of 200 μM against a broad panel of bacteria similar to that of the original publication of microketide A by Liu et al. ( *Bacillus subtilis* 168, *Bacillus licheniformis* ATCC 14580, *Escherichia coli* K12, *Klebsiella aerogenes* DSM 30053, *Staphylococcus aureus* USA300, *Streptococcus pyogenes* ATCC 700294, *Pseudomonas aeruginosa* PAO1, and *Salmonella enterica* subsp. enterica LT2).^1^ They also did not show an IC_50_ in human embryonic kidney cells (HEK 293) within the concentration range tested, although metabolic activity was slightly reduced at 200 μM (Supplementary Figure S1).

Many natural products exert their function by covalent engagement with protein targets in the cell. We thus tried to identify potential covalent interactions of the two compounds possessing a Michael acceptor, *rac*-leptosphaerone B (*rac*-**4**) and *rac*-11-deoxy-MikA (**46**), with cysteines in the proteome of *E. coli* . For this, we used competitive residue-specific chemoproteomics, employing iodoacetamide alkyne as a globally reactive cysteine probe and isotopically labeled desthiobiotin azide (isoDTB) tags for enrichment and MS based quantification (Supplementary Figure S2).^59,60^ As we did not observe any significantly enriched peptides, we examined the in vitro reactivity of *rac*-leptosphaerone B (*rac*-**4**) and *rac*-11-deoxy-MikA (**46**) with 10-fold excess of several thiol-based nucleophiles (*N*-acetylcysteine, glutathione, β-mercaptoethanol), as well as other nucleophilic amino acids (*N*-α-acetyllysine, *N*-Cbz-serine) by mass spectrometry. In line with the proteomics data, we did not observe the formation of any adducts under the tested conditions, leading us to the conclusion that the scaffolds are very weak electrophiles which are unreactive toward biological targets (Supplementary Figure S3). As the study of Mayer et al. has shown that substituents remote from the electrophilic π-system have only a minor impact on the electrophilicity of 3-methylcyclohexenones,^40^ we assume that the electrophilicity of microketide A does not differ significantly from that of *rac*-leptosphaerone B (*rac*-**4**) or *rac*-11-deoxy-MikA (**46**), rendering a covalent mode of action of microketide A unlikely.

To identify interactions of our compounds with the proteome of bacteria beyond covalent reactivity or toxic effects, we performed a full proteome analysis of *S. aureus* treated in situ with our compounds (*rac*-leptosphaerone B (*rac*-**4**), *rac*-dihydro-MikA (**38**), *rac*-11-deoxy-MikA (**46**)) for 1 h. The only protein that was consistently upregulated for all three compounds compared to a DMSO control was the multidrug efflux transporter MmpL (Supplementary Figure S4). For *rac*-11-deoxy-MikA (**46**), STRING analysis showed the upregulation of proteins from clusters associated with the degradation of xylene and other aromatic compounds. For the other compounds, no functional assignments of significantly up- or downregulated proteins were possible. These results show that all three compounds have no direct effect on the cellular processes of *S. aureus*, except the activation of protection mechanisms to remove or degrade the foreign molecules. This is in line with the results from the activity assays and chemoproteomics experiments and suggests that leptosphaerone B (**4**) and our two close analogs of microketide A (**38**, **46**) lack bioactivity.

## Conclusions

In conclusion, we successfully performed the first racemic total synthesis of leptosphaerone B and synthesized two very close analogs of microketide A, *rac*-dihydro-MikA (**38**) and *rac*-11-deoxy-MikA (**46**), only differing from the original scaffold in a saturation of the α,β-double bond of the enone or the lack of the second hydroxy group at C-11. Despite our extensive efforts to find suitable conditions for the fusion of the fully functionalized fragments **6** and **7**, the original natural product microketide A remains elusive. Despite the exploitation of multiple coupling strategies, the ligation of both fragments is most likely challenged by steric hindrance, as the *ortho*-positions of both fragments are substituted, as well as by the elimination of oxygen substituents in fragment **7** to gain aromaticity.

We examined the biological activity of *rac*-leptosphaerone B (*rac*-**4**), *rac*-dihydro-MikA (**38**), and *rac*-11-deoxy-MikA (**46**) against a broad panel of bacteria, as well as human embryonic kidney cells, observing no activity up to 200 μM. Full proteome analysis of *S. aureus* treated with high concentrations of our compounds did not show changes in the proteome except the upregulation of defense mechanisms such as efflux pumps and degradation clusters. *rac*-Leptosphaerone B (*rac*-**4**) and *rac*-11-deoxy-MikA (**46**) did not form adducts with the side chains of nucleophilic amino acids in vitro and competitive residue-specific chemoproteomics revealed no significant covalent modification of proteins in lysate of *E. coli* . Given the potent antibiotic activity reported for mikroketide A, it is surprising that already the lack of a nonreactive Michael acceptor or a hydroxy group fully abolishes bacterial killing. This suggests a very restricted structural flexibility which needs to be further exploited by the synthesis of analogs. Our synthetic route, although not able to provide the natural product, represents a robust platform for exploiting alternative modifications for in-depth structure activity relationship studies assessing the potential of this natural product.

## Experimental Section

### Chemical Synthesis

Detailed experimental procedures for the synthesis of all compounds mentioned in this article, as well as analytical data for all products and intermediates are provided in the Supporting Information, accompanied by general considerations on synthesis methods, sources of starting materials and analytical methods used in this project.

### Biochemical Methods

Detailed descriptions of all biological and biochemical assays and experiments mentioned in this article, as well as lists of materials and organisms used, along with the corresponding suppliers and the composition of all media and buffers are provided in the Supporting Information. All assays and experiments are briefly summarized below.

### Minimal Inhibitory Concentration (MIC) Assay

Antibacterial activity of our compounds against a variety of bacteria was evaluated by determination of their minimum inhibitory concentrations (MICs) in 96-well format using a broth microdilution method adopted from the Clinical and Laboratory Standards Institute (CLSI) guideline^61^ and individual growth conditions for the respective bacteria (as stated in the Supporting Information).

### Cytotoxicity Determination via Metabolic Activity Measurement (MTT Assay)

Cytotoxicity of our compounds in human embryonic kidney (HEK 293) cells was evaluated by measuring the metabolic activity of the cells via MTT assay after 24 h treatment of the cells with medium containing different concentrations of our compounds. The cells were grown in poly-l-lysin coated 96-well plates and incubated at 37 °C and 5% CO_2_. Metabolic activity was determined by quantifying the metabolic conversion of 3-(4,5-dimethyl-2-thiazolyl)-2,5-diphenyl-2H-tetrazolium bromide to formazan within 1 h, which was done by measuring the absorbance at 570 nm (formazan) vs background (630 nm).

### Competitive Residue-Specific Chemoproteomics

Analysis of covalently modified protein targets of *rac*-11-deoxy-MikA (**46**) and *rac*-leptosphaerone B (*rac*-**4**) in *E. coli* K12 was performed through competitive, residue-specific proteomics using the isoDTB-ABPP platform.^59,60^ Bacteria were lysed under nondenaturing conditions and the lysates were treated with either *rac*-11-deoxy-MikA or *rac*-leptosphaerone B (200 μM), or DMSO (1%) for 1 h at room temperature. The treated samples were subsequently labeled with IA-alkyne (1 mM) and clicked to either a light (compound) or heavy (DMSO) isoDTB-tag. Samples were combined in a 1:1 ratio with their respective DMSO-control, enriched on streptavidin beads, tryptically digested, and analyzed via LC-MS/MS. The ratio between detected heavy- and light-tagged peptides indicates which cysteines are covalently engaged with the compounds.

### In Vitro Reactivity Profiling

Enones *rac*-leptosphaerone B (*rac*-**4**) and *rac*-11-deoxy-MikA (**46**) were incubated with 10-fold excess of thiol-based biological nucleophiles and nucleophilic amino acids at pH 9.0 at 37 °C for 1 h before the formation of adducts was examined via HPLC-MS.

### Full Proteome Analysis

For whole proteome analyses according to a procedure by Schum et al.,^62^ cultures of *S. aureus* NCTC 8325 were treated for 1 h at 37 °C with *rac*-11-deoxy-MikA (**46**), *rac*-dihydro-MikA (**38**) or *rac*-leptosphaerone B (*rac*-**4**), before the cells were lysed and the proteome was isolated, tryptically digested, and analyzed via LC-MS/MS. The quantities of all proteins were determined using label-free quantification and the values were compared to a DMSO control.

## Supplementary Material





## Data Availability

The mass spectrometry proteomics data have been deposited to the ProteomeXchange Consortium via the PRIDE partner repository^63^ with the data set identifier PXD071945. Raw NMR data of final compounds (*rac*-**4**, **38**, and **46**) and the crucial intermediate (*syn*-**35**) for determination of the relative configuration of **38** are made available in the Supporting Information of this publication.

## Notes

Supplementary data, detailed experimental procedures, analytical data, and figures of ^1^H and ^13^C NMR spectra (PDF)
Raw NMR data of compounds *rac*-**4**, **38**, **46,** and *syn*-**35** (ZIP)
