# Supplementary material for: Total Synthesis and Biological Evaluation of Leptosphaerone B and Derivatives of Microketide A
Source: J Nat Prod. 2026 Feb 13;89(2):737–45. doi: 10.1021/acs.jnatprod.5c01581 (PMC12954852; doi:10.1021/acs.jnatprod.5c01581)

Supporting Information

Total Synthesis and Biological Evaluation of Leptosphaerone B and Derivatives of Microketide A

*Martin F. Köllen and Stephan A. Sieber**

TUM School of Natural Sciences, Department of Bioscience, Center for Functional Protein Assemblies (CPA), Chair of Organic Chemistry II, Technical University of Munich, 85748 Garching bei München, Germany

*Correspondence: stephan.sieber@tum.de

Table of Contents

1 Supplementary Schemes, Figures and Tables 4

2 Chemical Synthesis Methods 9

2.1 General Considerations 9

2.2 Detailed Experimental Procedures for All Compounds 13

3 Biochemical Methods 41

3.1 Materials 41

3.2 Experimental Procedures 44

4 References 55

5 Appendix 57

5.1 List of Abbreviations 57

5.2 NMR Spectra 60

Table of Items

Supplementary Scheme S1: Enantioselective *syn*-dihydroxylation using a chiral sulfoximine auxiliary 4

Supplementary Scheme S2: Synthesis of *N*-*tert*-butylbenzenesulfinimidoyl chloride 4

Supplementary Scheme S3: Mechanism of the dienolate alkylation reaction and a hypothetical mechanism for the concurring elimination of the acetonide protection group under alkaline conditions 4

Supplementary Figure S1: Relative metabolic activity of HEK 293 cells after treatment with *rac*-11-deoxy-MikA, *rac*-dihydro-MikA and *rac*-leptosphaerone B 5

Supplementary Figure S2: Analysis of covalently modified protein targets of *rac*-11-deoxy-MikA and *rac*-leptosphaerone B in *E. coli* K12 6

Supplementary Figure S3: *In vitro* reactivity screening of enones *rac*-leptosphaerone B and *rac*-11-deoxy-MikA with thiol-based nucleophiles and nucleophilic amino acids 7

Supplementary Figure S4: Whole proteome analyses of *S. aureus* NCTC 8325 treated with *rac*-11-deoxy-MikA, *rac*-dihydro-MikA or *rac*-leptosphaerone B vs. DMSO control 8

Table S1: List of chemicals used for biochemical experiments 41

Table S2: List of buffers used for biochemical experiments 42

Table S3: Bacterial cell lines used for this project. 43

Table S4: DIA-PASEF scan windows 53

5.2.1 ^{1}H NMR (400 MHz, $CDCl_3$) and ^{13}C NMR (101 MHz, $CDCl_3$) of compound 11 60

5.2.2 ^{1}H NMR (500 MHz, $CDCl_3$) and ^{13}C NMR (101 MHz, $CDCl_3$) of compound 6 61

5.2.3 ^{1}H NMR (400 MHz, $CDCl_3$) and ^{13}C NMR (101 MHz, $CDCl_3$) of compound 13 62

5.2.4 ^{1}H NMR (400 MHz, $CDCl_3$) and ^{13}C NMR (101 MHz, $CDCl_3$) of compound 14 63

5.2.5 ^{1}H NMR (500 MHz, $CDCl_3$) and ^{13}C NMR (101 MHz, $CDCl_3$) of compound 15 64

5.2.6 ^{1}H NMR (500 MHz, $CDCl_3$) and ^{13}C NMR (101 MHz, $CDCl_3$) of compound 9 65

5.2.7 ^{1}H NMR (500 MHz, $CDCl_3$) and ^{13}C NMR (75 MHz, $CDCl_3$) of compound 16 66

5.2.8 ^{1}H NMR (400 MHz, $CDCl_3$) and ^{13}C NMR (101 MHz, $CDCl_3$) of compound 17 67

5.2.9 ^{1}H NMR (500 MHz, $CDCl_3$) and ^{13}C NMR (75 MHz, $CDCl_3$) of *syn*-8 68

5.2.10 ^{1}H NMR (500 MHz, $CDCl_3$) and ^{13}C NMR (75 MHz, $CDCl_3$) of *anti*-8 69

5.2.11 ^{1}H NMR (400 MHz, $CDCl_3$) and ^{13}C NMR (101 MHz, $CDCl_3$) of compound 7 70

5.2.12 ^{1}H NMR (500 MHz, CD_3CN) of *rac*-leptosphaerone B (*rac*-4) 71

5.2.13 ^{1}H NMR (500 MHz, $CDCl_3$) and ^{13}C NMR (101 MHz, $CDCl_3$) of *syn*-35 72

5.2.14 ^{1}H NOESY NMR (500 MHz, $CDCl_3$) of *syn*-35...73

5.2.15 ^{1}H NMR (400 MHz, $CDCl_3$) and ^{13}C NMR (101 MHz, $CDCl_3$) of *rac*-dihydro-MikA (38) 74

5.2.16 ^{1}H NMR (500 MHz, $CDCl_3$) and ^{13}C NMR (101 MHz, $CDCl_3$) of compound 39 75

5.2.17 ^{1}H NMR (400 MHz, $CDCl_3$) and ^{13}C NMR (101 MHz, $CDCl_3$) of compound 42 76

5.2.18 ^{1}H NMR (400 MHz, $CDCl_3$) and ^{13}C NMR (101 MHz, $CDCl_3$) of compound 43 77

5.2.19 ^{1}H NMR (400 MHz, $CDCl_3$) and ^{13}C NMR (101 MHz, $CDCl_3$) of compound 20a...78

5.2.20 ^{1}H NMR (400 MHz, $CDCl_3$) and ^{13}C NMR (101 MHz, $CDCl_3$) of compound 20b...79

5.2.21 ^{1}H NMR (500 MHz, $CDCl_3$) and ^{13}C NMR (126 MHz, $CDCl_3$) of compound 44 80

5.2.22 ^{1}H NMR (400 MHz, $CDCl_3$) and ^{13}C NMR (101 MHz, $CDCl_3$) of *rac*-11-deoxy-MikA (46) 81

5.2.23 ^{1}H NMR (400 MHz, $CDCl_3$) and ^{13}C NMR (101 MHz, $CDCl_3$) of compound 24a...83

5.2.24 ^{1}H NMR (400 MHz, $CDCl_3$) and ^{13}C NMR (101 MHz, $CDCl_3$) of compound 24b...84

5.2.25 ^{1}H NMR (500 MHz, $CDCl_3$) of compound 26...85

5.2.26 ^{1}H NMR (500 MHz, $CDCl_3$) of compound 32...85

5.2.27 ^{1}H NMR (500 MHz, $CDCl_3$) and ^{13}C NMR (101 MHz, $CDCl_3$) of 2,3-dimethylphenyl acetate 86

5.2.28 ^{1}H NMR (400 MHz, $CDCl_3$) and ^{13}C NMR (101 MHz, $CDCl_3$) of compound 29b...87

5.2.29 ^{1}H NMR (400 MHz, $CDCl_3$) of compound 31a...88

5.2.30 ^{1}H NMR (500 MHz, $CDCl_3$) of compound 33...88

5.2.31 ^{1}H NMR (400 MHz, $CDCl_3$) of compound 34a...89

1 Supplementary Schemes, Figures and Tables

Supplementary Scheme S1: Enantioselective *syn*-dihydroxylation using a chiral sulfoximine auxiliary to direct the attack of osmium tetroxide to one side of the molecule through coordination.[1,2] However, the product decomposed during the thermolysis step.

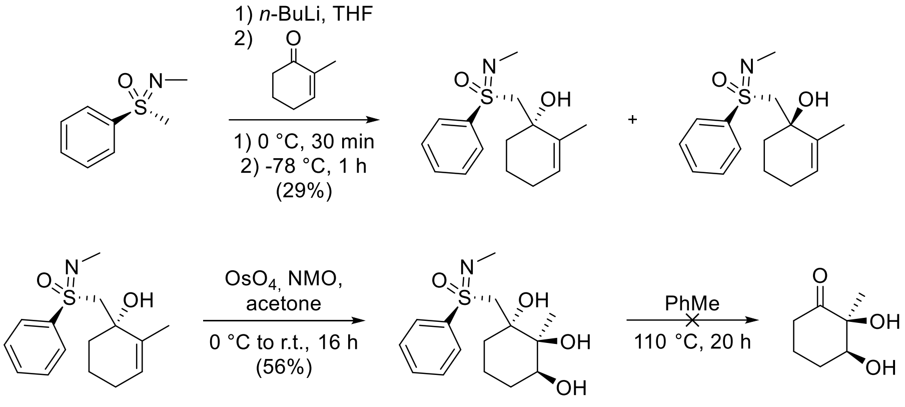

Supplementary Scheme S2: Synthesis of *N*-*tert*-butylbenzenesulfinimidoyl chloride.

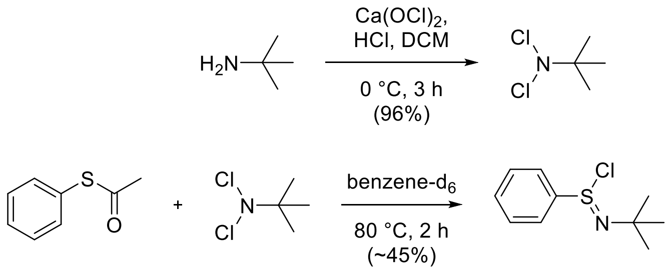

Supplementary Scheme S3: Mechanism of the dienolate alkylation reaction (green arrows) and a hypothetical mechanism for the concurring elimination of the acetonide protection group under alkaline conditions, leading to aromatization (red arrows).

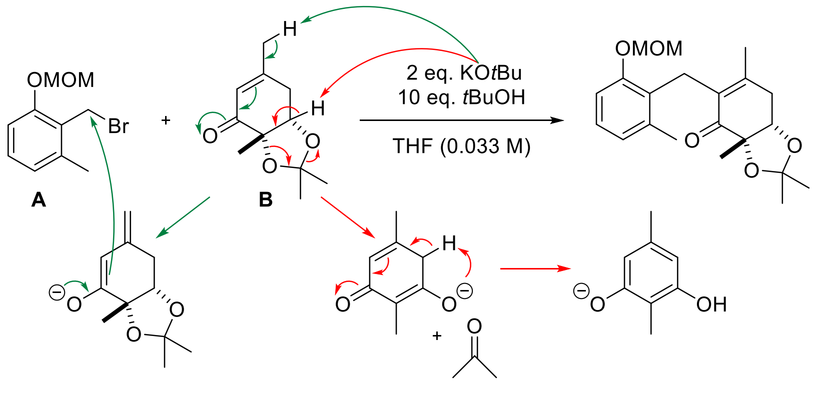

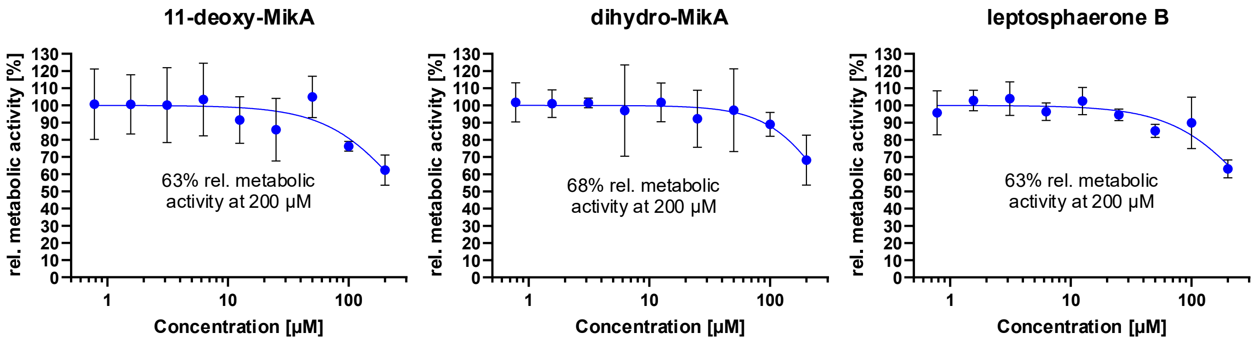

Supplementary Figure S1: Relative metabolic activity of HEK 293 cells determined through an MTT assay after 24 h treatment with *rac*-11-deoxy-MikA, *rac*-dihydro-MikA and *rac*-leptosphaerone B at concentrations up to 200 µM. Data represent mean ± std. of $n = 3$ replicates. IC_{50} values (concentration at which 50 % viability is reached) were determined using GraphPad Prism v10.0.1. Absorbance at 570 nm (formazan) and 630 nm (background) was measured and the background was subtracted, the absorption values of the replicates were normalized to the upper asymptote of a nonlinear fit of the data ([Inhibitor] vs. response – variable slope (four parameters)) for each compound tested, followed by re-fitting a nonlinear regression to the normalized data ([Inhibitor] vs. normalized response – variable slope) according to a protocol by Krebs *et al.*[3]

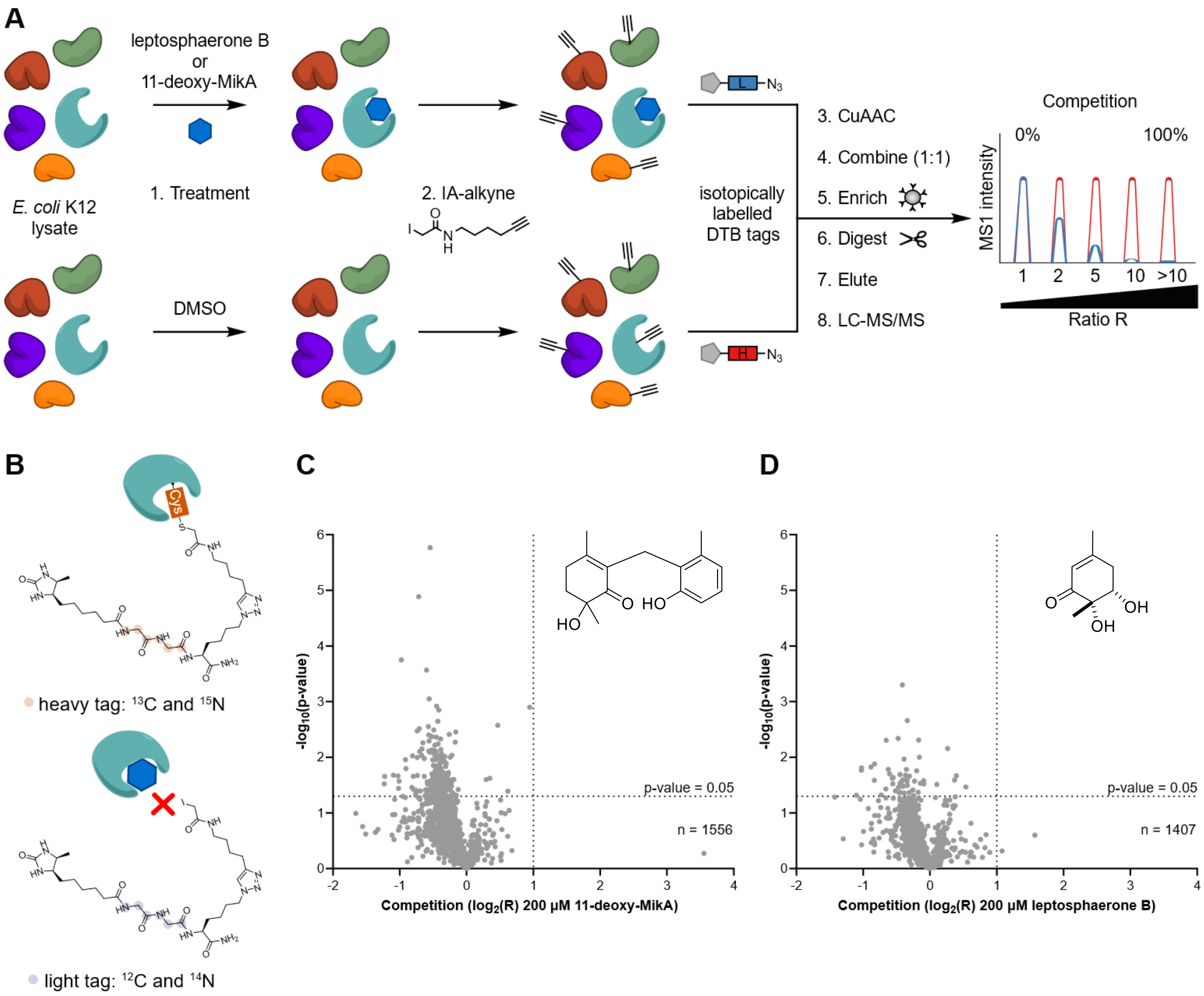

Supplementary Figure S2: Analysis of covalently modified protein targets of *rac*-11-deoxy-MikA and *rac*-leptosphaerone B in *E. coli* K12. (**A**) Schematic workflow for competitive, residue-specific proteomics using the isoDTB-ABPP platform.[4,5] Bacteria were lysed under non-denaturing conditions and the lysates were treated with either *rac*-11-deoxy-MikA or *rac*-leptosphaerone B (200 µM), or DMSO (1 %) for 1 h at room temperature. The treated samples were subsequently labeled with IA-alkyne (1 mM) and clicked to either a light (compound) or heavy (DMSO) isoDTB-tag. Samples were combined in a 1 : 1 ratio with their respective DMSO-control, enriched on streptavidin beads, tryptically digested, and analyzed via LC-MS/MS. The ratio between detected heavy- and light-tagged peptides indicates which cysteines are covalently engaged with the compounds. (B) Schematic representation of the competition between compound and IA-alkyne and structure of the isoDTB tags. (C and D) Volcano plots for *rac*-11-deoxy-MikA and *rac*-leptosphaerone B showing no covalently modified cysteine residues. The dashed lines indicate cut-offs at $-\log_{10}(p) = 1.3$ and $\log_2(R) = 1$ that were used as criteria for hit selection. Data represents $n = 4$ biological replicates.

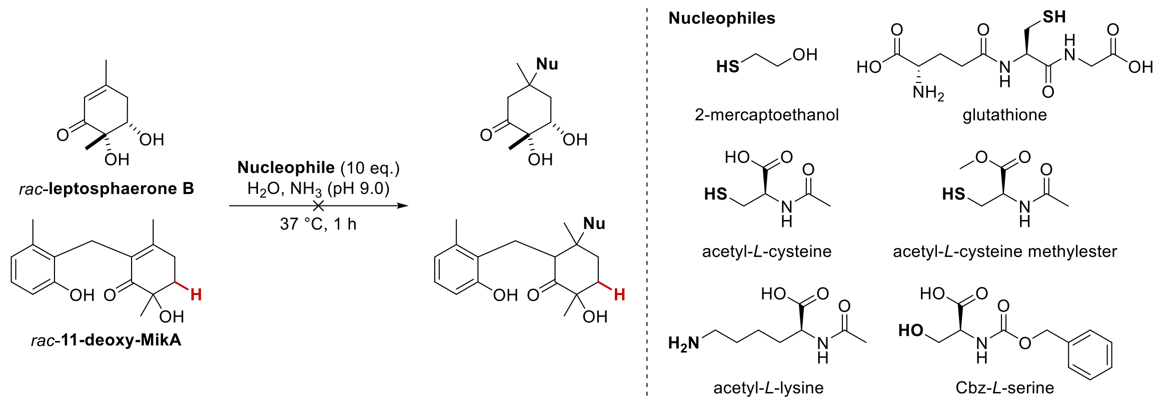

Supplementary Figure S3: *In vitro* reactivity screening of enones *rac*-leptosphaerone B and *rac*-11-deoxy-MikA with thiol-based nucleophiles and nucleophilic amino acids. The enones were incubated with 10-fold excess of nucleophiles at pH 9.0 at 37 °C for 1 h before the formation of adducts was examined *via* HPLC-MS. No adduct formation was observed for any combination of enone and nucleophile.

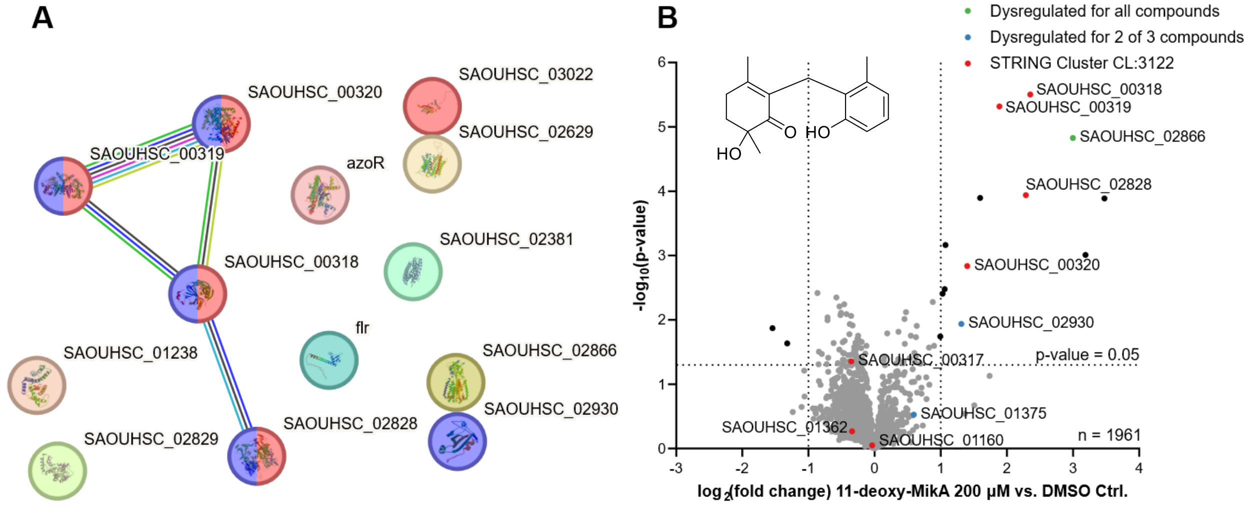

Local network cluster (STRING)

	cluster	description	gene count	strength	FDR
(red)	CL:3122	Mixed, incl. Xylene degradation, and Hexose phosphate transport	4 of 7	2.1	5.22E-05
(blue)	CL:3036	Mixed, incl. Degradation of aromatic compounds, and Glyoxalase/Bleomycin resistance protein/Dihydroxybiphenyl dioxygenase	5 of 58	1.28	0.001

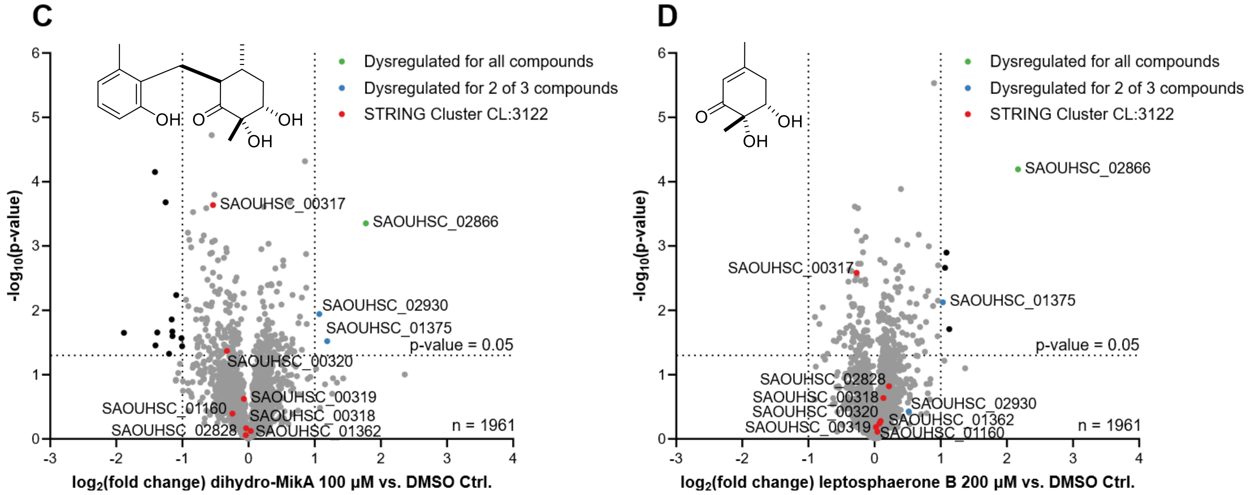

Supplementary Figure S4: Whole proteome analyses of *S. aureus* NCTC 8325 treated for 1 h at 37 °C with *rac*-11-deoxy-MikA (**A** and **B**, 200 µM), *rac*-dihydro-MikA (**C**, 100 µM) or *rac*-leptosphaerone B (**D**, 200 µM) versus DMSO control. (**A**) STRING GO-term analysis of significantly up-regulated proteins ($\log_2$(fold change)) >1, $p < 0.05$ of *rac*-11-deoxy-MikA-treated *S. aureus* NCTC 8325. For the analysis, the default STRING DB v.12.0 settings were used, and the required interaction score was set to high confidence (>0.7). The compound induces an up-regulation of the clusters CL:3122 (red) and CL:3036 (blue) associated with degradation of xylene and other aromatic compounds. (**B** to **D**) Volcano plots depicting the proteome regulation of *S. aureus* after the treatment. Threshold lines represent a $\log_2$ regulation of ± 1 and $-\log_{10}(p)$ of 1.3 (two-sided two-sample t-test, $n = 4$ replicates per treatment condition and $n = 3$ replicates for the DMSO control). Proteins up-regulated for all three compounds are marked green, proteins up-regulated for two of three compounds are marked blue, and proteins belonging to the STRING-Cluster CL:3122 are marked red. All other significantly dysregulated proteins are marked black. The only protein upregulated for all compounds is an MmpL efflux pump.

2 Chemical Synthesis Methods

2.1 General Considerations

All reactions, except otherwise noted or those employing aqueous reagents or media, were performed under Argon atmosphere (Ar 4.6, *Westfalen AG*) using standard *Schlenk* technique. Glassware was equipped with magnetic stirring and dried *in vacuo* using a heat gun. To transfer dry solvents or reagents, syringes were used, which were purged three times with Argon prior to use. Room temperature (r.t.) is defined as 20–25 °C.

All performed reactions were monitored by HPLC-MS, NMR spectroscopy or analytic thin-layer chromatography to examine the reaction progress. After purification by flash column chromatography, products were concentrated using a rotary evaporator. After purification by preparative HPLC, products were concentrated using a lyophilizer.

In this publication, the relative configuration of racemates is represented by straight lines (bold or hashed). The absolute configuration of enantiomerically pure or enriched compounds is represented by wedge-shaped lines (bold or hashed).

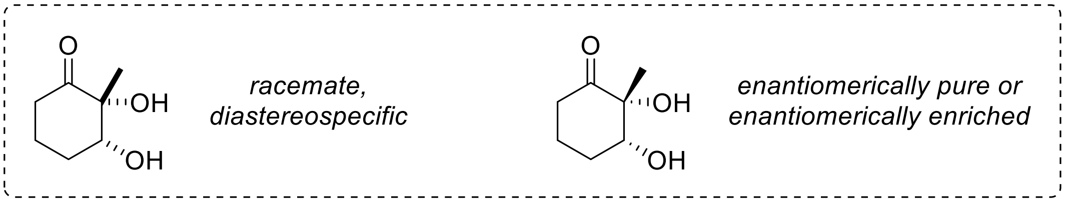

2.1.1 Solvents

For setting up reactions, dry organic solvents from commercial sources (*Sigma-Aldrich Co. LLC*, *Thermo Fisher Scientific Inc.*, *VWR International LLC*) were used. For extractions and purification purposes, acetone, dichloromethane (DCM), diethyl ether (Et_2O), ethyl acetate (EtOAc), n-pentane and methanol (MeOH) were used in technical grade from commercial sources. For analytical and preparative HPLC, acetonitrile (MeCN) and trifluoroacetic acid (TFA) of HPLC-grade purity (Fisher Scientific Inc.), as well as double distilled water (ddH_2O) was used. ddH_2O was prepared from deionized water using a *Sartorius* Arium® Pro ultrapure water system.

2.1.2 Reagents

All chemicals were purchased from commercial sources (*Sigma-Aldrich Co. LLC, BLD Pharmatech Ltd., Thermo Fisher Scientific Inc., Merck KGaA, ABCR GmbH & Co.KG, TCI Europe GmbH, Johnson Matthey Plc*) and were used without further purification unless otherwise stated.

- ***R*-(–)-*N*,*S*-dimethyl-*S*-phenylsulfoximine**

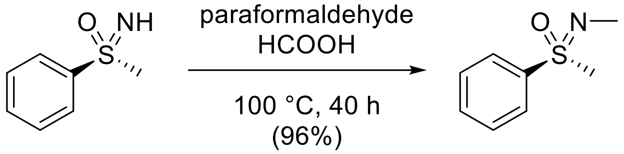

R-(–)-*N*,*S*-dimethyl-*S*-phenylsulfoximine was prepared from *R*-(–)-*S*-methyl-*S*-phenylsulfoximine through an Eschweiler-Clarke reaction according to a procedure of Shiner and Berks.[6]

- ***N*-*tert*-Butylbenzenesulfinimidoyl chloride**

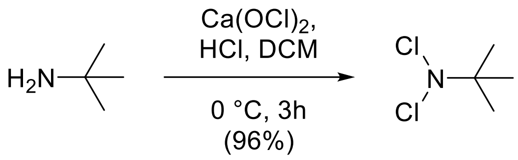

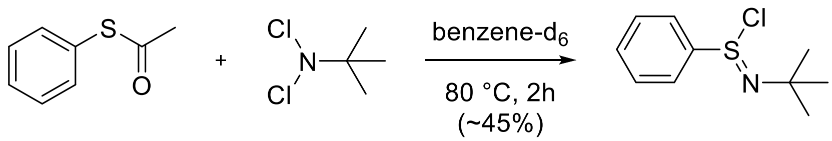

N-*tert*-Butylbenzenesulfinimidoyl chloride was synthesized from *tert*-butylamine and *S*-phenyl thioacetate according to a procedure of Matsuo *et al.*[7] The second step was performed in deuterated benzene to allow for determination of the yield and concentration of the crude product *via* NMR, as the reaction mixture was directly used in dehydrogenation reactions with enolates.

2.1.3 Chromatography

- **Thin layer chromatography (TLC)** was conducted using *Merck* silica gel 60 F_{254} plates. To visualize the spots of the different products, the TLC plates were exposed to UV light (wavelength λ = 254 nm) and stained with different staining solutions, followed by heating with a heat gun if necessary. Following staining reagents were used:
    - **$KMnO_4$-solution:** 1.5 g $KMnO_4$, 10 g K_2CO_3 and 0.125 g NaOH in 200 mL water
    - **CAM-solution:** 12 g ammonium molybdate, 0.5 g ceric ammonium molybdate and 15 mL H_2SO_4 in 235 mL water

- **Flash column chromatography** was performed with silica gel from *Merck* (SiO_2, 60 Å, 40–63 µm) as stationary phase and forced flow of the eluent stated in the experimental procedure.
- **Automated flash column chromatography** was performed on a flash column chromatography system (*Büchi* Reveleris X2) by employing silica gel from *Merck* (SiO_2, 60 Å, 40–63 µm) as stationary phase and forced flow of the eluent stated in the experimental procedure.
- **Preparative High-Performance Liquid Chromatography (prep-HPLC)** was performed using a *Waters* 2545 quaternary gradient module equipped with a fraction collector. The separation was performed on a *YMC* Triart C18 column (250 × 10 mm, 5 µm) with mobile phase A (0.1 % TFA in ddH_2O) and mobile phase B (0.1 % TFA in HPLC-grade acetonitrile) or mobile phase C (ddH_2O) and mobile phase D (HPLC-grade acetonitrile). The specific gradient used is defined in each procedure.

2.1.4 Analytical Data

NMR spectra were recorded on *Bruker* AV-HD400 and AV-HD500 spectrometers as solutions in $CDCl_3$ and *d*6-DMSO at 300 K. The signals refer to the residual proton signals of the deuterated solvent:

- $CDCl_3$: δ (^1H) = 7.26 ppm, δ (^{13}C) = 77.16 ppm
- d_6-DMSO: δ (^1H) = 2.50 ppm, δ (^{13}C) = 39.52 ppm
- CD_3CN: δ (^1H) = 1.94 ppm, δ (^{13}C) = 118.26, 1.32 ppm

All chemical shifts are reported as δ-values in parts per million (ppm). All coupling constants are reported in Hertz (Hz). For the characterization of the observed signal multiplicities the following abbreviations were used: s (singlet), d (doublet), t (triplet), q (quartet), dd (doublet of doublets), dt (doublet of triplets), td (triplet of doublets), tt (triplet of triplets), ddd (doublet of doublets of doublets), ddt (doublet of doublets of triplets), and m (multiplet). The NMR spectra were processed using MestReNova.

Mass spectrometry: Low-Resolution Mass Spectra (LRMS) for reaction controls were recorded on an MSQ™ Plus coupled to a DIONEX Ultimate 3000 HPLC system (both *Thermo Fisher scientific*). High-Resolution Mass Spectrometry (HRMS) was performed on an LTQ FT Ultra or a QExactive™ Plus mass spectrometer (both *Thermo Fisher scientific*), coupled

to a DIONEX Ultimate 3000 HPLC system (*Thermo Fisher scientific*). Electrospray ionization (ESI) or Atmospheric Pressure Chemical Ionization (APCI) was used as the ionization method. MS data were evaluated with THERMO Xcalibur 2.1 (*Thermo Fisher scientific*).

Gas Chromatography–Mass Spectrometry (GC–MS): Low-Resolution Mass Spectra (LRMS) with Electron Ionization (EI) were recorded on a GC-MS system by *Agilent*, consisting of an *Agilent* GC 7890B gas chromatograph and single quadrupole mass detector MS 5977A, equipped with a 7693A automatic liquid sampler with G4513A autoinjector. A HP-MS UI column (length: 30 m, internal diameter: 0.25 mm, film: 0.25 μm) was used for separation with a gradient from 60 to 300 °C. MS data was acquired and evaluated using *Agilent* MassHunter B.07.03.2129.

2.2 Detailed Experimental Procedures for All Compounds

2.2.1 1-(Methoxymethoxy)-2,3-dimethylbenzene (11)

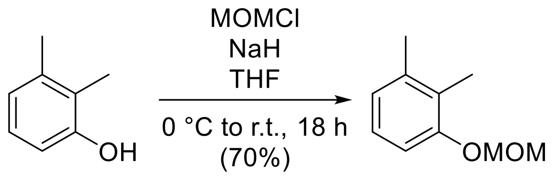

Sodium hydride (60 % in mineral oil, 4.0 g, 100 mmol, 2.0 eq.) was suspended in 80 mL of dry THF and cooled to 0 °C. A solution of 2,3-dimethylphenol (**12**, 6.11 g, 50.0 mmol, 1.0 eq.) in dry THF (175 mL) was added to the suspension via cannula, and the reaction mixture was allowed to warm to room temperature. Methoxymethyl chloride (5.71 mL, 75.0 mmol, 1.5 eq.) was added, and the mixture was stirred overnight at room temperature. The reaction was quenched by the addition of 250 mL of water, followed by vigorous stirring for 1 hour to hydrolyze remaining methoxymethyl chloride. The aqueous phase was extracted with ethyl acetate (3×). The combined organic layers were washed with brine, dried over anhydrous magnesium sulfate, filtered, and concentrated under reduced pressure. Purification by flash chromatography (pentane/ethyl acetate, 100:0 → 95:5) afforded **11** as a colorless liquid (5.80 g, 34.9 mmol, 70 %).

TLC (pentane:EtOAc = 95:5) R_f = 0.52 (UV)

^{1}H NMR (400 MHz, $CDCl_3$) δ [ppm] = 7.04 (t, J = 7.9 Hz, 1H), 6.92 (d, J = 8.2 Hz, 1H), 6.84 (d, J = 7.5 Hz, 1H), 5.19 (s, 2H), 3.50 (s, 3H), 2.28 (s, 3H), 2.19 (s, 3H).

^{13}C NMR (101 MHz, $CDCl_3$) δ [ppm] = 155.33, 138.21, 126.10, 126.01, 123.54, 111.97, 94.92, 56.13, 20.28, 11.93.

HRMS (ESI-LTQ-FT, positive) m/z: $[M+H]^+$ calcd. for $C_{10}H_{15}O_2^+$: 167.10666; found: 167.10667.

2.2.2 2-(Bromomethyl)-1-(methoxymethoxy)-3-methylbenzene (6)

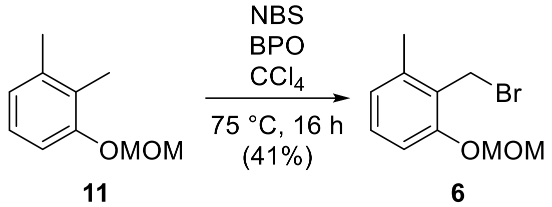

1-(Methoxymethoxy)-2,3-dimethylbenzene (**11**, 658 mg, 3.96 mmol, 1.0 eq.) and *N*-bromo-succinimide (705 mg, 3.96 mmol, 1.0 eq.) were dissolved in carbon tetrachloride (25 mL) under argon atmosphere. Benzoyl peroxide (75 % in water, 128 mg, 0.396 mmol, 0.1 eq.) was added, and the reaction mixture was heated to 75 °C for 16 h. After cooling to room temperature, the mixture was filtered, and the solvent was removed under reduced pressure. Purification by flash column chromatography (pentane/DCM, 100:0 → 95:5) afforded **6** as a colorless oil (501 mg, 1.64 mmol, 80 % purity, 41 % yield).

TLC (pentane:DCM = 75:25) R_f = 0.30 (UV)

Note: NMR analysis showed 80 % product, 4 % starting material, and 16 % dibrominated side product.

^{1}H NMR (500 MHz, $CDCl_3$) δ [ppm] = 7.16 (t, J = 8.0 Hz, 1H), 6.95 (d, J = 8.3 Hz, 1H), 6.84 (d, J = 7.5 Hz, 1H), 5.26 (s, 2H), 4.67 (s, 2H), 3.52 (s, 3H), 2.41 (s, 3H).

^{13}C NMR (101 MHz, $CDCl_3$) δ [ppm] = 155.23, 138.97, 129.45, 125.18, 123.84, 111.82, 94.30, 56.23, 26.09, 18.84.

HRMS (APCI-LTQ-FT, positive) m/z: $[M-Br]^+$ calcd. for $C_{10}H_{13}O_2^+$: 165.09101; found: 165.09102.

2.2.3 2-Iodocyclohex-2-en-1-one (13)

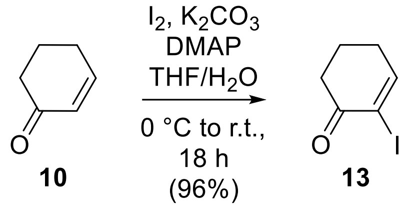

2-Cyclohexen-1-one (**10**, 21.0 g, 208 mmol, 1.0 eq.) was dissolved in a mixture of tetrahydrofuran and water (1 L, *v*/*v* = 1:1) and cooled to 0 °C. Iodine (106 g, 416 mmol, 2.0 eq.), potassium carbonate (34.5 g, 250 mmol, 1.2 eq.), and 4-(dimethylamino)pyridine (5.08 g, 41.6 mmol, 0.2 eq.) were added, and the reaction mixture was stirred at 0 °C for 10 minutes. The ice bath was then removed and the mixture stirred at room temperature overnight (16 h). Ethyl acetate was added and the layers were separated. The aqueous phase was extracted with ethyl acetate (3×). The combined organic layers were washed thoroughly with aqueous sodium thiosulfate solution, 1 M HCl, and brine. The organic phase was dried over anhydrous magnesium sulfate, filtered, and concentrated under reduced pressure. Purification by flash column chromatography (hexane/ethyl acetate, 100:0 → 90:10) afforded **13** as a pale yellow solid (44.5 g, 200 mmol, 96 %). Note: The product is sensitive to prolonged exposure to air, light or room temperature and was therefore stored in the dark at –20 °C under argon atmosphere.

TLC (pentane:EtOAc = 9:1) R_f = 0.30 (UV)

^{1}H NMR (400 MHz, $CDCl_3$) δ [ppm] = 7.77 (t, J = 4.4 Hz, 1H), 2.70–2.63 (m, 2H), 2.44 (td, J = 6.0, 4.4 Hz, 2H), 2.13–2.05 (m, 2H).

^{13}C NMR (101 MHz, $CDCl_3$) δ [ppm] = 192.31, 159.51, 104.04, 37.40, 30.08, 23.00.

HRMS (ESI-LTQ-FT, positive) m/z: $[M+H]^+$ calcd. for $C_6H_8IO^+$: 222.96143; found: 222.96143.

2.2.4 2-Methylcyclohex-2-en-1-one (14)

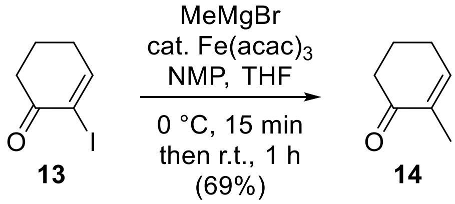

3-Iodo-2-cyclohexen-1-one (**13**, 20.0 g, 90.0 mmol, 1.0 eq.) and iron(III) acetylacetonate (1.59 g, 4.50 mmol, 0.05 eq.) were dissolved in dry tetrahydrofuran (180 mL), and the resulting solution was cooled to 0 °C. *N*-Methyl-2-pyrrolidone (78.7 mL, 810 mmol, 9.0 eq.) was added, followed by dropwise addition of methylmagnesium bromide (3 M in THF, 39.0 mL, 117 mmol, 1.3 eq.). The reaction mixture was stirred at 0 °C for 15 minutes and subsequently warmed to room temperature and stirred for an additional hour. The reaction was quenched by the addition of 1 M HCl and extracted with diethyl ether (3×). The combined organic phases were washed successively with saturated aqueous sodium bicarbonate solution, water, and brine. The organic layer was dried over anhydrous magnesium sulfate and concentrated under reduced pressure (not below 350 mbar at 40 °C or 100 mbar at 20 °C, as the product is volatile!). Purification by flash column chromatography (pentane/diethyl ether, 9:1) afforded **14** as an orange oil (6.80 g, 61.7 mmol, 69 %).

TLC (pentane:Et_2O = 9:1) R_f = 0.28 (UV)

^{1}H NMR (400 MHz, $CDCl_3$) δ [ppm] = 6.81–6.67 (m, 1H), 2.46–2.39 (m, 2H), 2.35–2.28 (m, 2H), 2.02–1.94 (m, 2H), 1.80–1.74 (m, 3H).

^{13}C NMR (101 MHz, $CDCl_3$) δ [ppm] = 200.19, 145.74, 135.86, 38.47, 26.17, 23.44, 16.14.

HRMS (APCI-LTQ-FT, positive) m/z: $[M+H]^+$ calcd. for $C_7H_{11}O^+$: 111.08044; found: 111.08042.

2.2.5 *syn*-2,3-Dihydroxy-2-methylcyclohexan-1-one (15)

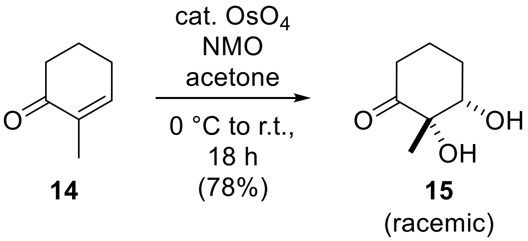

2-Methylcyclohex-2-en-1-one (**14**, 6.84 g, 62.1 mmol, 1.0 eq.) was dissolved in dry acetone (120 mL) under an argon atmosphere. The solution was degassed by the freeze-pump-thaw method for 5 min and subsequently warmed to 0 °C in an ice bath. Osmium tetroxide (4 % in water, 5.29 mL, 0.841 mmol, 1.35 mol%) and *N*-methylmorpholine *N*-oxide (50 % in water, 14 mL, 68 mmol, 1.1 eq.) were added to the reaction mixture. The ice bath was removed, and the mixture was stirred overnight while warming to room temperature. The reaction was quenched by the addition of two spatula tips of solid sodium thiosulfate. After stirring for 5 min, anhydrous magnesium sulfate was added, and the mixture was filtered through a short plug of celite and washed with acetone. The solvent was removed under reduced pressure. Purification by flash column chromatography (DCM/methanol, 100:0 → 95:5) afforded **15** as an orange oil (6.94 g, 48.1 mmol, 77 %).

TLC (DCM:MeOH = 9:1) R_f = 0.39 ($KMnO_4$)

^{1}H NMR (500 MHz, $CDCl_3$) δ [ppm] = 4.26 (s, 1H), 4.05 (t, J = 2.8 Hz, 1H), 2.89 (s, 1H), 2.62–2.51 (m, 1H), 2.50–2.43 (m, 1H), 2.23–2.03 (m, 2H), 2.00–1.86 (m, 2H), 1.39 (s, 3H).

^{13}C NMR (101 MHz, $CDCl_3$) δ [ppm] = 213.71, 78.79, 76.99, 37.06, 27.87, 23.59, 21.55.

HRMS (ESI-LTQ-FT, positive) m/z: $[M+H]^+$ calcd. for $C_7H_{13}O_3^+$: 145.08592; found: 145.08592.

2.2.6 2,2,3a-Trimethyltetrahydrobenzo[d][1,3]dioxol-4(3aH)-one (9)

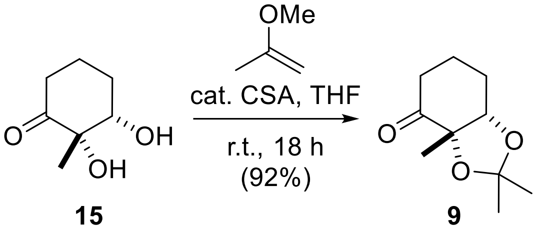

syn-2,3-Dihydroxy-2-methylcyclohexan-1-one (**15**, 6.94 g, 48.1 mmol, 1.0 eq.) was dissolved in dry THF (350 mL). 2-Methoxypropene (35.8 g, 45.9 mL, 481 mmol, 10.0 eq.) and 10-camphorsulfonic acid (228 mg, 0.963 mmol, 0.02 eq.) were added, and the mixture was stirred at room temperature for 18 h. The reaction was then quenched with water (300 mL), and the mixture was extracted with ethyl acetate (3×). The combined organic layers were dried over anhydrous magnesium sulfate, filtered, and concentrated under reduced pressure. Purification by flash column chromatography (DCM/methanol, 100:0 → 95:5) afforded **9** as a yellow oil (8.15 g, 44.2 mmol, 92 %).

TLC (DCM:MeOH = 95:5) R_f = 0.40 ($KMnO_4$)

^{1}H NMR (500 MHz, $CDCl_3$) δ [ppm] = 4.17 (td, J = 2.8, 1.2 Hz, 1H), 2.50–2.43 (m, 1H), 2.41–2.32 (m, 1H), 2.27–2.19 (m, 1H), 2.08–1.85 (m, 3H), 1.39 (s, 3H), 1.38 (d, J = 1.9 Hz, 6H).

^{13}C NMR (101 MHz, $CDCl_3$) δ [ppm] = 211.81, 108.48, 83.94, 82.28, 39.79, 27.17, 26.92, 26.18, 20.65, 19.97.

HRMS (ESI-LTQ-FT, positive) m/z: $[M+H]^+$ calcd. for $C_{10}H_{17}O_3^+$: 185.11722; found: 185.11726.

2.2.7 2,2,3a-Trimethyl-5-(phenylselanyl)tetrahydrobenzo[d][1,3]dioxol-4(3aH)-one (16)

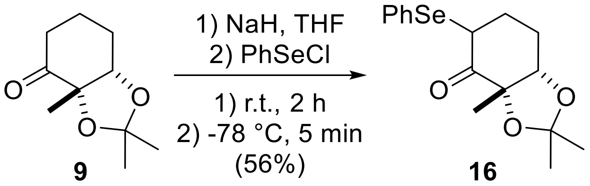

Sodium hydride (60 % in mineral oil, 1.60 g, 40.1 mmol, 1.0 eq.) was suspended in dry THF (80 mL) under argon. A solution of compound **9** (7.39 g, 40.1 mmol, 1.0 eq.) in dry THF (160 mL) was added dropwise to the NaH suspension at room temperature. The reaction mixture was stirred for 2 h, then cooled to –78 °C. Phenylselenyl chloride (8.62 g, 44.1 mmol, 1.1 eq.) was added in one portion. The mixture was allowed to warm to room temperature, then poured into saturated aqueous NH_4Cl (300 mL) and diluted with water until the precipitate dissolved. The layers were separated, and the aqueous phase was extracted with diethyl ether (3×). The combined organic phases were washed with brine, dried over anhydrous magnesium sulfate, filtered, and concentrated under reduced pressure. Purification by flash column chromatography (pentane/ethyl acetate, 100:0 → 91:9) afforded **16** as a yellow oil (7.84 g, 22.6 mmol, 56 %).

TLC (pentane:EtOAc = 9:1) R_f = 0.33 (UV)

^{1}H NMR (500 MHz, $CDCl_3$) δ [ppm] = 7.56–7.49 (m, 2H), 7.36–7.25 (m, 3H), 4.19 (t, J = 2.7 Hz, 1H), 3.97 (dt, J = 4.3, 1.9 Hz, 1H), 2.59 (tt, J = 13.9, 4.3 Hz, 1H), 2.29–2.20 (m, 1H), 2.18–2.11 (m, 1H), 2.06–2.00 (m, 1H), 1.71 (s, 3H), 1.33 (d, J = 40.5 Hz, 7H).

^{13}C NMR (75 MHz, $CDCl_3$) δ [ppm] = 207.68, 135.01, 129.47, 128.81, 128.10, 108.53, 82.88, 80.95, 48.03, 27.27, 26.96, 24.88, 24.80, 22.53.

HRMS (ESI-LTQ-FT, positive) m/z: $[M+H]^+$ calcd. for $C_{16}H_{21}O_3Se^+$: 341.06504; found: 341.06504.

2.2.8 2,2,3a-Trimethyl-7,7a-dihydrobenzo[d][1,3]dioxol-4(3aH)-one (17)

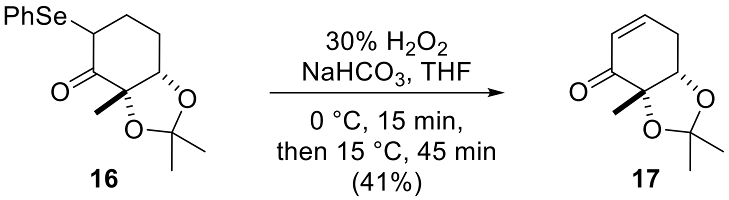

A solution of compound **16** (357 mg, 1.00 mmol, 1.0 eq.) in THF (20 mL) was cooled to 0 °C. Sodium bicarbonate (420 mg, 5.00 mmol, 5.0 eq.) and hydrogen peroxide (35 % in water, 0.43 mL, 5.00 mmol, 5.0 eq.) were added, and the mixture was stirred at 0 °C for 15 min. The reaction was then allowed to warm to room temperature using a water bath and stirred for another 30 min. A 1:1 mixture of saturated aqueous sodium bicarbonate and sodium thiosulfate solution was added, followed by additional water until the precipitate dissolved. The mixture was extracted with diethyl ether (4×). The combined organic layers were washed with brine, dried over anhydrous magnesium sulfate, filtered, and concentrated under reduced pressure. Purification by flash column chromatography (pentane/diethyl ether, 100:0 → 50:50) afforded **17** as a pale-yellow oil (74 mg, 0.406 mmol, 41 %), which crystallized overnight.

TLC (pentane:Et_2O = 1:1) R_f = 0.28 ($KMnO_4$)

^{1}H NMR (400 MHz, $CDCl_3$) δ [ppm] = 6.86–6.74 (m, 1H), 6.08 (ddd, J = 10.2, 2.9, 1.2 Hz, 1H), 4.24 (dt, J = 4.6, 1.6 Hz, 1H), 2.89 (ddt, J = 20.4, 5.2, 1.3 Hz, 1H), 2.73 (ddt, J = 20.4, 4.5, 2.8 Hz, 1H), 1.39 (d, J = 0.8 Hz, 3H), 1.36 (s, 3H), 1.30 (d, J = 0.8 Hz, 3H).

^{13}C NMR (101 MHz, $CDCl_3$) δ [ppm] = 200.01, 145.34, 127.77, 108.35, 80.55, 78.57, 27.48, 27.38, 26.78, 19.15.

HRMS (ESI-LTQ-FT, positive) m/z: $[M+H]^+$ calcd. for $C_{10}H_{15}O_3^+$: 183.10157; found: 183.10158.

2.2.9 2,2,3a,6-Tetramethyltetrahydrobenzo[d][1,3]dioxol-4(3aH)-one (8)

Procedure A

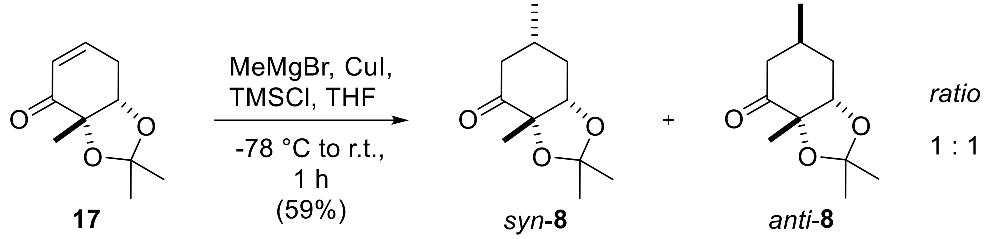

Compound **17** (179 mg, 0.909 mmol, 1.0 eq.), copper(I) iodide (24.2 mg, 0.127 mmol, 0.14 eq.), and trimethylchlorosilane (198 mg, 1.82 mmol, 2.0 eq.) were dissolved in diethyl ether (11 mL). The solution was cooled to –78 °C, and methylmagnesium bromide (3 M in Et_2O, 0.667 mL, 2.00 mmol, 2.2 eq.) was added dropwise. The mixture was allowed to warm to room temperature and stirred for 1 hour. The reaction was quenched by the dropwise addition of saturated aqueous NH_4Cl. The phases were separated, and the aqueous layer was extracted with diethyl ether (3×). The combined organic phases were washed with brine, dried over anhydrous magnesium sulfate, filtered, and concentrated under reduced pressure. Purification by flash column chromatography (pentane/diethyl ether, 80:20 → 70:30 → 60:40) afforded the two diastereomers *syn*-**8** (54.5 mg, 0.275 mmol, 30 %) and *anti*-**8** (51.5 mg, 0.26 mmol, 29 %) as white crystalline solids, which slowly turned yellow after storage at –20 °C.

Procedure B

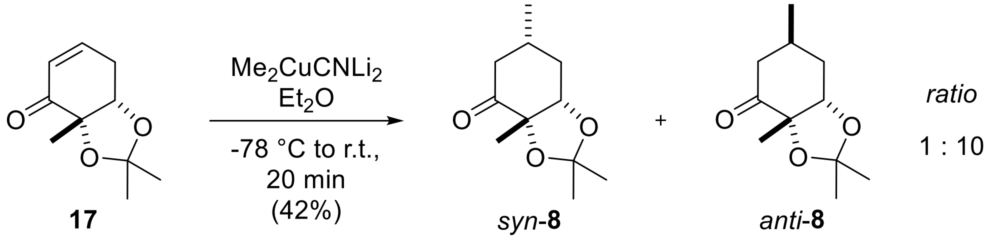

To a stirred solution of copper(I) cyanide (35.9 mg, 0.401 mmol, 1.2 eq.) in dry diethyl ether (10.0 mL), methyllithium (1.6 M in Et_2O, 459 µL, 0.735 mmol, 2.2 eq.) was added dropwise at –78 °C under an argon atmosphere. The mixture was stirred for 10 minutes at 0 °C and then cooled again to –78 °C. A solution of compound **17** (76.1 mg, 0.334 mmol, 1.0 eq.) in diethyl ether (7.5 mL) was added dropwise, and the resulting mixture was stirred for 20 minutes at 0 °C, during which the bright yellow color gradually faded. The reaction was quenched by addition of saturated aqueous NH_4Cl (10 mL) at –78 °C. The layers were separated, and the aqueous phase was extracted with Et_2O (3×). The combined organic phases were washed with brine, dried over anhydrous magnesium sulfate, filtered, and concentrated under reduced pressure. The crude product was purified by flash column chromatography (pentane/diethyl ether, 8:2 → 7:3 → 6:4 → 5:5) to afford 28 mg of a 1:10 mixture of the two diastereomers *syn*-**8** (2.5 mg, 0.025 mmol, 4 %) and *anti*-**8** (25.4 mg, 0.25 mmol, 38 %) as a colorless oil.

HRMS (ESI-LTQ-FT, positive) m/z: $[M+H]^+$ calcd. for $C_{11}H_{19}O_3^+$: 199.13287; found: 199.13289.

syn-**8**

TLC (pentane:Et_2O = 60:40) R_f = 0.27 ($KMnO_4$)

^{1}H NMR (500 MHz, $CDCl_3$) δ [ppm] = 4.25 (t, J = 4.4 Hz, 1H), 2.67 (dd, J = 15.0, 6.1 Hz, 1H), 2.44–2.34 (m, 1H), 2.25 (ddd, J = 15.1, 5.2, 1.7 Hz, 1H), 2.20 (dt, J = 15.1, 4.6 Hz, 1H), 1.97–1.89 (m, 1H), 1.44 (d, J = 0.7 Hz, 3H), 1.43 (s, 3H), 1.37 (d, J = 0.8 Hz, 3H), 1.13 (d, J = 7.2 Hz, 3H).

^{13}C NMR (75 MHz, $CDCl_3$) δ [ppm] = 211.34, 108.92, 83.80, 82.94, 45.35, 32.81, 29.54, 27.54, 26.62, 21.54, 21.38.

anti-**8**

TLC (pentane:Et_2O = 60:40) R_f = 0.32 ($KMnO_4$)

^{1}H NMR (500 MHz, $CDCl_3$) δ [ppm] = 4.14 (t, J = 2.8 Hz, 1H), 2.42 (dt, J = 14.0, 3.0 Hz, 1H), 2.34–2.26 (m, 1H), 2.26–2.20 (m, 1H), 2.12 (dd, J = 13.9, 13.1 Hz, 1H), 1.63 (ddd, J = 15.1, 12.1, 2.9 Hz, 1H), 1.38 (s, 6H), 1.37 (s, 3H), 1.06 (d, J = 6.5 Hz, 3H).

^{13}C NMR (75 MHz, $CDCl_3$) δ [ppm] = 211.29, 108.56, 83.28, 81.92, 47.94, 34.70, 28.11, 27.19, 26.91, 21.75, 19.96.

2.2.10 2,2,3a,6-Tetramethyl-5-(phenylselanyl)tetrahydrobenzo[d][1,3]dioxol-4(3aH)-one (18)

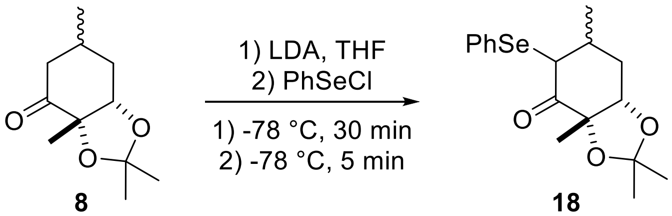

Diisopropylamine (58.3 mg, 0.576 mmol, 1.2 eq.) was dissolved in dry THF (1.5 mL), and the solution was cooled to –78 °C. *n*-Butyllithium (1.8 M in hexane, 0.320 mL, 0.576 mmol, 1.2 eq.) was added dropwise, and the mixture was stirred for 10 minutes to generate lithium diisopropylamide (LDA).

A solution of a 1:1 mixture of *syn*- and *anti*-**8** (95.2 mg, 0.48 mmol, 1.0 eq.) in THF (1.5 mL) was then added dropwise to the cold LDA solution. After stirring for 30 minutes at –78 °C, a solution of phenylselenyl chloride (93.8 mg, 0.48 mmol, 1.0 eq.) in THF (1.0 mL) was added in one portion. After 5 minutes, the mixture was quenched with saturated aqueous NH_4Cl (5 mL), and water was added until the precipitate dissolved. The mixture was extracted with diethyl ether (3×). The combined organic layers were washed with brine, dried over anhydrous magnesium sulfate, filtered, and concentrated under reduced pressure. Purification by flash column chromatography (pentane/ethyl acetate, 80:20 → 0:100) afforded a mixture of educt **8** and product **18** as a yellow oil (100 mg), which was directly taken forward in the next step.

TLC (pentane:Et_2O = 8:2) R_f = 0.53 ($KMnO_4$).

2.2.11 2,2,3a,6-Tetramethyl-7,7a-dihydrobenzo[d][1,3]dioxol-4(3aH)-one (7)

Procedure A

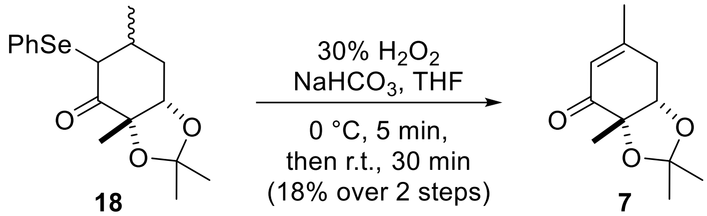

The mixture of educt **8** and product **18** from the previous step (100 mg, 0.19 mmol, 1.0 eq.) was dissolved in dry THF (5 mL) and cooled to 0 °C. Sodium bicarbonate (119 mg, 1.42 mmol, 7.5 eq.) and hydrogen peroxide (35% in water, 161 mg, 1.42 mmol, 7.5 eq.) were added sequentially. The mixture was stirred for 5 minutes at 0 °C, then warmed to room temperature using a water bath and stirred for an additional 30 minutes. A 1:1 mixture of saturated aqueous $NaHCO_3$ and $Na_2S_2O_3$ was added, followed by water until the precipitate dissolved. The aqueous layer was extracted with diethyl ether (4×). The combined organic layers were washed with brine, dried over anhydrous magnesium sulfate, filtered, and evaporated under reduced pressure. Purification by flash column chromatography (pentane/diethyl ether, 100:0 → 75:25 → 50:50) afforded **7** (16.7 mg, 0.0851 mmol, 18% yield over two steps).

Procedure B

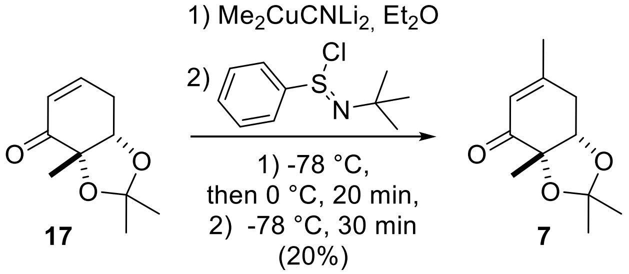

To a stirred solution of copper(I) cyanide (66.3 mg, 0.74 mmol, 1.2 eq.) in dry diethyl ether (10 mL), methyllithium (1.6 M in Et_2O, 1.36 mmol, 718 µL, 2.2 eq.) was added dropwise at –78 °C under an argon atmosphere. The mixture was stirred for 10 minutes at 0 °C, then cooled again to –78 °C. A solution of compound **17** (125 mg, 0.617 mmol, 1.0 eq.) in diethyl ether (4 mL) was added dropwise. The resulting mixture was stirred for 20 minutes at 0 °C, during which the initially bright yellow color faded. Subsequently, a solution of *N-tert*-butylbenzenesulfinimidoyl chloride (0.37 M in benzene, 5.00 mL, 1.85 mmol, 3.0 eq.) was added at –78 °C, and the reaction mixture was stirred for 30 minutes at this temperature.

The reaction was quenched by the dropwise addition of 10% aqueous NH_4OH in saturated NH_4Cl (25 mL) at –78 °C. The layers were separated, and the aqueous phase was extracted with diethyl ether (3×). The combined organic layers were washed with water and brine, dried over anhydrous magnesium sulfate, filtered, and concentrated under reduced pressure. The crude product was purified by flash column chromatography (pentane/diethyl ether, 8:2 → 6:4 → 4:6 → 0:10) to afford **7** (24 mg, 0.122 mmol, 20 %).

TLC (pentane:Et_2O = 6:4) R_f = 0.21 ($KMnO_4$)

^{1}H NMR (400 MHz, $CDCl_3$) δ [ppm] = 5.94–5.91 (m, 1H), 4.21 (dd, J = 4.2, 1.9 Hz, 1H), 2.82–2.72 (m, 1H), 2.72–2.64 (m, 1H), 2.01 (s, 3H), 1.39 (s, 4H), 1.35 (s, 3H), 1.30 (s, 3H).

^{13}C NMR (101 MHz, $CDCl_3$) δ [ppm] = 199.58, 157.34, 124.62, 108.36, 79.70, 78.55, 32.35, 27.41, 26.86, 24.45, 19.29.

2.2.12 *syn*-5,6-Dihydroxy-3,6-dimethylcyclohex-2-en-1-one (*rac*-leptosphaerone B, *rac*-4)

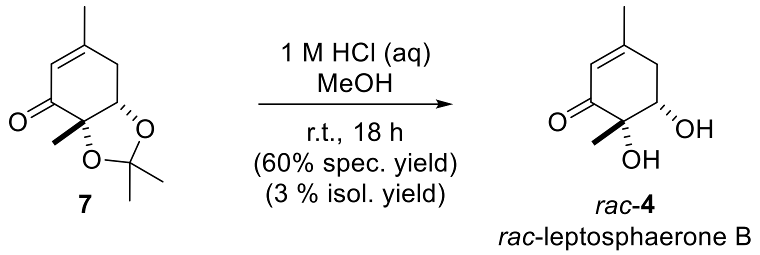

7 *rac*-**4**
rac-leptosphaerone B

A solution of compound **7** (23.9 mg, 0.122 mmol, 1.0 eq.) in methanol (2.0 mL) was treated with 1 M aqueous HCl (2.0 mL, 2.00 mmol, 16.4 eq.) and stirred at room temperature for 18 h. The reaction mixture was concentrated under reduced pressure until almost dry. Magnesium sulfate was added, and the residue was washed with DCM/MeOH. The filtrate was evaporated and subjected to flash column chromatography (DCM/MeOH, 100:0 → 97:3 → 95:5 → 90:10), yielding 18 mg of still impure *rac*-leptosphaerone B (*rac*-**4**, 60 % NMR yield). Preparative HPLC (2–40 % MeCN, 27 min) gave pure *rac*-leptosphaerone B (0.60 mg, 3.84 µmol, 3 %).

^{1}H NMR (500 MHz, CD_3CN) δ [ppm] = 5.84–5.81 (m, 1H), 3.97 (dt, J = 4.1, 2.3 Hz, 1H), 3.79 (s, 1H), 3.11 (t, J = 1.9 Hz, 1H), 2.76–2.68 (m, 1H), 2.46 (dd, J = 19.3, 2.5 Hz, 1H), 1.94 (s, 3H), 1.22 (s, 3H).

Note: *The amount of product was too small to acquire a ^{13}C NMR spectrum.*

HRMS (ESI-LTQ-FT, positive) m/z: $[M+H]^+$ calcd. for $C_8H_{13}O_3^+$: 157.08592; found: 157.08595.

2.2.13 5-(2-(Methoxymethoxy)-6-methylbenzyl)-2,2,3a,6-tetramethyltetrahydrobenzo[d][1,3]dioxol-4(3aH)-one (*syn*-35)

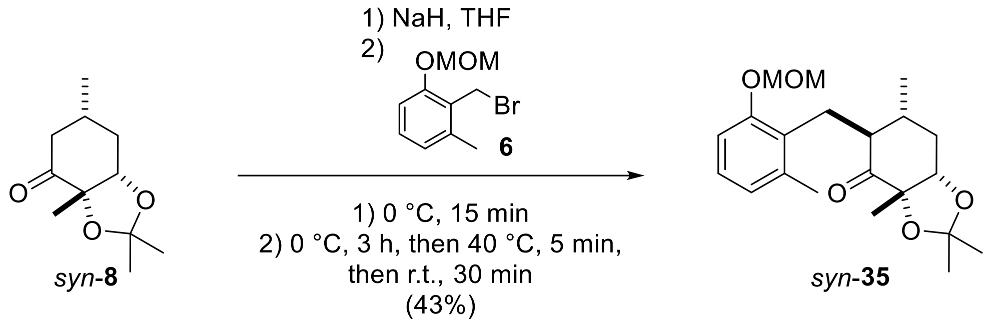

A solution of *syn*-**8** (14.0 mg, 0.0706 mmol, 1.0 eq.) in THF (0.5 mL) was cooled to 0 °C, and sodium hydride (60 % in mineral oil, 6.5 mg, 0.162 mmol, 2.3 eq.) was added. After 15 min, compound **6** (25.3 mg, 0.0918 mmol, 1.3 eq.) in 0.2 mL THF was added dropwise. The reaction mixture was stirred at 0 °C for 2 h, then additional 5.7 eq. of NaH (60 % in mineral oil, 16.1 mg, 0.403 mmol) were added, and stirring continued for 1 h at 0 °C. The reaction mixture was then allowed to warm to room temperature, briefly heated to 30–40 °C using a heat gun, and stirred for an additional 30 min at room temperature. The reaction was quenched with saturated aqueous NH_4Cl and extracted with diethyl ether (3×). The combined organic phases were washed with water and brine, dried over anhydrous magnesium sulfate, and concentrated under reduced pressure. Purification by flash column chromatography (pentane/ethyl acetate, 100:0 → 95:5 → 92.5:7.5) afforded a single diastereomer of the product (*syn*-**35**) as a colorless oil (11.0 mg, 0.0303 mmol, 43 %).

TLC (pentane/EtOAc, 80:20) R_f = 0.36 (CAM)

^{1}H NMR (500 MHz, $CDCl_3$) δ [ppm] = 7.05 (t, J = 7.9 Hz, 1H), 6.92 (d, J = 8.3 Hz, 1H), 6.81 (d, J = 7.5 Hz, 1H), 5.17 (s, 2H), 4.31 (t, J = 5.2 Hz, 1H), 3.47 (s, 3H), 2.97 (dd, J = 13.6, 7.7 Hz, 1H), 2.86 (dd, J = 13.6, 6.8 Hz, 1H), 2.72 (q, J = 7.4 Hz, 1H), 2.34 (s, 3H), 2.17 (dt, J = 14.7, 5.0 Hz, 1H), 2.04 (ddd, J = 13.0, 9.5, 6.0 Hz, 1H), 1.63 (ddd, J = 14.7, 6.8, 5.7 Hz, 1H), 1.51 (s, 3H), 1.42 (s, 3H), 1.39 (s, 3H), 1.07 (d, J = 6.8 Hz, 3H).

^{13}C NMR (101 MHz, $CDCl_3$) δ [ppm] = 212.22, 155.78, 138.16, 127.41, 127.13, 124.18, 111.51, 109.98, 94.79, 83.12, 82.52, 56.28, 53.03, 32.93, 32.75, 27.72, 27.56, 26.52, 22.70, 22.35, 20.05.

HRMS (ESI-LTQ-FT, positive) m/z: $[M+H]^+$ calcd. for $C_{21}H_{31}O_5^+$: 363.21660; found: 363.21678.

2.2.14 2,3-Dihydroxy-6-(2-hydroxy-6-methylbenzyl)-2,5-dimethylcyclohexan-1-one (*rac*-dihydro-MikA, 38)

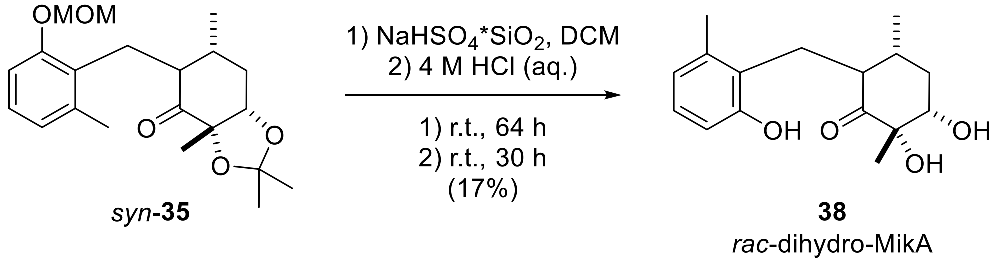

To a solution of *syn*-**35** (14.0 mg, 38.6 µmol, 1.0 eq.) in DCM (1.0 mL) was added silica-supported sodium bisulfate ($NaHSO_4 \cdot SiO_2$, 30.0 mg, 66.2 µmol, 1.71 eq.), which had prior been kept in an oven at 120 °C for at least 48 h. The reaction mixture was stirred at room temperature for 64 h. Aqueous HCl (4 M, 1 mL) was added, and the mixture was stirred for 30 h. The catalyst was filtered off and washed with DCM (2 × 5 mL). The combined filtrates were concentrated under reduced pressure. Purification by preparative HPLC (20–98 % MeCN with 0.1 % TFA, 23 min) gave racemic dihydro-MikA (**38**, 1.8 mg, 6.47 µmol, 17 %).

^{1}H NMR (400 MHz, CD_3CN) δ [ppm] = 6.98 (t, J = 7.7 Hz, 1H), 6.76 (d, J = 7.4 Hz, 1H), 6.61 (dd, J = 26.7, 8.1 Hz, 1H), 3.51 (dd, J = 11.6, 4.8 Hz, 1H), 2.78 (dd, J = 17.1, 5.6 Hz, 1H), 2.70 (dd, J = 17.1, 2.0 Hz, 1H), 2.23 (s, 3H), 1.89 (ddd, J = 11.4, 5.7, 1.9 Hz, 1H), 1.60 (dt, J = 12.3, 4.3 Hz, 1H), 1.45–1.33 (m, 1H), 1.37 (s, 3H), 1.24–1.08 (m, 1H), 0.90 (d, J = 6.5 Hz, 3H).

^{13}C NMR (101 MHz, CD_3CN) δ [ppm] = 195.45, 152.63, 138.39, 127.38, 123.14, 115.34, 99.22, 76.01, 72.23, 39.53, 39.07, 28.73, 21.76, 19.76, 19.10, 18.16.

HRMS (ESI-LTQ-FT, negative) m/z: $[M-H]^-$ calcd. for $C_{16}H_{21}O_4^-$: 277.14453; found: 277.14446.

2.2.15 3,6-Dimethylcyclohex-2-en-1-one (39)

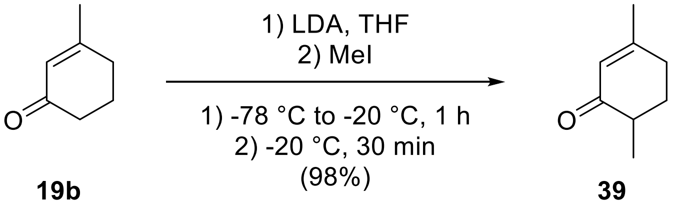

Diisopropylamine (3.39 mL, 24.0 mmol, 1.2 eq.) was dissolved in dry THF (25 mL) and cooled to –78 °C. *n*-Butyllithium (2.5 M in hexanes, 9.6 mL, 24.0 mmol, 1.2 eq.) was added dropwise, and the mixture was stirred for 10 min to form lithium diisopropylamide.

3-Methyl-2-cyclohexen-1-one (**19b**, 2.28 mL, 20.0 mmol, 1.0 eq.) in dry THF (15 mL) was added dropwise, and the mixture was allowed to warm to –20 °C over 1 h. Iodomethane (1.37 mL, 22.0 mmol, 1.1 eq.) in dry THF (8 mL) was added dropwise, and the mixture was stirred for 30 min at –20 °C. The reaction was quenched with saturated aqueous NH_4Cl. The layers were separated, and the aqueous phase was extracted with diethyl ether (3×). The combined organic phases were washed with brine, dried over anhydrous magnesium sulfate, filtered, and concentrated under reduced pressure to yield the crude product **39** in sufficient purity for the next step (2.42 g, 19.5 mmol, 98 %).

^{1}H NMR (500 MHz, $CDCl_3$) δ [ppm] = 5.85–5.82 (m, 1H), 2.39–2.21 (m, 3H), 2.04 (m, 1H), 1.93 (s, 3H), 1.76–1.64 (m, 1H), 1.12 (d, J = 6.8 Hz, 3H).

^{13}C NMR (101 MHz, $CDCl_3$) δ [ppm] = 202.25, 161.63, 126.26, 40.55, 30.82, 30.71, 24.29, 15.19.

2.2.16 6-Hydroxy-3,6-dimethylcyclohex-2-en-1-one (42)

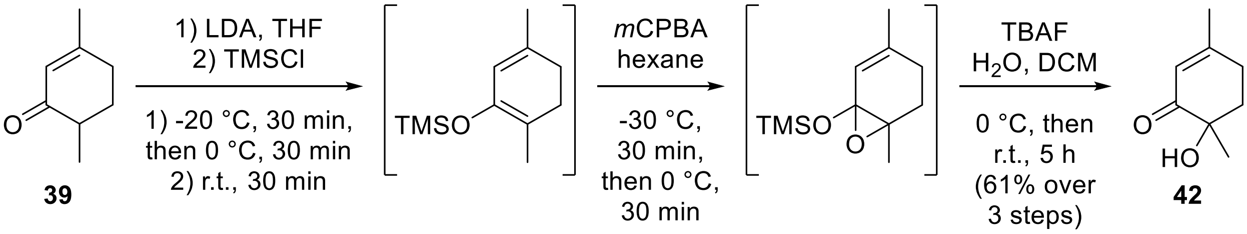

A solution of diisopropylamine (3.37 mL, 2.43 g, 24.1 mmol, 1.26 eq.) in anhydrous THF (75 mL) was cooled to –20 °C, and *n*-butyllithium (2.5 M in hexanes, 8.63 mL, 21.6 mmol, 1.13 eq.) was added dropwise. The mixture was stirred for 10 min to form lithium diisopropylamide.

A solution of compound **39** (2.372 g, 19.1 mmol, 1.0 eq.) in THF (10 mL) was added dropwise, and the mixture was stirred for 30 min at –20 °C, followed by an additional 30 min at 0 °C. Chlorotrimethylsilane (4.85 mL, 38.2 mmol, 2.0 eq.) was added in one portion, and the reaction mixture was stirred for 30 min at 25 °C. The reaction was quenched with cold saturated aqueous $NaHCO_3$ (50 mL) and extracted with hexanes (3×). The organic phase was dried over anhydrous magnesium sulfate, filtered, and concentrated under reduced pressure to afford the crude enol silyl ether.

m-Chloroperoxybenzoic acid (70–75 %, 3.63 g, 21 mmol, 1.1 eq.) in hexane (170 mL) was stirred for 20 min at 25 °C and then cooled to –30 °C. The crude enol silyl ether was dissolved in hexanes (20 mL) and slowly added over 5 min. The mixture was stirred for 30 min at –30 °C and 30 min at 0 °C. The reaction mixture was filtered, and the volatiles were removed under reduced pressure to give the crude epoxide.

The residue was dissolved in DCM (90 mL), cooled to 0 °C, and treated with TBAF (1 M in THF, 38.2 mL, 38.2 mmol, 2.0 eq.) and a few drops of water. The mixture was stirred at 25 °C for 5 h, then quenched with saturated aqueous $NaHCO_3$ (100 mL) and extracted with diethyl ether (3×). The combined organic extracts were dried over anhydrous magnesium sulfate, filtered, and concentrated under reduced pressure. Purification by flash column chromatography (pentane/ethyl acetate, 9:1) afforded compound **42** as a colorless oil (1.62 g, 11.6 mmol, 61 %).

TLC (pentane/EtOAc, 7:3) R_f = 0.25 ($KMnO_4$)

^{1}H NMR (400 MHz, $CDCl_3$) δ [ppm] = 5.90–5.86 (m, 1H), 3.68 (s, 1H), 2.46–2.36 (m, 2H), 2.09 (ddd, J = 13.2, 5.2, 2.5 Hz, 1H), 2.02 (ddd, J = 11.0, 7.2, 5.5 Hz, 1H), 1.97 (s, 3H), 1.29 (d, J = 0.5 Hz, 3H).

^{13}C NMR (101 MHz, $CDCl_3$) δ [ppm] = 202.39, 163.29, 123.28, 72.48, 35.55, 30.44, 24.50, 24.39, 24.37.

2.2.17 6-(Methoxymethoxy)-3,6-dimethylcyclohex-2-en-1-one (43)

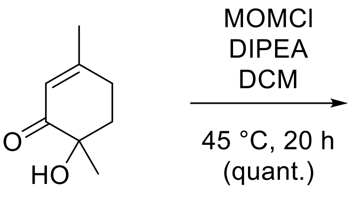

42 **43**

To a solution of compound **42** (1.50 g, 10.7 mmol, 1.0 eq.) in dry DCM (50 mL) were added chloromethyl methyl ether (1.679 mL, 21 mmol, 2.0 eq.) and *N*,*N*-diisopropylethylamine (9.15 mL, 52.5 mmol, 5.0 eq.), and the reaction mixture was heated to 45 °C for 20 h. The next day, 1 M NaOH was added, and stirring was continued for 1 h to degrade remaining chloromethyl methyl ether. The mixture was cooled to room temperature, the phases were separated, and the aqueous layer was extracted with DCM (2×). The combined organic phases were washed with a mixture of saturated aqueous NH_4Cl and 1 M HCl, followed by brine, and subsequently dried over anhydrous magnesium sulfate, filtered, and concentrated under reduced pressure to afford the pure product as a yellow oil (1.97 g, 10.7 mmol, 100 %).

^{1}H NMR (400 MHz, $CDCl_3$) δ [ppm] = 5.86–5.79 (m, 1H), 4.74–4.65 (m, 2H), 3.31 (s, 3H), 2.61–2.50 (m, 1H), 2.32–2.22 (m, 1H), 2.27–2.16 (m, 1H), 1.95–1.94 (m, 3H), 1.97–1.90 (m, 1H), 1.34 (s, 3H).

^{13}C NMR (101 MHz, $CDCl_3$) δ [ppm] = 198.30, 161.52, 124.97, 92.16, 76.50, 55.67, 35.68, 29.04, 24.16, 20.75.

2.2.18 General Procedure A for the Reaction of Dienolates with Benzyl Bromides

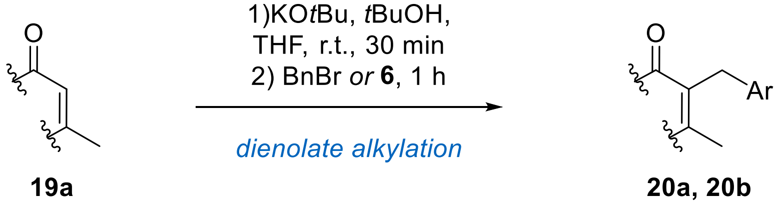

19a **43** **20a, 20b** **44**

Potassium *tert*-butoxide (2.0 eq.) was dissolved in anhydrous THF (20 mL per mmol of enone), and *tert*-butanol (10 eq.) was added. A solution of a 3-methylcyclohex-2-en-1-one derivative (**19a** or **43**, 1.0 eq.) in THF (0.5 mL per mmol) was added, and the mixture was stirred for 30 min at room temperature. A solution of a benzyl bromide derivative (benzyl bromide or **1**, 1.0 eq.) in THF (10 mL) was added to the reaction mixture, and stirring was

continued for 1 h at room temperature. The mixture was extracted with ethyl acetate (3×). The combined organic layers were washed with brine, dried over anhydrous magnesium sulfate, filtered, and concentrated under reduced pressure.

2.2.18.1 (1*S*,5*R*)-3-Benzyl-4,6,6-trimethylbicyclo[3.1.1]hept-3-en-2-one (20a)

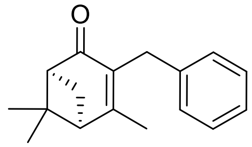

20a (81%)

(1*S*)-(–)-Verbenone (**19a**) and benzyl bromide were used and the experiment was performed on a 1.00 mmol scale according to general procedure A (2.2.18). Purification by automated flash column chromatography (pentane/ethyl acetate, 100:0 → 95:5) afforded the product (195 mg, 0.811 mmol, 81 %) as a colorless oil

^{1}H NMR (400 MHz, $CDCl_3$) δ [ppm] = 7.28–7.19 (m, 4H), 7.17–7.11 (m, 1H), 3.70 (d, J = 14.6 Hz, 1H), 3.58 (d, J = 14.6 Hz, 1H), 2.77 (dt, J = 8.9, 5.4 Hz, 1H), 2.71 (dd, J = 6.7, 5.4 Hz, 1H), 2.41 (dd, J = 6.7, 5.4 Hz, 1H), 2.05 (d, J = 8.9 Hz, 1H), 2.03 (s, 3H), 1.46 (s, 3H), 0.91 (s, 3H).

^{13}C NMR (101 MHz, $CDCl_3$) δ [ppm] = 203.20, 163.28, 140.78, 130.01, 128.63, 128.41, 125.81, 57.56, 54.29, 50.73, 40.74, 29.58, 26.86, 22.03, 20.99.

2.2.18.2 (1*S*,5*R*)-3-(2-(Methoxymethoxy)-6-methylbenzyl)-4,6,6-trimethylbicyclo[3.1.1]hept-3-en-2-one (20b)

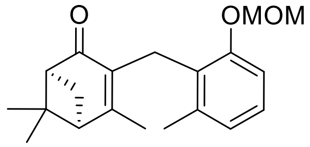

20b (52%)

(1*S*)-(–)-Verbenone (**19a**) and 2-(bromomethyl)-1-(methoxymethoxy)-3-methylbenzene (**6**) were used and the experiment was performed on a 1.00 mmol scale according to general procedure A (2.2.18). Purification by automated flash column chromatography (pentane/ethyl acetate, 100:0 → 95:5) afforded the product (203 mg, 0.516 mmol, 52 %) as a colorless oil.

TLC (pentane/EtOAc, 95:5) R_f = 0.26

^{1}H NMR (400 MHz, $CDCl_3$) δ [ppm] = 7.03 (t, J = 7.9 Hz, 1H), 6.92 (dd, J = 8.3, 1.2 Hz, 1H), 6.80 (d, J = 7.5 Hz, 1H), 5.16 (d, J = 6.6 Hz, 1H), 5.13 (d, J = 6.7 Hz, 1H), 3.76 (d, J = 15.3 Hz, 1H), 3.63 (dd, J = 15.2, 1.3 Hz, 1H), 3.46 (s, 3H), 2.72–2.63 (m, 2H), 2.35 (s, 3H), 2.34–2.31 (m, 1H), 2.00 (d, J = 8.4 Hz, 1H), 1.86 (d, J = 0.9 Hz, 3H), 1.44 (s, 3H), 0.97 (s, 3H).

^{13}C NMR (101 MHz, $CDCl_3$) δ [ppm] = 202.92, 162.47, 155.78, 138.77, 129.43, 128.29, 126.58, 124.14, 111.73, 94.97, 57.75, 56.16, 54.47, 51.48, 40.06, 26.76, 22.14, 21.50, 20.57, 20.51.

2.2.18.3 6-(Methoxymethoxy)-2-(2-(methoxymethoxy)-6-methylbenzyl)-3,6-dimethylcyclohex-2-en-1-one (44)

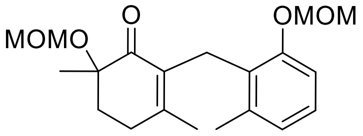

44 (quant.)

6-(Methoxymethoxy)-3,6-dimethylcyclohex-2-en-1-one (**43**) and 2-(bromomethyl)-1-(methoxymethoxy)-3-methylbenzene (**6**) were used and the experiment was performed on a 3.00 mmol scale according to general procedure A (2.2.18). Purification by flash column chromatography (pentane/ethyl acetate, 9:1 → 8:2 → 7:3) afforded compound **44** (1.04 g, 2.99 mmol, 100 %) as a yellow oil.

TLC (pentane/EtOAc, 8:2) R_f = 0.31

^{1}H NMR (500 MHz, $CDCl_3$) δ [ppm] = 7.01 (t, J = 7.9 Hz, 1H), 6.88 (dd, J = 8.4, 1.3 Hz, 1H), 6.77 (dt, J = 7.5, 1.0 Hz, 1H), 5.11 (s, 2H), 4.62 (dd, J = 31.8, 7.3 Hz, 2H), 3.75 (dd, J = 44.8, 15.2 Hz, 2H), 3.44 (s, 3H), 3.29 (s, 3H), 2.58–2.49 (m, 1H), 2.30 (s, 3H), 2.29–2.20 (m, 2H), 1.93–1.87 (m, 1H), 1.81 (s, 3H), 1.34 (s, 3H).

^{13}C NMR (126 MHz, $CDCl_3$) δ [ppm] = 197.39, 155.55, 154.65, 138.63, 132.74, 128.30, 126.45, 124.13, 111.68, 94.77, 92.17, 77.10, 56.08, 55.48, 34.78, 30.75, 23.28, 21.46, 21.19, 20.51.

2.2.19 *rac*-6-Hydroxy-2-(2-hydroxy-6-methylbenzyl)-3,6-dimethylcyclohex-2-en-1-one (*rac*-11-deoxy-MikA, 46)

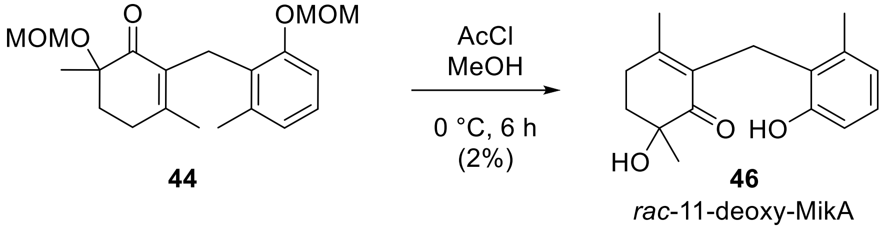

A solution of compound **44** (348 mg, 1.00 mmol, 1.0 eq.) in methanol (2 mL) was cooled to 0 °C, and acetyl chloride (6.0 µL, 0.10 mmol, 0.10 eq.) was added. The mixture was stirred at 0 °C for 6 h. Ethyl acetate and saturated aqueous $NaHCO_3$ were added, and the mixture was thoroughly mixed. The phases were separated, and the aqueous layer was extracted with ethyl acetate (3×). The combined organic layers were washed with brine, dried over anhydrous magnesium sulfate, filtered, and concentrated under reduced pressure. The residue was purified by preparative HPLC (30–98 % MeCN, 30 min) to afford *rac*-11-deoxy-MikA (**46**, 4.3 mg, 0.0165 mmol, 2 %).

^{1}H NMR (400 MHz, DMSO) δ [ppm] = 9.05 (s, 1H), 6.75 (t, J = 7.8 Hz, 1H), 6.55 (d, J = 7.9 Hz, 1H), 6.18 (d, J = 7.6 Hz, 1H), 4.92 (s, 1H), 3.46 (d, J = 16.1 Hz, 1H), 3.34 (d, J = 16.1 Hz, 1H), 2.41 (d, J = 6.1 Hz, 1H), 2.05 (s, 3H), 1.92–1.79 (m, 2H), 1.74 (s, 3H), 1.13 (s, 3H).

^{13}C NMR (101 MHz, DMSO) δ [ppm] = 199.93, 156.84, 154.92, 139.04, 130.41, 125.47, 121.86, 116.95, 112.21, 71.12, 35.53, 30.04, 27.84, 24.01, 21.07, 11.07.

HRMS (ESI-Orbitrap, positive) m/z: $[M+H]^+$ calcd. for $C_{16}H_{21}O_3^+$ 261.14852; found 261.14822.

2.2.20 General Procedure B for the Epoxidation of Enones

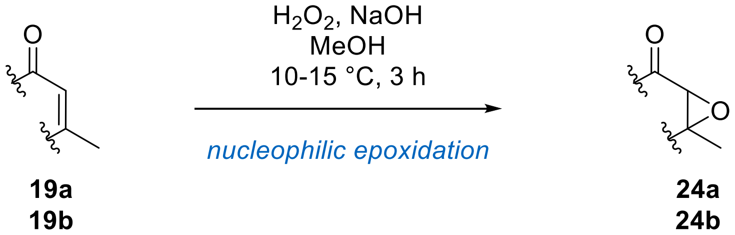

A 3-methylcyclohex-2-en-1-one derivative (**19a** or **19b**, 5.00 mmol, 1.0 eq.) was dissolved in methanol (8 mL) and cooled to 10 °C. Hydrogen peroxide (35 % in water, 2.0 mL, 20 mmol,

4.0 eq.) was added, followed by an aqueous sodium hydroxide solution (6 M, 467 µL, 2.80 mmol, 0.56 eq.). The mixture was stirred for 2 h at 12–15 °C, diluted with water (10 mL), and extracted with ethyl acetate (4×). The combined organic layers were washed with water (2×), dried over anhydrous magnesium sulfate, filtered, and concentrated under reduced pressure to afford α,β-epoxy ketones **24a** and **24b** as colorless oils in sufficient purity without further purification.

2.2.20.1 (1*R*,6*S*)-2,7,7-Trimethyl-3-oxatricyclo[4.1.1.0^{2,4}]octan-5-one (24a)

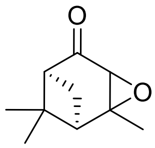

24a (78%)

(–)-Verbenone (**19a**, 833 µg, 5.00 mmol) was epoxidized according to general procedure B (2.2.20) to afford verbenone epoxide (**24a**) as a colorless oil (649 mg, 3.90 mmol, 78 %).

^{1}H NMR (400 MHz, $CDCl_3$) δ [ppm] = 3.17 (d, J = 1.7 Hz, 1H), 2.44 (td, J = 5.7, 1.8 Hz, 1H), 2.32 (t, J = 5.9 Hz, 1H), 2.30–2.23 (m, 1H), 2.09–2.05 (m, 1H), 1.51 (s, 3H), 1.43 (s, 3H), 1.27 (d, J = 6.9 Hz, 1H), 1.02 (s, 3H).

^{13}C NMR (101 MHz, $CDCl_3$) δ [ppm] = 206.37, 60.11, 59.15, 56.71, 50.00, 45.90, 26.67, 21.91, 21.78, 21.03.

2.2.20.2 6-methyl-7-oxabicyclo[4.1.0]heptan-2-one (24b)

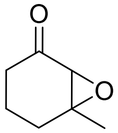

24b (61%)

3-Methylcyclohex-2-en-1-one (**19b**, 567 µg, 5.00 mmol) was epoxidized according to general procedure B (2.2.20) to afford 6-methyl-7-oxabicyclo[4.1.0]heptan-2-one (**24b**) as a colorless oil (386 mg, 3.06 mmol, 61 %).

^{1}H NMR (400 MHz, $CDCl_3$) δ [ppm] = 3.07 (s, 1H), 2.52–2.44 (m, 1H), 2.16–1.81 (m, 4H), 1.68–1.59 (m, 1H), 1.45 (s, 3H).

^{13}C NMR (101 MHz, $CDCl_3$) δ [ppm] = 206.97, 62.59, 62.13, 35.82, 28.53, 22.35, 17.32.

2.2.21 General Procedure C for the Iodination of β-Methylcyclohexenones with PIFA

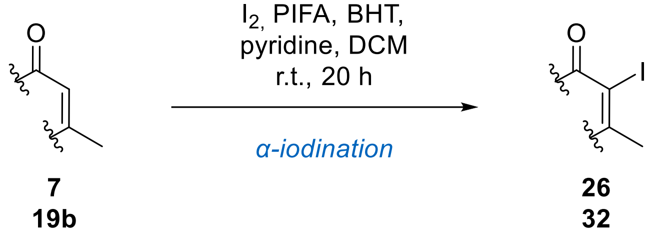

Iodine (1.0 eq.) and pyridine (1.5 eq.) were dissolved in DCM. Phenyliodine(III) bis(trifluoroacetate) (1.0 eq.) was added, and the mixture was stirred for 15 min at 20 °C in the dark. Butylated hydroxytoluene (0.05 eq.) and a solution of the enone (1.0 eq.) in DCM were then added. The reaction mixture was stirred for 22 h at room temperature. The reaction was quenched with saturated aqueous $Na_2S_2O_3$ and extracted with ethyl acetate (3×). The combined organic layers were washed with brine, dried over anhydrous magnesium sulfate, filtered, and concentrated under reduced pressure.

2.2.21.1 5-Iodo-2,2,3a,6-tetramethyl-7,7a-dihydrobenzo[d][1,3]dioxol-4(3aH)-one (26)

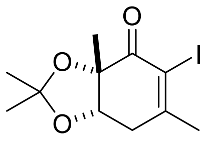

26 (10%)

Compound **7** was used and the experiment was performed on a 150 µmol scale in 1.2 mL DCM according to General Procedure C (2.2.21). Purification by flash column chromatography (hexanes/ethyl acetate, 75:25 → 70:30) afforded compound **26** (5 mg, 15.5 µmol, 10 %).

^{1}H NMR (500 MHz, $CDCl_3$) δ [ppm] = 4.15 (dd, J = 4.4, 1.6 Hz, 1H), 3.02 (dd, J = 19.6, 1.6 Hz, 1H), 2.90–2.81 (m, 1H), 2.29 (d, J = 1.1 Hz, 3H), 1.39 (s, 6H), 1.26 (s, 3H).

2.2.21.2 2-Iodo-3-methylcyclohex-2-en-1-one (32)

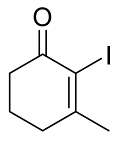

32 (96%)

Compound **19b** was used and the experiment was performed on a 1.0 mmol scale in 3.75 mL DCM according to General Procedure C (2.2.21). Purification by flash column chromatography (pentane/ethyl acetate, 100:0 → 95:5 → 92.5:7.5 → 75:25) afforded compound **32** (227 mg, 0.962 µmol, 96 %).

TLC (pentane/EtOAc, 9:1) $R_f = 0.3$

^{1}H NMR (500 MHz, $CDCl_3$) δ [ppm] = 2.60 (dd, J = 7.7, 6.7 Hz, 2H), 2.55 (t, J = 6.0 Hz, 2H), 2.25 (s, 3H), 1.98 (dt, J = 13.3, 6.1 Hz, 2H).

2.2.22 2,3-Dimethylphenyl acetate

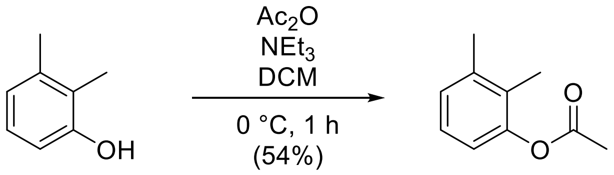

12

2,3-Dimethylphenol (**12**, 5.00 g, 40.1 mmol, 1.0 eq.) was dissolved in DCM (12 mL) and cooled to 0 °C. Triethylamine (11.3 mL, 80.2 mmol, 2.0 eq.) was added, followed by dropwise addition of acetic anhydride (initially 5.7 mL, 61.2 mmol, 1.5 eq.). After 20 min, TLC indicated incomplete conversion, and an additional 5.0 mL acetic anhydride (53.5 mmol, total 2.8 eq.) was added. The reaction mixture was stirred for a total of 60 min at 0 °C until TLC showed full conversion. Ice-cold water was added, and the aqueous layer was extracted with DCM. The combined organic layers were washed with brine, dried over anhydrous magnesium sulfate, filtered, and concentrated under reduced pressure to give a brown oil and colorless crystals. The mixture was diluted with pentane, filtered, and the crystals were washed with additional pentane. The filtrate was concentrated to give a clear brown oil. Purification by automated flash column chromatography (hexane/ethyl acetate, 100:0 → 9:1) afforded 2,3-dimethylphenyl acetate as a pale orange oil (3.58 g, 21.8 mmol, 54 %).

TLC (pentane:EtOAc = 9:1) R_f = 0.69 (UV)

^{1}H NMR (500 MHz, $CDCl_3$) δ [ppm] = 7.10 (t, J = 7.7 Hz, 1H), 7.05 (d, J = 7.5 Hz, 1H), 6.86 (d, J = 8.0 Hz, 1H), 2.33 (s, 3H), 2.30 (s, 3H), 2.08 (s, 3H).

^{13}C NMR (101 MHz, $CDCl_3$) δ [ppm] = 169.62, 149.37, 138.65, 128.79, 127.66, 126.20, 119.54, 20.97, 20.19, 12.53.

HRMS (ESI-LTQ-FT, positive) m/z: $[M+H]^+$ calcd. for $C_{10}H_{13}O_2^+$: 165.09101; found 165.09108.

2.2.23 2-(Bromomethyl)-3-methylphenyl acetate (29b)

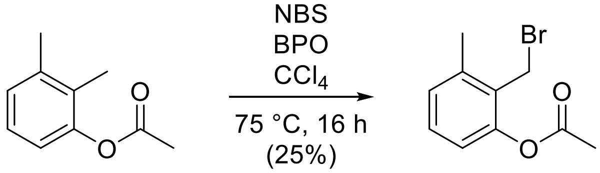

29b

A solution of 2,3-dimethylphenyl acetate (1.64 g, 10.0 mmol, 1.0 eq.) and *N*-bromosuccinimide (1.78 g, 10.0 mmol, 1.0 eq.) in carbon tetrachloride (50 mL) was prepared, and benzoyl peroxide (323 mg, 1.00 mmol, 0.10 eq.) was added. The mixture was heated at 75 °C for 16 h, then cooled to room temperature, filtered, and concentrated under reduced pressure. The crude product was purified by flash column chromatography (pentane/ethyl acetate, 100:0 → 9:1) to afford compound **29b** (608 mg, 2.50 mmol, 25 %) as a colorless oil.

TLC (pentane:EtOAc = 95:5) R_f = 0.37 ($KMnO_4$)

^{1}H NMR (400 MHz, $CDCl_3$) δ [ppm] = 7.24 (t, J = 7.9 Hz, 1H), 7.08 (d, J = 7.7 Hz, 1H), 6.96 (d, J = 8.1 Hz, 1H), 4.47 (s, 2H), 2.43 (s, 3H), 2.38 (s, 3H).

^{13}C NMR (101 MHz, $CDCl_3$) δ [ppm] = 169.28, 149.43, 139.18, 129.39, 128.33, 128.12, 120.70, 24.77, 21.13, 18.99.

HRMS (ESI-LTQ-FT, positive) m/z: $[M+H]^+$ calcd. for $C_{10}H_{12}O_2{}^{79}Br^+$ 243.00152, calcd. for $C_{10}H_{12}O_2{}^{81}Br^+$ 244.99947; found 243.00171, 244.99975.

2.2.24 General Procedure D for the Zincation of Benzyl Bromides

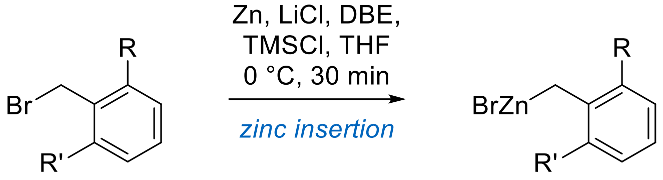

29a
29b

30a
30b

Lithium chloride (1.5 eq.) was placed in a Schlenk flask equipped with a magnetic stir bar and septum and heated with a heat gun (400 °C) under high vacuum for 10 min. The flask was flushed with argon (3×) and cooled to room temperature. Zinc dust (1.5 eq.) and THF were added. 1,2-Dibromoethane (0.05 eq.) was added, and the mixture was heated until ebullition occurred. After cooling to room temperature, trimethylsilyl chloride (0.01 eq.) was added, and the mixture was heated again until ebullition occurred. The mixture was cooled to 0 °C before a solution of benzyl bromide (1.368 g, 8.00 mmol, 1.0 eq.) in THF was added at 0 °C. The reaction mixture was stirred for 30 min before the remaining zinc dust was allowed to settle, and the concentration of the resulting benzylic zinc bromide solution was determined by iodometric titration.

2.2.24.1 Benzylzinc bromide (30a)

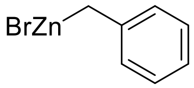

30a (99%)

Benzyl bromide (**29a**) was used and the experiment was performed on an 8.0 mmol scale in a total volume of 4 mL THF according to General Procedure D (2.2.24). The concentration of the active organozinc species was determined to be 1.96 M by iodometric titration, corresponding to a yield of 7.92 mmol (99 %).

2.2.24.2 (2-acetoxy-6-methylbenzyl)zinc bromide (30b)

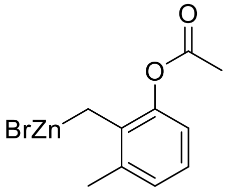

30b (17%)

2-(Bromomethyl)-3-methylphenyl acetate (**29b**) was used and the experiment was performed on a 0.50 mmol scale in a total volume of 0.5 mL THF according to General

Procedure D (2.2.24). The concentration of the active organozinc species was determined to be 0.17 M by iodometric titration, corresponding to a yield of 84 µmol (17 %).

2.2.25 2-Benzylcyclohex-2-en-1-one (31a)

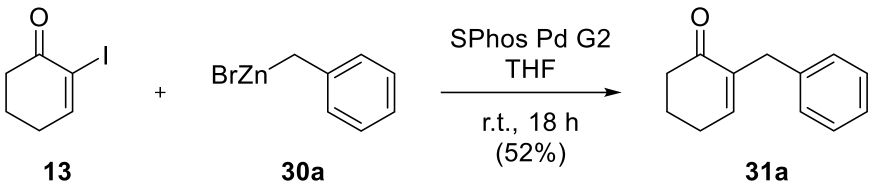

2-Iodocyclohex-2-en-1-one (**13**, 185 mg, 0.833 mmol, 1.0 eq.) and SPhos Pd G2 (60 mg, 0.0833 mmol, 0.10 eq.) were dissolved in THF (1 mL). After stirring for 5 min at room temperature, benzylic zinc bromide (**30a**, 1.96 M solution in THF, 0.51 mL, 1.00 mmol, 1.2 eq.) was added. The reaction mixture was stirred overnight (20 h) at room temperature. The reaction was quenched with saturated aqueous NH_4Cl and extracted with diethyl ether (3 × 25 mL). The combined organic layers were washed with aqueous sodium thiosulfate, dried over anhydrous magnesium sulfate, filtered, and concentrated under reduced pressure. Purification of the crude residue by flash column chromatography (pentane/ethyl acetate, 98:2 → 95:5 → 9:1) afforded the product (**31a**, 80.9 mg, 0.434 mmol, 52.1 %) as a colorless oil.

TLC (pentane:EtOAc= 8:2) R_f = 0.77 ($KMnO_4$)

^{1}H NMR (400 MHz, $CDCl_3$) δ [ppm] = 7.31–7.24 (m, 2H), 7.22–7.13 (m, 3H), 6.55 (tt, J = 4.3, 1.4 Hz, 1H), 3.54–3.50 (m, 2H), 2.48–2.41 (m, 2H), 2.37–2.29 (m, 2H), 2.02–1.94 (m, 2H).

2.2.26 3-Methyl-2-(tributylstannyl)cyclohex-2-en-1-one (33)

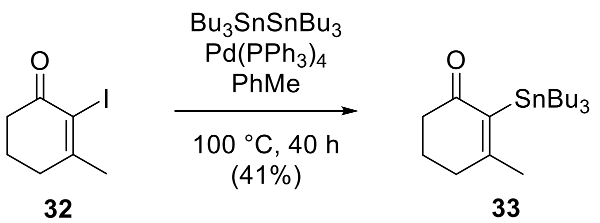

A flame-dried round-bottom flask equipped with a reflux condenser was charged with 2-iodo-3-methylcyclohex-2-en-1-one (**32**, 201 mg, 850 µmol, 1.0 eq.), bis(tributyltin) (1.33 mL, 2.55 mmol, 3.0 eq.), and tetrakis(triphenylphosphine)palladium(0) (99.2 mg, 85.0 µmol, 0.10 eq.) in toluene (25 mL). The mixture was degassed by bubbling argon through the solution for 15 min and then heated at reflux for 40 h. The reaction mixture was cooled to

room temperature and concentrated under reduced pressure The residue was purified by flash column chromatography (pentane/DCM, 100:0 → 8:2 → 1:1) to afford stannane **33** (138 mg, 346 µmol, 41 %).

TLC (pentane:DCM= 1:1) R_f = 0.70 ($KMnO_4$)

^{1}H NMR (500 MHz, $CDCl_3$) δ [ppm] = 2.37–2.29 (m, 4H), 1.99 (s, 3H), 1.96–1.90 (m, 2H), 1.51–1.42 (m, 6H), 1.33–1.27 (m, 6H), 0.99–0.92 (m, 7H), 0.88 (t, J = 7.3 Hz, 9H).

2.2.27 2-Benzyl-3-methylcyclohex-2-en-1-one (34a)

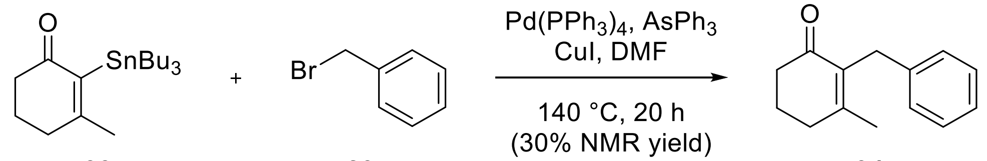

33 **29a** **34a**

A solution of stannane **33** (10 mg, 25 µmol, 1.0 eq.) and benzyl bromide (**29a**, 3.0 µL, 25 µmol, 1.0 eq.) in dry, degassed DMF (0.25 mL) was prepared. Tetrakis(triphenylphosphine)palladium(0) (1.5 mg, 1.3 µmol, 0.05 eq.), triphenylarsine (1.2 µL, 5.0 µmol, 0.20 eq.), and copper(I) iodide (4.8 mg, 25 µmol, 1.0 eq.) were added. The mixture was heated at 140 °C in the dark for 20 h. After cooling, DCM (5 mL) was added, and the mixture was extracted with 5 % aqueous LiCl (3×). The organic phase was dried by passing through a magnesium sulfate plug and concentrated under reduced pressure. 1,2-Dichloroethane was added as an internal standard, and the yield was determined by NMR (7.58 µmol, 30 %).

^{1}H NMR (400 MHz, $CDCl_3$) δ [ppm] = 7.26–7.20 (m, 2H), 7.17–7.11 (m, 3H), 3.69 (s, 2H), 2.46–2.41 (m, 2H), 2.41–2.37 (m, 2H), 2.03–1.92 (m, 5H), 1.96 (s, 4H).

3 Biochemical Methods

3.1 Materials

3.1.1 Chemicals

Table S1: List of chemicals used for biochemical experiments, along with their acronyms and the supplier.

Chemical	Acronym	Supplier
L-Glutathione, reduced form	GSH	*Sigma Aldrich*
Dimethyl sulfoxide	DMSO	*Sigma Aldrich/NEB*
Disodium hydrogen phosphate	Na_2HPO_4	*Carl Roth*
Ethanol	EtOH	*Carl Roth*
Fetal bovine serum	FBS	*Sigma Aldrich*
Glycerol		*Carl Roth*
Hydrochloric acid	HCl	*Fisher*
Potassium chloride	KCl	*Carl Roth*
Potassium dihydrogen phosphate	KH_2PO_4	*VWR*
Sodium bicarbonate	$NaHCO_3$	*Fisher*
Sodium chloride	NaCl	*VWR*
Sodium hydroxide	NaOH	*Fisher*
Ultrapure water	ddH_2O	
Poly-L-lysine	PLL	*Sigma Aldrich*
Accutase®		*Sigma Aldrich*
L-glutamine	L-Glu	*Sigma Aldrich*
Trypan blue		*invitrogen*
3-(4,5-Dimethyl-2-thiazolyl)-2,5-diphenyl-2H-tetrazolium bromide	MTT	*Sigma Aldrich*
Isotopically labelled desthiobiotin tags	isoDTB-tags	Synthesized inhouse according to procedure of Zanon *et al.*[4]
tert-Butanol	*t*BuOH	*Thermo scientific*
Copper sulfate	$CuSO_4$	*Carl Roth*
Tris((1-benzyl-4-triazolyl)methyl)amine	TBTA	*TCI*

Tris(2-carboxyethyl)phosphine	TCEP	*BLDpharm*
Sodium dodecyl sulfate	SDS	*Carl Roth*
Urea		*Sigma Aldrich*
Dithiothreitol	DTT	*Sigma Aldrich*
Iodoacetamide alkyne	IAA	Synthesized inhouse according to procedure of Zanon *et al.*[5]
Iodoacetamide	IA	*Sigma Aldrich*
Acetonitrile	MeCN	*Fisher*
Trifluoroacetic acid	TFA	*Fisher*
Formic acid	FA	*Fisher*
Triethylammonium bicarbonate buffer	TEAB	*Sigma Aldrich*
ß-Mercaptoethanol	BME	*Sigma Aldrich*
N-Acetyl cysteine methyl ester	NACME	*Sigma Aldrich*
N-Acetyl cysteine	NAC	*Sigma Aldrich*
N-Cbz-serine	Z-Ser	*BLDpharm*
*N*α-Acetyl lysine	NAL	*BLDpharm*
Triton X-100		*Sigma Aldrich*
Ammonia (as aqueous solution)	NH_3	*Supelco*

3.1.2 Buffers and Media

Table S2: List of buffers used for biochemical experiments, along with their composition.

Buffer/Medium	Composition
PBS	10 mM Na_2HPO_4<br>1.8 mM KH_2PO_4<br>140 mM NaCl<br>2.7 mM KCl<br>in ddH_2O<br>pH = 7.4
LB-medium (Carl Roth)	0.5 % (w/v) yeast extract<br>1.0 % (w/v) peptone<br>0.5 % (w/v) NaCl<br>in ddH_2O<br>pH = 7.5

BHI-medium (Carl Roth)	0.75 % (w/v) brain infusion<br>1.0 % (w/v) heart infusion<br>1.0 % (w/v) peptone<br>0.5 % (w/v) NaCl<br>0.25 % (w/v) Na_2HPO_4<br>0.2 % (w/v) glucose<br>in ddH_2O<br>pH = 7.4
B-medium (Carl Roth)	0.5 % (w/v) yeast extract<br>1.0 % (w/v) peptone<br>0.5 % (w/v) NaCl<br>0.1 % (w/v) K_2HPO_4<br>in ddH_2O<br>pH = 7.5
High glucose Dulbecco's Modified Eagle's Medium (DMEM, *Sigma Aldrich*)	

3.1.3 Bacterial cell lines

Bacteria were stored at -80 °C as cryostocks in a 1:1 mixture of culture medium and glycerol. Aliquots were shock frozen in liquid N_2 and only thawed once.

Table S3: Bacterial cell lines used for this project.

Organism	Strain	Medium
Acinetobacter baumannii	DSM 30007/ATCC 19606	LB
Bacillus licheniformis	ATCC 14580	LB
Bacillus subtilis	168	LB
Klebsiella aerogenes	DSM 30053	BHI
Escherichia coli	K12	LB
Pseudomonas aeruginosa	PAO1	LB
Salmonella enterica subsp. enterica	LT2	LB
Staphylococcus aureus	NCTC 8325	B
Staphylococcus aureus	USA300	B
Streptococcus pyogenes	ATCC 700294	BHI

3.2 Experimental Procedures

3.2.1 Minimal Inhibitory Concentration (MIC) Assay

5 mL of medium in a culture tube were inoculated at a 1:100 ratio with an overnight culture of the desired bacteria. The culture tube was then incubated until it reached at least 0.5 McFarland units. 2 µL of a DMSO stock at 100 times the highest test concentration were added to wells B–D and E–G in the first column of a 96-well plate (two different conditions in technical triplicates per plate). A sterile-filtered aqueous 500 mM $NaHCO_3$ solution was diluted 1:20 in culture medium. 100 µL of this medium were added to each well in rows A and H to serve as a sterile control. Additionally, 50 µL of medium were dispensed into columns 2–12 of rows B–G. 98 µL of medium were added to the stocks in wells B–G of column 1, resulting in a final volume of 100 µL with twice the highest test concentration of the compound. The diluted compounds in the first column were thoroughly mixed by pipetting up and down. From the first column, 50 µL were transferred to the second column and mixed, followed by sequential transfers of 50 µL from one column to the next until column 10 was reached. 50 µL from column 10 were transferred to column 12, which served as the sterile control for the dilution process. Column 11 served as the untreated growth control. If necessary, the bacterial suspension was diluted to 0.5 McFarland units with fresh medium. The suspension was then diluted 1:100 in fresh medium and 50 µL of this suspension were then added to all wells except the sterile controls. The plate was incubated for 16 h at 37 °C with shaking at 200 rpm. Optical density (OD_{600}) values were measured using a plate reader (*Tecan* Infinite® 200 Pro M Nano). The lowest concentration where no bacterial growth was observed ($OD_{600} \leq 0.1$) represents the MIC.

3.2.2 Cytotoxicity Determination *via* Metabolic Activity Measurement (MTT Assay)

Cytotoxicity was assessed in human embryonic kidney 293 cells (HEK 293) in 96-well plates (Transparent Nunc 96-well flat bottom, cell culture treated, *Thermo Fisher Scientific*) coated with poly-L-lysine. To coat the plates, a 0.01 % aqueous solution of poly-L-lysine was used (50 µL per well). The solution was evenly distributed by tilting the plate, followed by a 10 min incubation at room temperature. The coating solution was then aspirated, the wells were rinsed with 200 µL PBS and then left to dry without lid for 15 min. The cells were

cultured in a T175 flask in high glucose Dulbecco's Modified Eagle's Medium (DMEM), supplemented with 10 % FBS and 2mM L-glutamine. After removing the medium, cells were washed with PBS and detached by incubating with 2mL Accutase® for 5 min at 37 °C, followed by tapping the flask. 8mL of fresh medium were added to wash the cells from the flask wall. The cell suspension was gently mixed and transferred to a 15 mL Falcon tube, centrifuged at 500 rpm for 5 min, the supernatant removed, and the pellet resuspended in 10mL fresh medium. The concentration of viable cells was determined using trypan blue stain, a Neubauer counting chamber and a stereomicroscope. HEK 293 cells were seeded at a density of 4000 cells per well in 100 µL medium on the poly-L-lysine-coated 96-well plates. To prevent edge effects, wells in rows A and H and columns 1 and 12 were filled with 200 µL medium or PBS. The cells were incubated overnight at 37 °C and 5 % CO_2. For compound treatment, a dilution series of the compound was prepared from a DMSO stock in DMEM without FBS, ensuring a final DMSO concentration of 1 %. Medium was carefully removed from the wells without disturbing the cell layer, and 100 µL of the compound dilutions or DMSO control were added to each well. Plates were incubated for 24 h at 37 °C and 5 % CO_2. After incubation, 20 µL of 3-(4,5-dimethyl-2-thiazolyl)-2,5-diphenyl-2H-tetrazolium bromide solution (MTT, 5 mg mL^{-1} in PBS) were added to each well using a stepper pipette. The plate was incubated in the dark for 1 h at 37 °C and 5 % CO_2. Following incubation, the medium was carefully removed, and formazan crystals were solubilized in 200 µL DMSO per well. Plates were placed on a rocker at 300 rpm for 10 min. Absorbance at 570 nm (formazan) and 630 nm (background) was measured using a plate reader (*Tecan* Infinite® 200 Pro M Nano). The assay was performed in n = 3 biologically independent replicates with three technical replicates each. IC_{50} values (concentration at which 50 % viability is reached) were determined using GraphPad Prism v10.0.1. The background was subtracted, the absorption values of technical replicates were averaged and the resulting values were normalized to the upper and lower asymptote of a nonlinear fit of the data ([Inhibitor] vs. response – variable slope (four parameters)) for each compound tested, followed by re-fitting a nonlinear regression to the normalized data ([Inhibitor] vs. normalized response – variable slope) according to a protocol by Krebs *et al.*[3]

3.2.3 Competitive Residue-Specific Chemoproteomics

Sample preparation: The experiment was adapted from published procedures.[4,8] Day cultures of four different colonies of *E. coli* K12 were diluted 1:100 into fresh LB medium and grown to early stationary phase (37 °C, 200 rpm). Each culture was split into two 50 mL centrifuge tubes, harvested (2 × 50 mL, 6,000 ×g, 4 °C, 10 min), washed with PBS (25 mL, 6,000 ×g, 4 °C, 10 min) and recombined. Cells were resuspended in 5 mL PBS and transferred to pre-packed bead-mill lysing tubes (Precellys Lysing Kit CK01, 7 mL, *Bertin Technologies*). Cells were disrupted by bead beating for three cycles of 30 s at 6500 rpm with 30 s cooling intervals inbetween using a bead mill (Precellys 24 Homogenizer, *Bertin Technologies*) with liquid nitrogen cooling. Lysates were aliquoted to microcentrifuge tubes and cleared by centrifugation (21,000 ×g, 4 °C, 45 min). The protein concentration was determined (ROTI Quant universal, *Roth*) and adjusted to 2 mg/mL.

100 µL aliquots of the adjusted samples were treated with 2 µL 10 mM *rac*-11-deoxy-MikA or *rac*-leptosphaerone B (in DMSO) or 2 µL DMSO and incubated for 1 h (r.t., 200 rpm). Subsequently, the samples were treated with 2 µL iodoacetamide-alkyne (50 mM stock in DMSO for a final concentration of 100 µM) and incubated for 1 h (20 °C, 1400 rpm). The samples were clicked to the heavy (DMSO-treated) and light (compound-treated) isoDTB-tags by adding 14 µL of the following mixture: 6 µL TBTA ligand (0.9 mg/mL in 4:1 *t*BuOH/DMSO), 2 µL copper sulfate (12.5 mg/mL in ddH2O), 2 µL TCEP (13 mg/mL in ddH2O), 2 µL of the respective isoDTB tag (50 mM stock in DMSO) and 2 µL SDS (10 % in PBS). After incubation at room temperature for 1 h, 1 µL DNAse I (*Roche*) was added and the samples were incubated for 30 min at 37 °C.

A 1:1 mix of hydrophobic and hydrophilic carboxylate-coated magnetic beads (*Cytiva*, cat. 65152105050250 and 45152105050250, 50 µg/µL) was washed three times with H_2O and subsequently aliquoted (80 µL) to LoBind tubes (*Eppendorf*). The corresponding heavy- and light-tagged samples were combined onto the 80 µL bead mixture and incubated for 5 min while shaking (1000 rpm), followed by the addition of 800 µL absolute ethanol and 5 min incubation while shaking (1000 rpm). The tubes were placed on a magnetic rack, the supernatant was removed, and the beads were washed three times with 400 µL of 80 % ethanol. The beads were resuspended in 200 µL of 2 M urea in 0.5 % SDS in PBS. A 31 mg/mL solution of DTT in water (10 µL) was added, and the mixture was incubated for

15 min at 65 °C. Subsequently, 10 µL of a 71 mg/mL solution of iodoacetamide in water was added, followed by incubation for 30 min at 37 °C with shaking at 1000 rpm. Absolute ethanol (400 µL) was added, and the suspension was incubated for an additional 5 min at room temperature while shaking at 1000 rpm. The tubes were placed on a magnetic rack, the supernatant was removed, and the beads were washed three times with 400 µL of 80 % ethanol. The beads were resuspended in 200 µL of 2 M urea in PBS, and trypsin (8 µL of a 0.5 mg/mL solution; 2 µg total, *Promega*, V511C) was added. Digestion was carried out overnight at 37 °C in a shaking incubator (200 rpm).

The tubes were placed on a magnetic rack and the supernatant containing the digested peptides was collected and transferred to fresh 2 mL LoBind tubes. Peptides remaining on the beads were eluted by adding 50 µL of 2 % DMSO in water, followed by incubation for 30 min at 37 °C with shaking at 1000 rpm. After magnetic separation, the eluate was transferred to the previously collected supernatant. This elution step was repeated once, resulting in a combined eluate volume of 300 µL.

50 µL of Streptavidin high-capacity beads (*Thermo Fisher scientific*, cat. 20359) per sample were washed three times with 10 mL PBS (centrifugation at 1000 ×g for 3 min) and finally resuspended in 1200 µL PBS per sample. The bead suspension (1200 µL per sample) was added to the peptide solutions and the mixture was incubated for 1 h at room temperature while rotating. After centrifugation at 1000 ×g for 3 min, the supernatant was removed. The beads were resuspended in 600 µL PBS and transferred to Pierce™ centrifuge columns (*Thermo Fisher scientific*, cat. 89868). The liquid was forced through the columns by gentle pressure. The beads were washed twice with 600 µL PBS, followed by three washes with 600 µL water, and three washes with 600 µL of 50 % acetonitrile in water. Peptides were eluted into fresh LoBind tubes with 1 × 200 µL and 2 × 70 µL 0.1 % TFA in 50% acetonitrile, followed by a final centrifugation step (3,000 ×g, 3 min). The samples were dried using a centrifugal vacuum concentrator (*Eppendorf*) at 45 °C. The dried samples were stored at −20 °C.

Peptides were reconstituted in 30 µL 0.1% TFA. The samples were sonicated for 5 min, vortexed, and centrifuged. This process was repeated twice more. Ultrafree-MC centrifugal filters (0.22 µM, UFC30GVNB, *Millipore*) were pre-washed with 300 µL of 0.1 % TFA before the samples were filtered through by centrifugation at 17000 ×g for 2 min. Filtrates were transferred to MS vials for LC-MS/MS analysis.

LC-MS/MS Measurements on Orbitrap Eclipse: The samples were analyzed by HPLC-MS/MS at an injection volume of 1.25 µL in non-systematic order, with a blank run after every four samples on a Vanquish Neo UHPLC (*Thermo Fisher scientific*) coupled to an Orbitrap Eclipse Tribrid mass spectrometer (*Thermo Fisher scientific*). The first sample was re-measured at the end of the sequence for performance comparison. The Vanquish Neo UHPLC was equipped with a PepMap Neo 5 µm C18 300 µm × 5 mm trap cartridge (*Thermo Fisher scientific*) and an Aurora Ultimate separation column (3rd generation, 1.7 µm C18, 75 µm × 25 cm nanoflow UHPLC compatible, *IonOpticks*) with a Nanospray Flex Ion Source (*Thermo Fisher scientific*), and was operated in the Trap-and-Elute-Injection mode. Samples were loaded onto the trap column, and the subsequent separation was carried out with a flow rate of 400 nL/min using mobile phase A (0.1 % FA in water) and mobile phase B (0.1 % FA in MeCN). The separation column was heated to 40 °C. The HPLC method comprised 75 min and started with a gradient from 5 % to 40 % B over a period of 60 min, followed by a second gradient up to 60 % B within 5 min and an isocratic period of 10 min at 90 % B. Separation column washing and equilibration were conducted with enabled fast equilibration, equilibration factor “3” at 5 % B. Trap column washing and equilibration are conducted with enabled fast wash and equilibration together with zebra wash (2 wash cycles, automatic equilibration factor).

The *Orbitrap Eclipse Tribrid* mass spectrometer was run with an internal real-time mass calibration using a user-defined lock mass (positive, m/z = 445.12003) and operated in data-dependent acquisition mode. The full MS scans were collected in the orbitrap at a resolution of 120,000 and an AGC target of 4e5 with automated maximum injection time in a scan range of 300–1500 m/z. For MS^2 scans, the TOP10 intense ions with a charge state of 2–7 were selected with a minimum intensity threshold of 5e3 and enabled isotope exclusion and dynamic exclusion for 30 s whereby peaks with charge state 1 or unassigned charge state were excluded. MS^2 spectra were collected at a resolution of 15,000 and an AGC target of 5e4 with automated maximum injection time. Isolation in the quadrupole was conducted using a window of 1.6 m/z. Higher-energy collision-induced dissociation with normalized collision energy of 30 % was used to generate the fragments which were detected in the orbitrap. Data acquisition was performed using Thermo Scientific *Foundation Software 3.1sp9* and *Xcalibur 4.6*.

Data Analysis of Orbitrap Eclipse Measurements: Data evaluation was performed based on protocols published by Hacker *et al.*[5]

Acquired raw files were converted into mzML format using the tool *MSConvert 3.0.25054-207c92d* of the software *ProteoWizard 3.0*.[9] Standard settings were used with vendor´s peak picking enabled. Further analysis was performed with the *FragPipe v22.0* interface with *MSFragger 4.1*,[10,11] *IonQuant 1.10.27*,[12] *diaTracer 1.1.5*,[13] *DIA-NN 1.8.2 beta 8*,[14] and *Python 3.11.11*. Every experiment (Supplementary Figure S2**:** C and D) was analyzed in a different run. The global settings were set to "0"(=auto) RAM and "4" for parallelism. A "closed search" was conducted based on a FASTA file of the UniProt reference proteome for *E. coli* K12 (proteome ID: UP000000625, taxon ID: 83333, downloaded on 22.04.2025)[15,16] and reverse sequences were added automatically. The datasets were analyzed with the following settings: Precursor mass tolerance was set from −20 to 20 ppm, with a fragment mass tolerance of 20 ppm. The calibration and optimization were set to "Mass calibration, parameter optimization" and the isotope error to "0/1/2". For protein digestion, an "enzymatic" cleavage was entered with enabled clip N-term and "trypsin" as enzyme name and the trypsin cleavage rules (cut after "KR", but no cleavage after "P" and "2" missing cleavage, sense "C"). Peptide length was given with 6 to 50 in a peptide mass range of 500−5,000 Da. Variable modifications were given with a maximum of "3" per peptide and a maximum of "5,000" combinations and all modifications were enabled in the first search. The following settings were used as mass delta at the respective site: 15.9949 (M), 42.0106 ([^), 561.3387 (C), 567.3462 (C). The fixed modification is enabled for all amino acids with 0.0, except for cysteine (57.02146). Mass offsets and labile modifications were disabled and all other settings for *MSFragger* were kept as default. Run validation tools were activated, and Cristal-C was disabled. The following settings for Rescoring using deep learning prediction were chosen: MSBooster, prediction of RT and spectra prediction were enabled whereas correlated features were not activated. PSM validation was set to percolator with a min probability of "0.5" and "-only psms --no terminate --post-processing-tdc" as command line options. ProteinProphet was enabled with "--maxppmdiff 2000000" and FDR filter and reports are activated to generate reports using "--sequential --prot 0.01" as filter. Other settings for the FDR filter, PTMShepherd and O-Pair were disabled. For MS^1 quantification, Run MS^1 quant, IonQuant and MaxLFQ were enabled with MaxLFQ min ions set to "2". The masses for labelling were given with C561.3387 for light and C567.3462 for heavy. Re-

quantification was activated, match between runs (MBR) and intensity normalization across the runs were not enabled. Feature detection and peak tracing were conducted with min. scans of "3" and a min. isotopes of "2", using a tolerance of 10 ppm for m/z, 0.4 min for RT and 0.05 $1/k_0$ for IM. The other settings for MS^1 quantification were kept as default. Run TMT-Integrator for isobaric labelling-based Quantification and spectral library generation was disabled. For downstream data analysis, the "ion_label_quant.tsv" files of the individual replicates were analyzed separately. The "Modified peptide" was generated as either the "Light Modified Peptide" or the "Heavy Modified Peptide" based on the entry with the higher Intensity, for each entry. The masses of probe modification in the "Modified Peptide" were replaced by an "*" and the mass of carbamidomethylation ([57.0215]) in this entry was deleted, if present. The full protein sequence was linked into the table. Based on this information, all peptide sequences that do not occur exactly once in the protein were excluded and the residue number of the modified residue was determined. The "identifier" was generated in the format "UniProtCode" C_"residue number", where C is the one letter code of the modified amino acid cysteine. For each "identifier", the median "Log_2 ratio HL", which is the log_2 transformed ratio of the heavy and light channels, was determined as median of the "Log_2 ratio HL" column of all corresponding ions and renamed "Log_2R_replicate name". If several different "Modified peptides" were detected for the same "Identifier", the "Modified Peptide" with the shortest sequence was kept. For all identifiers, the data for all replicates was now combined into one table. If different "Modified peptides" were detected for the same "Identifier" in the different replicates, the "Modified Peptide" with the shortest sequence was kept. The average of the Log_2R values of the individual replicates was calculated and named "Log_2R_Average" and only kept, if it was quantified in at least 2 out of 4 replicates.

Downstream analysis was performed with the Perseus[17] software (version 2.0.11.0) according to a published procedure.[4] The data for each replicate of the same condition were loaded into Perseus before the rows were filtered based on at least two valid values. A two-sided one-sample t-test was conducted against a value of $log_2(R) = 0$. The median values of $log_2(R)$ and $-log_{10}(p)$ derived from Perseus were used for the visualization in a volcano plot. The requirements for the identification as "hit" were a statistical significance $p < 0.05$ and a median ratio $log_2(R) > 1$. The UniProt identifier is linked to the data based on peptide sequence.

3.2.4 *In vitro* Reactivity Profiling

To a tapered MS-vial were added 0.4 µL pf an electrophile stock (*rac*-11-deoxy-MikA or *rac*-leptosphaerone B, 20 mM in DMSO) and 1.0 µL of a nucleophile stock (β-mercaptoethanol, *N*-acetyl cysteine methyl ester, *N*-acetyl cysteine, and *N*-Cbz-serine: 80 mM in MeCN; *N*α-acetyl lysine and glutathione: 80 mM in 1:1 H_2O:MeCN), as well as 18.6 µL water adjusted to pH 9.0 using an aqueous solution of ammonia, resulting in a final electrophile concentration of 400 µM and a final nucleophile concentration of 4 mM (1:10 ratio). The samples were vortexed for 5 s and incubated for 60 min at 37 °C in a water bath. During the first and last 3 min of incubation, the samples were sonicated. 10 µL of the reaction mixtures were subsequently analyzed by HPLC-MS on a QExactive™ Plus mass spectrometer, coupled to a DIONEX Ultimate 3000 HPLC system (both *Thermo Fisher scientific*). Electrospray ionization (ESI) was used as the ionization method. MS data were evaluated with THERMO Xcalibur 2.1 (*Thermo Fisher scientific*).

3.2.5 Full Proteome Analysis

Sample preparation: The procedure was adapted from Schum *et al.*[18] An overnight culture of *S. aureus* NCTC 8325 was used to inoculate one day culture per sample (quadruplicates of every treatment condition) to an initial OD_{600} of 0.05 in 5 mL B medium. The cultures were cultivated for 2 h at 37 °C and 200 rpm before optical densities were recorded (BioPhotometer model 6131, *Eppendorf*). Upon reaching $OD_{600} \approx 0.5$, 990 µL of each culture were transferred to an Eppendorf tube and treated with either DMSO (1 %) or one of the following compounds: *rac*-leptosphaerone B (200 µM), *rac*-11-deoxy-MikA (200 µM) or *rac*-dihydro-MikA (100 µM). Cells were cultivated for a further 1 h at 37 °C and 200 rpm. The suspensions were pelleted (6000 ×g, 10 min, 4 °C) and washed once with 1 mL of ice-cold PBS and pelleted again (6000 ×g, 10 min, 4 °C). Pellets were resuspended in 150 µL PBS containing 0.5 % SDS and 1 % Triton X-100. Cells were disrupted by a 10 s sonication pulse at 30 % intensity (Sonopuls HD 2070, *Bandelin*) followed by bead beating in bead-mill tubes (0.1 mm zirconia beads) for three cycles of 30 s at 6500 rpm with 30 s cooling intervals inbetween (Precellys 24 Homogenizer, *Bertin Technologies*). Lysates were centrifuged at 10,000 ×g for 10 min, transferred to new microcentrifuge tubes, and centrifuged at 21,000 ×g for 30 min (r.t.). The final supernatants were transferred to new LoBind tubes (*Eppendorf*), and the protein concentrations were quantified by BCA assay (Roti Quant, *Carl*

Roth). Equal amounts of protein (8.55 µg) were transferred to a V-bottom 96-well polypropylene plate (*Greiner*, cat. 651201) and the volume was adjusted to 100 µL using PBS containing 0.5 % SDS and 1 % Triton X-100. For reduction/alkylation, 3 µL of a 1:2 mixture of TCEP and iodoacetamide (each 500 mM) were added, incubated for 15 min at 950 rpm (r.t.), and excess iodoacetamide was quenched with 2 µL 500 mM DTT. A 1:1 mix of hydrophobic and hydrophilic carboxylate-coated magnetic beads (*Cytiva*, cat. 65152105050250 and 45152105050250, 50 µg/µL) was washed three times with H_2O and subsequently added to the plate (2 µL per well), followed by 150 µL EtOH to precipitate proteins. The samples were thoroughly mixed by pipetting up and down. All further liquid-handling steps were executed on a *Hamilton* Microlab Prep robot. After 5 min of shaking (500 rpm, r.t.), beads were captured on a 96-ring magnet (Magnum FLX, *Alpaqua*), and supernatants were withdrawn slowly at 20 µL s^{-1} to prevent bead loss. Beads were washed three times with 180 µL 80 % EtOH and once with 180 µL MeCN, with off-magnet shaking between wash steps (1 min, 800 rpm, r.t.). Proteins were digested overnight at 37 °C in 100 µL 50 mM TEAB containing 0.2 µL sequencing-grade trypsin (trypsin:protein 1:100, 0.5 µg/µL; *Promega*) while shaking at 800 rpm under a heated lid with a tightly sealed plate. Peptides were then eluted with 50 µL 3 % formic acid, desalted on two-disk styrenedivinylbenzene-reverse phase sulfonate (SDB-RPS) StageTips (Empore, *3M*), which were equilibrated with 150 µL wash buffer 1 (1 % TFA in isopropanol).[19] Samples were loaded (10 min, 500 ×g), washed with buffer 1 (30 min, 800 ×g) and buffer 2 (0.2 % TFA in H_2O; 30 min, 800 ×g). Peptides were eluted using 50 µL elution buffer (1 % NH_3, 80 % MeCN) by centrifugation (5 min at 300 ×g and then 800 ×g) and dried in a centrifugal evaporator (Concentrator Plus, *Eppendorf*). Dried peptides were reconstituted in 128.3 µL 1 % formic acid (0.0667 µg/µL protein) and 4 µL were injected on a *Bruker* timsTOF Pro operated in DIA mode. All conditions were examined in four replicates, except the DMSO control, which was evaluated in triplicate, as one sample had to be discarded.

LC-MS/MS Measurements on timsTOF Pro: The procedure was adapted from Schum *et al.*[18] Peptide separation and mass spectrometry were performed using an UltiMate 3000 nano HPLC system (*Thermo Fisher Scientific*) coupled to a timsTOF Pro instrument (*Bruker*) via a CaptiveSpray nano-electrospray ion source and a Sonation column oven. Samples were first loaded onto a trap column (Acclaim PepMap 100 C18, 75 µm × 2 cm, 3 µm particle size, *Thermo Fisher Scientific*) and washed for 7 min using solvent A (0.1 %

formic acid in water) at a flow rate of 5 µL min^{-1}. The peptides were then eluted onto a separation column (Aurora C18, 25 cm × 75 µm, 1.7 µm particle size, *IonOpticks*) and separated with a gradient of solvent B (0.1 % formic acid in MeCN) at a constant flow of 400 nL min^{-1}. The elution profile consisted of 5–28 % B over 28 min, 28–40 % B over the next 6 min, and finally a high organic wash at 95 % B for 6 min before re-equilibration (5 % B for 10 min). The mass spectrometer operated in dia-PASEF mode. Ion mobility separation was conducted using a dual TIMS analyzer with equal accumulation and ramp times of 100 ms, covering a $1/K_0$ ion mobility range from 0.60 V s cm^{-2} to 1.60 V s cm^{-2} for MS^1 scans. Fragmentation was carried out over an m/z range of 400–1201 using an ion mobility window of 0.60 V s cm^{-2} to 1.43 V s cm^{-2}. Each dia-PASEF scan included two ion mobility isolation windows with 26 m/z width. The full mass range was covered using 32 windows with 1 m/z overlaps, yielding 16 dia-PASEF scans per MS^1 cycle and an overall cycle time of approximately 1.80 s (see Table S4). Collision energy was decreased linearly from 59 eV at $1/K_0$ = 1.3 V s cm^{-2} down to 20 eV at $1/K_0$ = 0.85 V s cm^{-2}. TIMS elution voltage calibration was performed using three reference ions (m/z 622, 922, and 1222) from the *Agilent* ESI-L Tuning Mix, which were spiked directly into the CaptiveSpray inlet filter to obtain accurate reduced ion mobility coefficients ($1/K_0$).

Table S4: DIA-PASEF scan windows including ion mobility range ($1/K_0$) and scan width (m/z).

MS Type	Scan	Scan Start [$1/K_0$]	Scan End [$1/K_0$]	Mass Start [m/z]	Mass End [m/z]
MS1	0	0.00	0.60	100	1700
dia-PASEF	1	0.90	1.20	800	826
dia-PASEF	1	0.60	0.90	400	426
dia-PASEF	2	0.92	1.22	825	851
dia-PASEF	2	0.62	0.92	425	451
dia-PASEF	3	0.93	1.23	850	876
dia-PASEF	3	0.63	0.93	450	476
dia-PASEF	4	0.95	1.25	875	901
dia-PASEF	4	0.65	0.95	475	501
dia-PASEF	5	0.96	1.26	900	926
dia-PASEF	5	0.66	0.96	500	526
dia-PASEF	6	0.98	1.28	925	951
dia-PASEF	6	0.68	0.98	525	551
dia-PASEF	7	0.99	1.29	950	976
dia-PASEF	7	0.69	0.99	550	576
dia-PASEF	8	1.01	1.31	975	1001

dia-PASEF	8	0.71	1.01	575	601
dia-PASEF	9	1.02	1.32	1000	1026
dia-PASEF	9	0.72	1.02	600	626
dia-PASEF	10	1.04	1.34	1025	1051
dia-PASEF	10	0.74	1.04	625	651
dia-PASEF	11	1.06	1.36	1050	1076
dia-PASEF	11	0.76	1.06	650	676
dia-PASEF	12	1.07	1.37	1075	1101
dia-PASEF	12	0.77	1.07	675	701
dia-PASEF	13	1.09	1.39	1100	1126
dia-PASEF	13	0.79	1.09	700	726
dia-PASEF	14	1.10	1.40	1125	1151
dia-PASEF	14	0.80	1.10	725	751
dia-PASEF	15	1.12	1.42	1150	1176
dia-PASEF	15	0.82	1.12	750	776
dia-PASEF	16	1.13	1.43	1175	1201
dia-PASEF	16	0.83	1.13	775	801

Data Analysis of timsTOF Pro Measurements: The MS data was first processed using DIA-NN[14] in library-free mode. For library generation, the UniProt reference proteome for *S. aureus* NCTC 8325 (proteome ID: UP000008816, taxon ID: 93061, downloaded on 2025/11/25) was used.[16,20] The DIA-NN configuration file was uploaded on the repositories referenced in the data availability section. The resulting LFQ intensities were then processed using Perseus software[17] (version 2.0.11.0). LFQ intensities were log_2-transformed, and samples were grouped according to treatment groups. Then, protein groups were filtered to retain those with at least 3 valid values in one group. For statistical analysis, two-sample Student's t-tests with permutation-based multiple testing correction (FDR = 0.05) were used, always comparing the antibiotic-treated group with the corresponding DMSO control group.

4 References

(1) Johnson, C. R.; Barbachyn, M. R. Sulfoximine-Directed Osmylation: Synthesis of Enantiomerically Pure Dihydroxycycloalkanones. *J. Am. Chem. Soc.* **1984**, *106* (8), 2459–2461. https://doi.org/10.1021/ja00320a053.

(2) Haiza, M.; Lee, J.; Snyder, J. K. Asymmetric Syntheses of Salvia Miltiorrhiza Abietanoid O-Quinones: Methyl Tanshinonate, Tanshinone IIB, Tanshindiol B and 3-Hydroxytanshinone. *J. Org. Chem.* **1990**, *55* (17), 5008–5013. https://doi.org/10.1021/jo00304a009.

(3) Krebs, A.; Nyffeler, J.; Rahnenführer, J.; Leist, M. Normalization of Data for Viability and Relative Cell Function Curves. *ALTEX - Altern. Anim. Exp.* **2018**, *35* (2), 268–271. https://doi.org/10.14573/1803231.

(4) Zanon, P. R. A.; Lewald, L.; Hacker, S. M. Isotopically Labeled Desthiobiotin Azide (isoDTB) Tags Enable Global Profiling of the Bacterial Cysteinome. *Angew. Chem. Int. Ed.* **2020**, *59* (7), 2829–2836. https://doi.org/10.1002/anie.201912075.

(5) Zanon, P. R. A.; Yu, F.; Musacchio, P. Z.; Lewald, L.; Zollo, M.; Krauskopf, K.; Mrdović, D.; Raunft, P.; Maher, T. E.; Cigler, M.; Chang, C. J.; Lang, K.; Toste, F. D.; Nesvizhskii, A. I.; Hacker, S. M. Profiling the Proteome-Wide Selectivity of Diverse Electrophiles. *Nat. Chem.* **2025**, *17* (11), 1712–1721. https://doi.org/10.1038/s41557-025-01902-z.

(6) Shiner, C. S.; Berks, A. H. Preparation of (+)- and (-)-N,S-Dimethyl-S-Phenylsulfoximine via an Improved Resolution. Accurate Determination of Very High Enantiomeric Purities by on-Column GC Analysis of Diastereomeric Derivatives. *J. Org. Chem.* **1988**, *53* (23), 5542–5545. https://doi.org/10.1021/jo00258a029.

(7) Matsuo, J.; Iida, D.; Tatani, K.; Mukaiyama, T. A New Method for Oxidation of Various Alcohols to the Corresponding Carbonyl Compounds by Using *N* - *t* - Butylbenzenesulfinimidoyl Chloride. *Bull. Chem. Soc. Jpn.* **2002**, *75* (2), 223–234. https://doi.org/10.1246/bcsj.75.223.

(8) Backus, K. M.; Correia, B. E.; Lum, K. M.; Forli, S.; Horning, B. D.; González-Páez, G. E.; Chatterjee, S.; Lanning, B. R.; Teijaro, J. R.; Olson, A. J.; Wolan, D. W.; Cravatt, B. F. Proteome-Wide Covalent Ligand Discovery in Native Biological Systems. *Nature* **2016**, *534* (7608), 570–574. https://doi.org/10.1038/nature18002.

(9) Kessner, D.; Chambers, M.; Burke, R.; Agus, D.; Mallick, P. ProteoWizard: Open Source Software for Rapid Proteomics Tools Development. *Bioinformatics* **2008**, *24* (21), 2534–2536. https://doi.org/10.1093/bioinformatics/btn323.

(10) Kong, A. T.; Leprevost, F. V.; Avtonomov, D. M.; Mellacheruvu, D.; Nesvizhskii, A. I. MSFragger: Ultrafast and Comprehensive Peptide Identification in Mass Spectrometry–Based Proteomics. *Nat. Methods* **2017**, *14* (5), 513–520. https://doi.org/10.1038/nmeth.4256.

(11) Yu, F.; Teo, G. C.; Kong, A. T.; Haynes, S. E.; Avtonomov, D. M.; Geiszler, D. J.; Nesvizhskii, A. I. Identification of Modified Peptides Using Localization-Aware Open Search. *Nat. Commun.* **2020**, *11* (1), 4065. https://doi.org/10.1038/s41467-020-17921-y.

(12) Yu, F.; Haynes, S. E.; Teo, G. C.; Avtonomov, D. M.; Polasky, D. A.; Nesvizhskii, A. I. Fast Quantitative Analysis of timsTOF PASEF Data with MSFragger and IonQuant. *Mol. Cell. Proteomics* **2020**, *19* (9), 1575–1585. https://doi.org/10.1074/mcp.TIR120.002048.

(13) Li, K.; Teo, G. C.; Yang, K. L.; Yu, F.; Nesvizhskii, A. I. diaTracer Enables Spectrum-Centric Analysis of diaPASEF Proteomics Data. *Nat. Commun.* **2025**, *16* (1), 95. https://doi.org/10.1038/s41467-024-55448-8.
(14) Demichev, V.; Messner, C. B.; Vernardis, S. I.; Lilley, K. S.; Ralser, M. DIA-NN: Neural Networks and Interference Correction Enable Deep Proteome Coverage in High Throughput. *Nat. Methods* **2020**, *17* (1), 41–44. https://doi.org/10.1038/s41592-019-0638-x.
(15) Blattner, F. R.; Plunkett, G.; Bloch, C. A.; Perna, N. T.; Burland, V.; Riley, M.; Collado-Vides, J.; Glasner, J. D.; Rode, C. K.; Mayhew, G. F.; Gregor, J.; Davis, N. W.; Kirkpatrick, H. A.; Goeden, M. A.; Rose, D. J.; Mau, B.; Shao, Y. The Complete Genome Sequence of Escherichia Coli K-12. *Science* **1997**, *277* (5331), 1453–1462. https://doi.org/10.1126/science.277.5331.1453.
(16) The UniProt Consortium. UniProt: The Universal Protein Knowledgebase in 2025. *Nucleic Acids Res.* **2025**, *53* (D1), D609–D617. https://doi.org/10.1093/nar/gkae1010.
(17) Tyanova, S.; Temu, T.; Sinitcyn, P.; Carlson, A.; Hein, M. Y.; Geiger, T.; Mann, M.; Cox, J. The Perseus Computational Platform for Comprehensive Analysis of (Prote)Omics Data. *Nat. Methods* **2016**, *13* (9), 731–740. https://doi.org/10.1038/nmeth.3901.
(18) Schum, D.; Elsen, F. A. V.; Ruddell, S.; Schorpp, K.; Junca, H.; Müsken, M.; Chen, S.-Y.; Fiedler, M. K.; Pickl, T.; Pieper, D. H.; Hadian, K.; Zacharias, M.; Sieber, S. A. Screening Privileged Alkyl Guanidinium Motifs under Host-Mimicking Conditions Reveals a Novel Antibiotic with an Unconventional Mode of Action. *JACS Au* **2024**, *4* (8), 3125–3134. https://doi.org/10.1021/jacsau.4c00449.
(19) Coscia, F.; Doll, S.; Bech, J. M.; Schweizer, L.; Mund, A.; Lengyel, E.; Lindebjerg, J.; Madsen, G. I.; Moreira, J. M.; Mann, M. A Streamlined Mass Spectrometry–Based Proteomics Workflow for Large-Scale FFPE Tissue Analysis. *J. Pathol.* **2020**, *251* (1), 100–112. https://doi.org/10.1002/path.5420.
(20) Gillaspy, A. F.; Worrell, V.; Orvis, J.; Roe, B. A.; Dyer, D. W.; Iandolo, J. J. The Staphylococcus Aureus NCTC 8325 Genome. In *Gram-Positive Pathogens*; John Wiley & Sons, Ltd, 2006; pp 381–412. https://doi.org/10.1128/9781555816513.ch32.

5 Appendix

5.1 List of Abbreviations

ABPP	Activity-based Protein Profiling
AGC	Automatic gain control
APCI	Atmospheric-pressure chemical ionization
BCA	Bicinchoninic Acid
BHT	Butylated hydroxytoluene
BPO	Benzoyl peroxide
calcd.	calculated
CAM	Cerium Ammonium Molybdate
Cbz	Benzyloxycarbonyl
CSA	Camphorsulfonic acid
Ctrl.	Control
DBE	1,2-Dibromoethane
DCM	Dichloromethane
DDA	Data-dependent acquisition
ddH2O	Double-distilled (ultrapure) water
DIA	Data-independent acquisition
dia-PASEF	Data-independent acquisition – parallel accumulation-serial fragmentation
DIPEA	*N*,*N*-Diisopropylethylamin
DMAP	4-(Dimethylamino)pyridine
DMEM	Dulbecco's Modified Eagle Medium
DMF	Dimethylformammide
DMSO	Dimethyl sulfoxide
DNAse	Deoxyribonuclease
DTT	Dithiothreitol
E. coli	*Escherichia coli*
EI	Electron ionization
ESI	Electrospray ionization
eq.	Equivalent(-s)

FA	Formic acid
FBS	Fetal bovine serum
FDR	False discovery rate
GC-MS	Gas chromatography – Mass spectrometry
HCD	Higher-energy collision-induced dissociation
HEK 293	Human embryonic kidney 293 cells
HPLC	High-performance liquid chromatography
HRMS	High-resolution mass spectrometry
IAA	Iodoacetamide alkyne
IC_{50}	Half maximal inhibitory concentration
IM	Ion mobility
isoDTB	Isotopically labelled desthiobiotin
isol.	isolated
LC-MS	Liquid chromatography – Mass spectrometry
LDA	Lithium diisopropylamide
LFQ	Label-free quantification
LRMS	Low-resolution mass spectrometry
MBR	Match between runs
*m*CPBA	*meta*-Chloroperoxybenzoic acid
MIC	Minimal inhibitory concentration
MikA	Microketide A
MOM	Methoxymethyl
MTT	3-(4,5-Dimethyl thiazol-2-yl)-2,5-diphenyltetrazolium bromide
NBS	*N*-Bromosuccinimide
NMO	*N*-Methylmorpholine *N*-oxide
NMP	*N*-Methyl-2-pyrrolidone
NMR	Nuclear magnetic resonance
OD_{600}	Optical density at a wavelength of 600 nm
PBS	Phosphate-buffered saline
PIFA	Phenyliodine bis(trifluoroacetate)
ppm	Parts per million
PSM	Peptide-spectrum match

RAM	Random access memory
r.t.	Room temperature
RT	Retention time
rel.	relative
R_f	Retention factor
S. aureus	*Staphylococcus aureus*
SDS	Sodium dodecyl sulfate
spec.	spectroscopic
std.	Standard deviation
TBAF	Tetrabutylammonium fluoride
TBTA	Tris((1-benzyl-4-triazolyl)methyl)amine
TCEP	Tris(2-carboxyethyl)phosphine
TEAB	Triethylammonium bicarbonate buffer
TFA	Trifluoroacetic acid
THF	Tetrahydrofuran
TLC	Thin layer chromatography
TMS	Trimethylsilyl
TMT	Tandem mass tag
UHPLC	Ultrahigh-performance liquid chromatography
UV	Ultraviolet radiation

5.2 NMR Spectra

5.2.1 ^{1}H NMR (400 MHz, $CDCl_3$) and ^{13}C NMR (101 MHz, $CDCl_3$) of compound 11

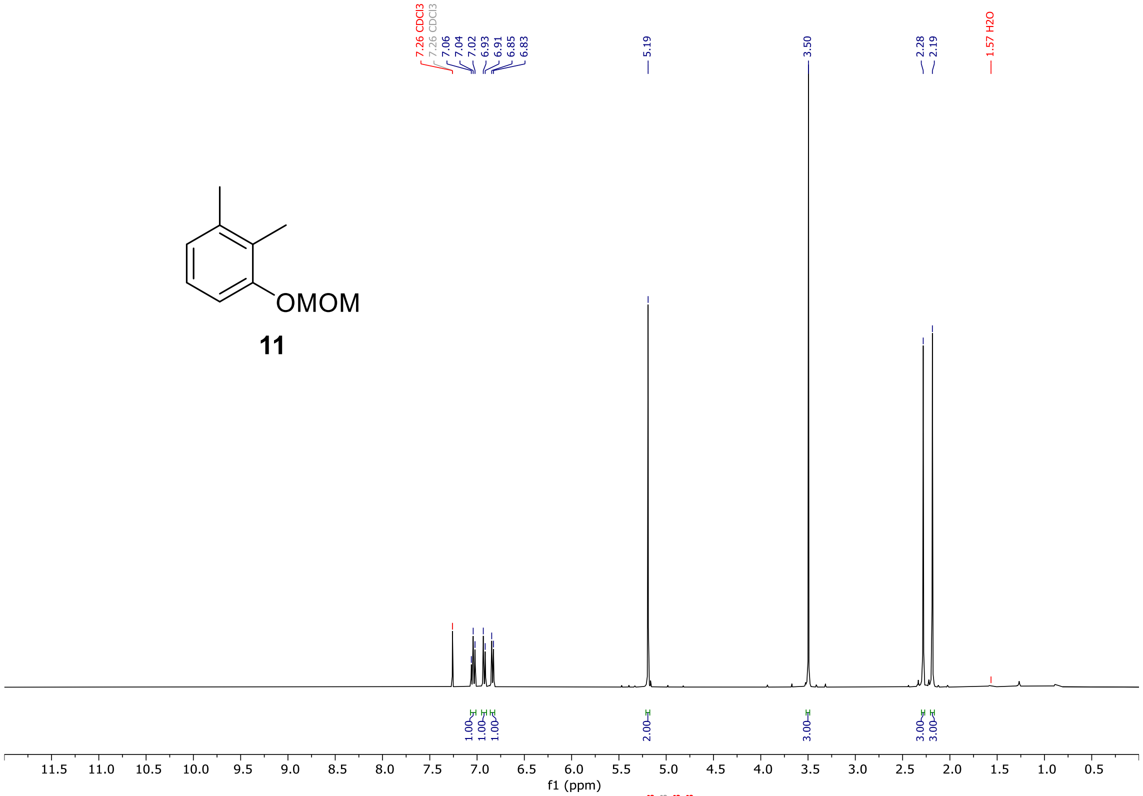

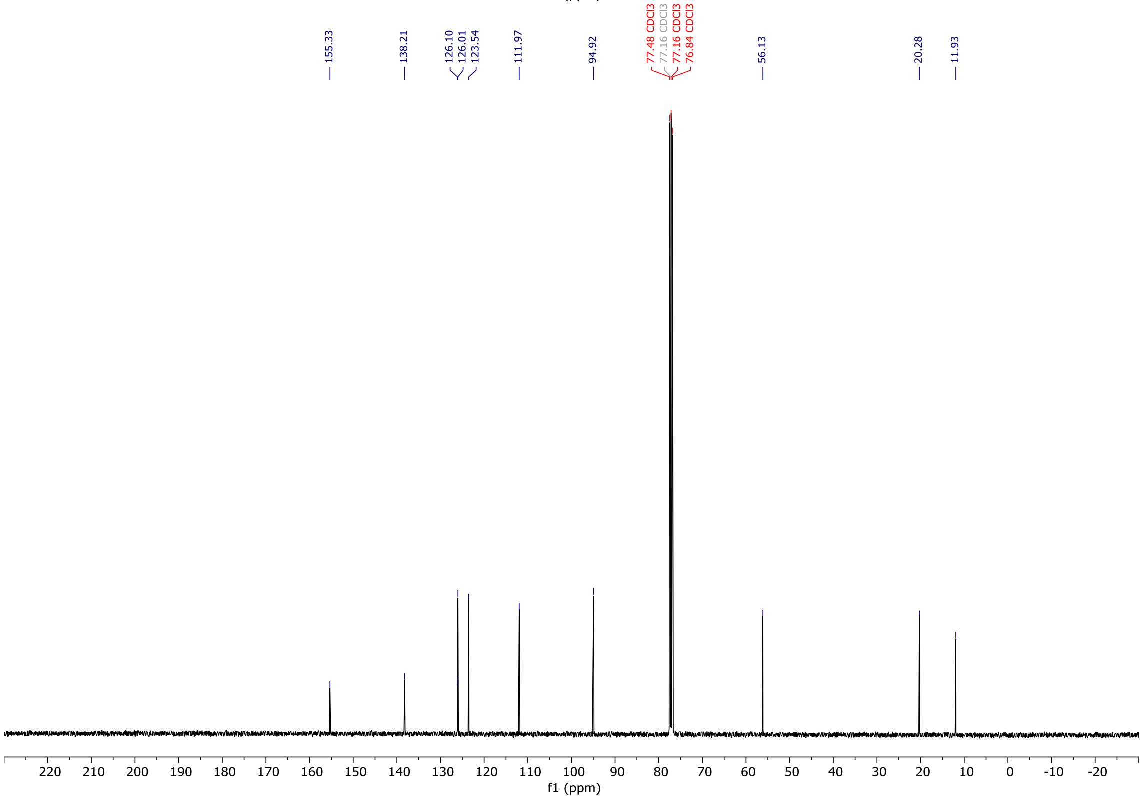

5.2.2 ^{1}H NMR (500 MHz, $CDCl_3$) and ^{13}C NMR (101 MHz, $CDCl_3$) of compound 6

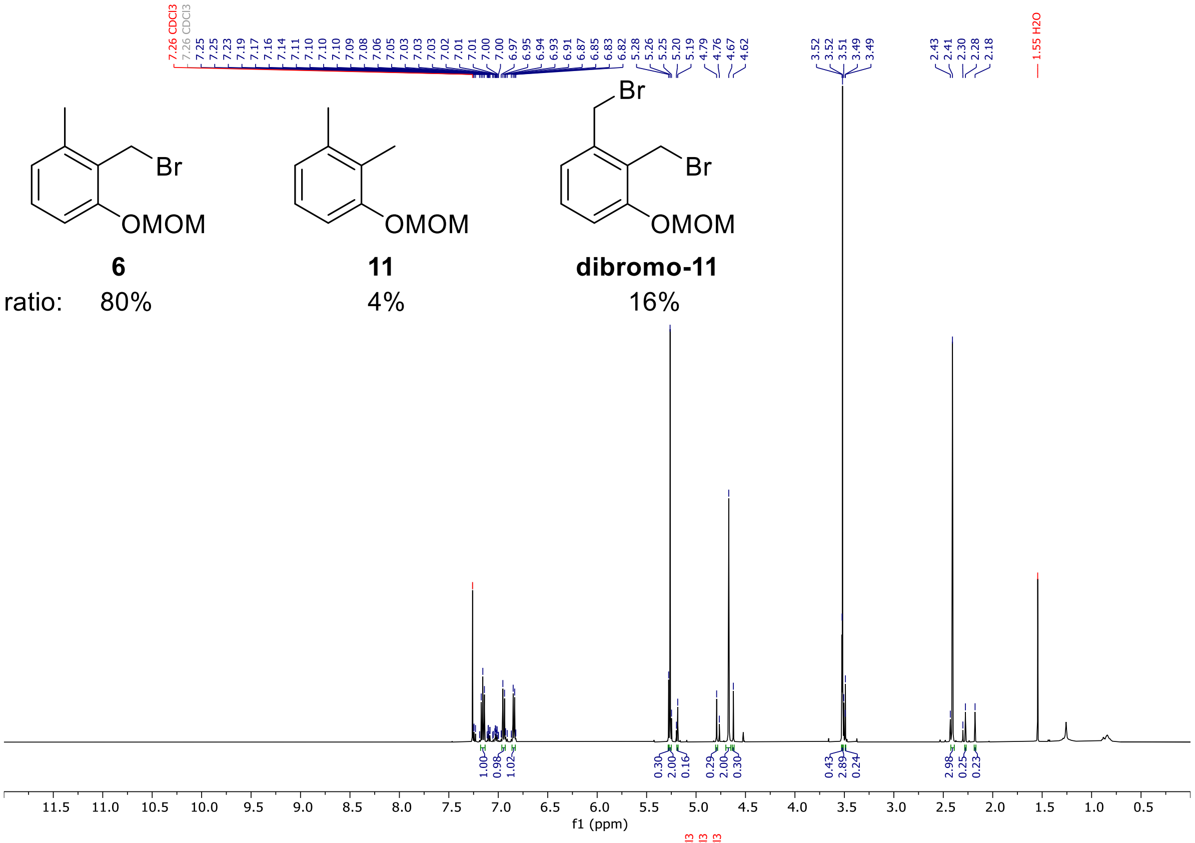

	6	11	dibromo-11
ratio:	80%	4%	16%

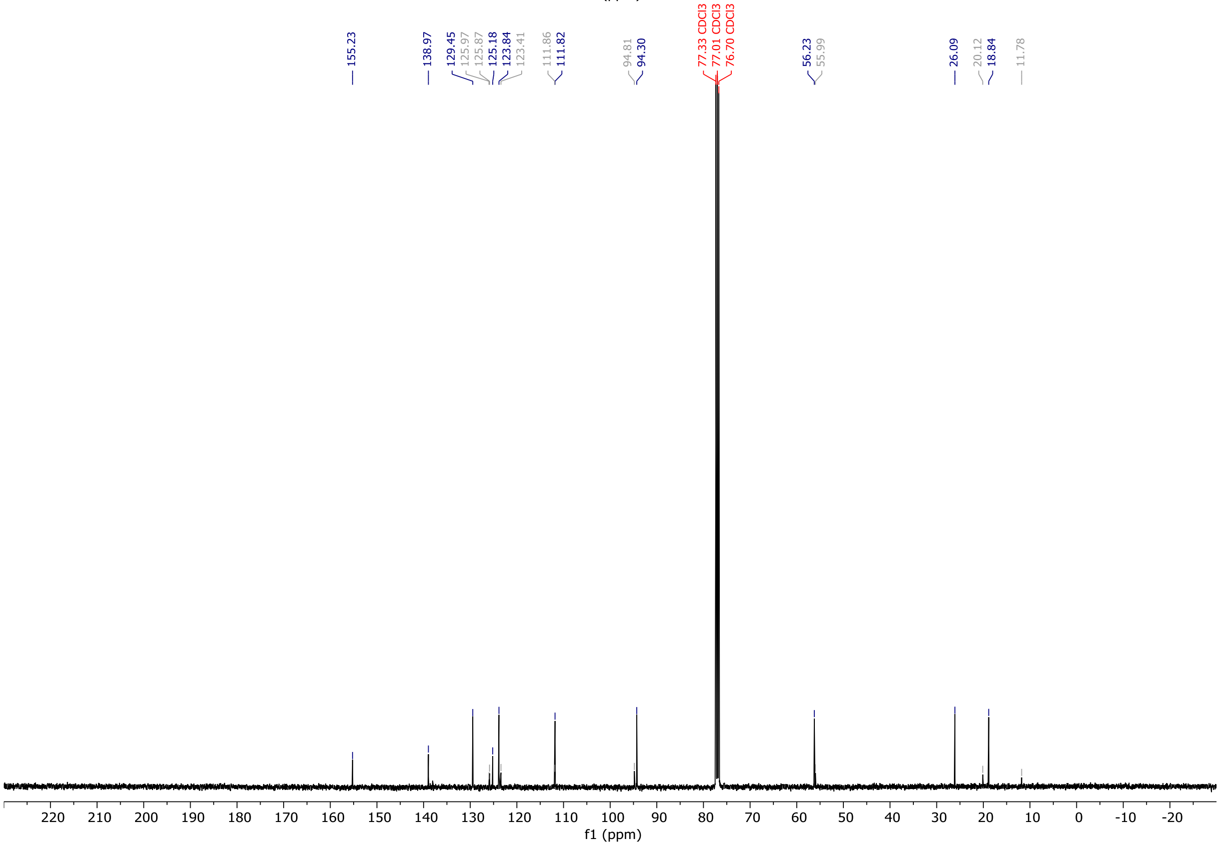

5.2.3 ^{1}H NMR (400 MHz, $CDCl_3$) and ^{13}C NMR (101 MHz, $CDCl_3$) of compound 13

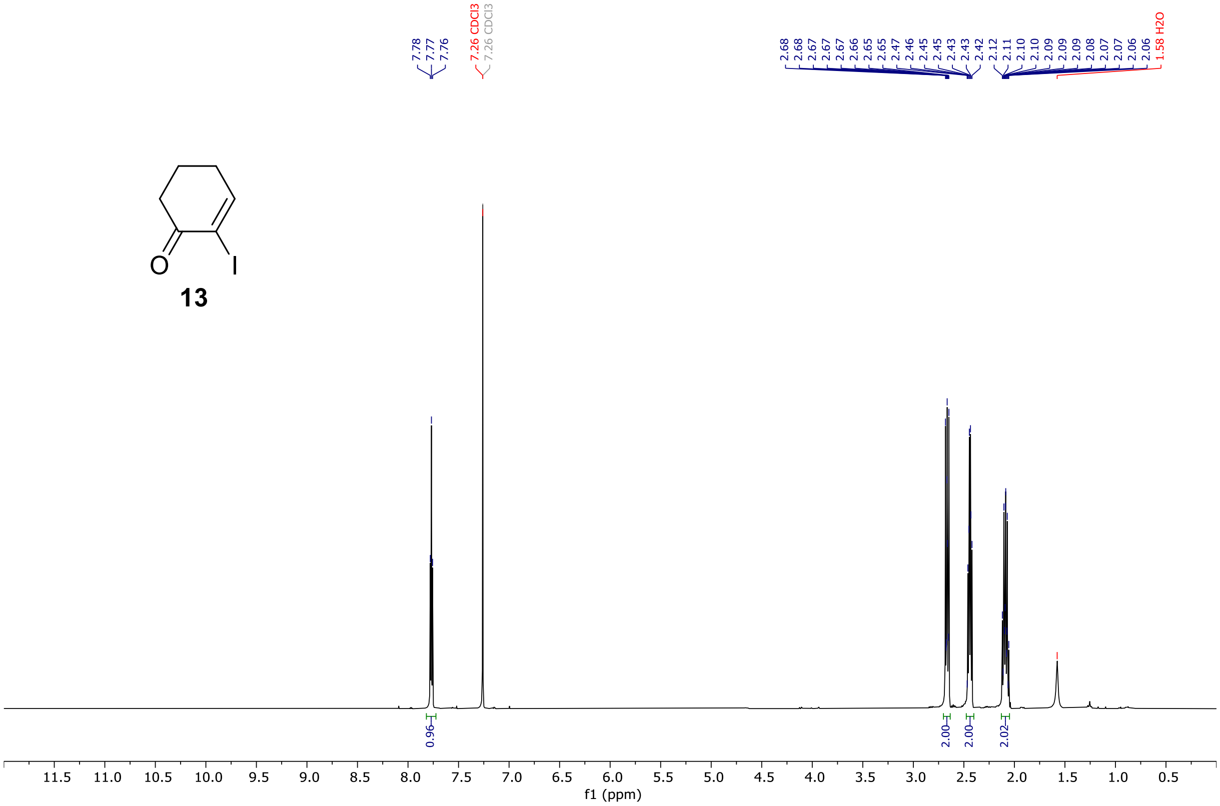

13

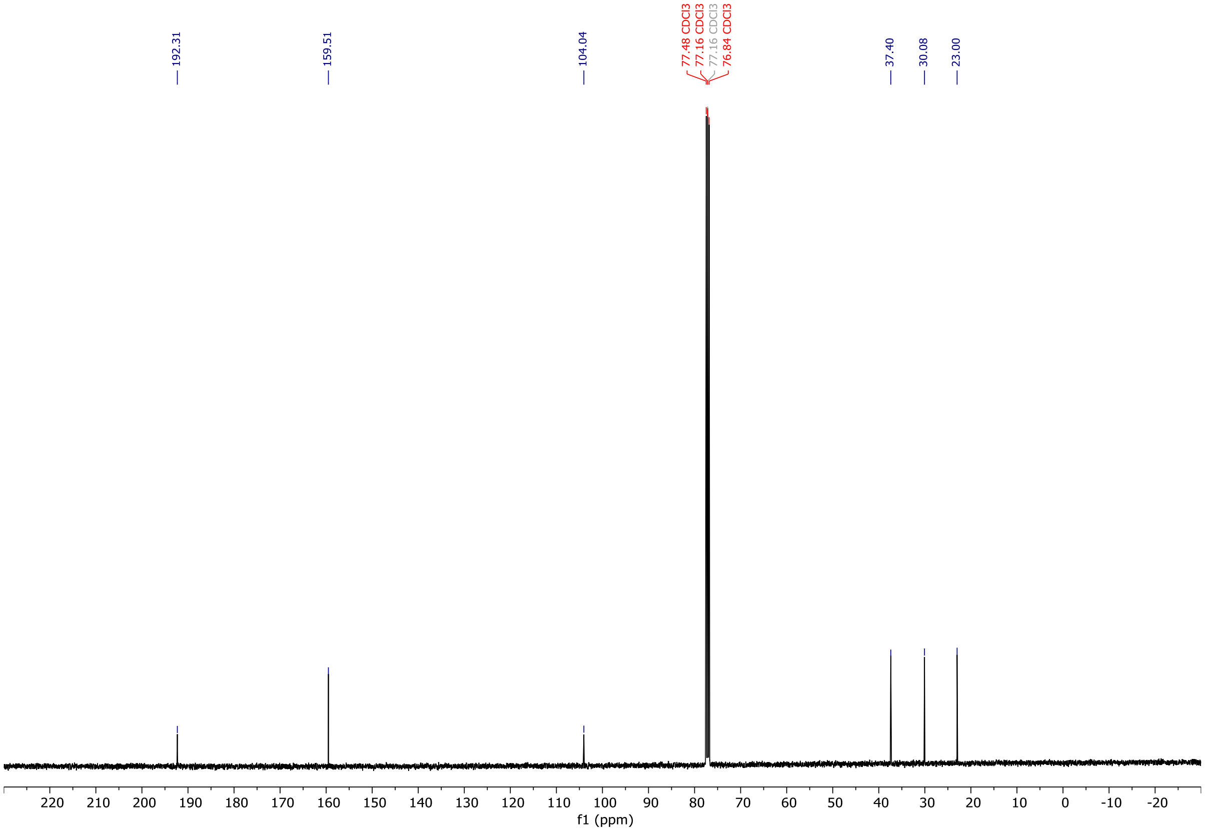

5.2.4 ^{1}H NMR (400 MHz, $CDCl_3$) and ^{13}C NMR (101 MHz, $CDCl_3$) of compound 14

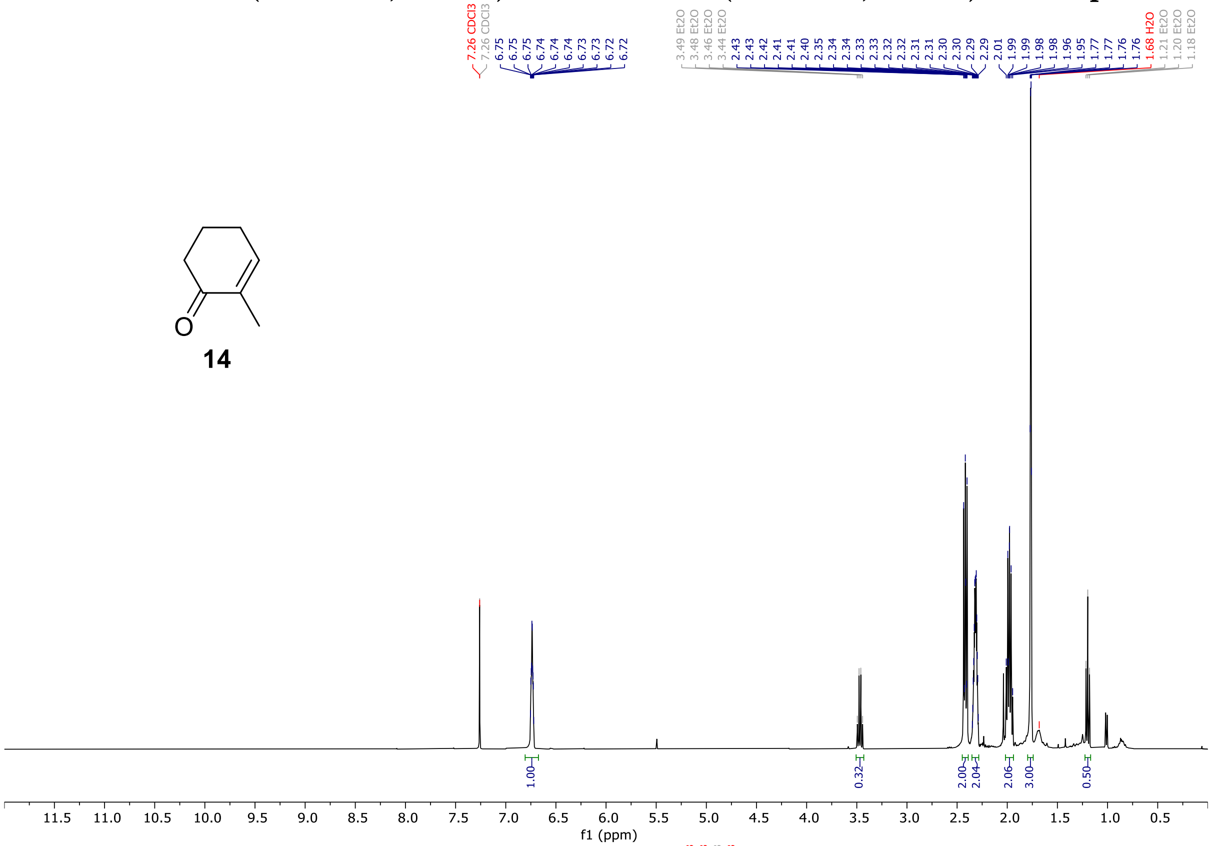

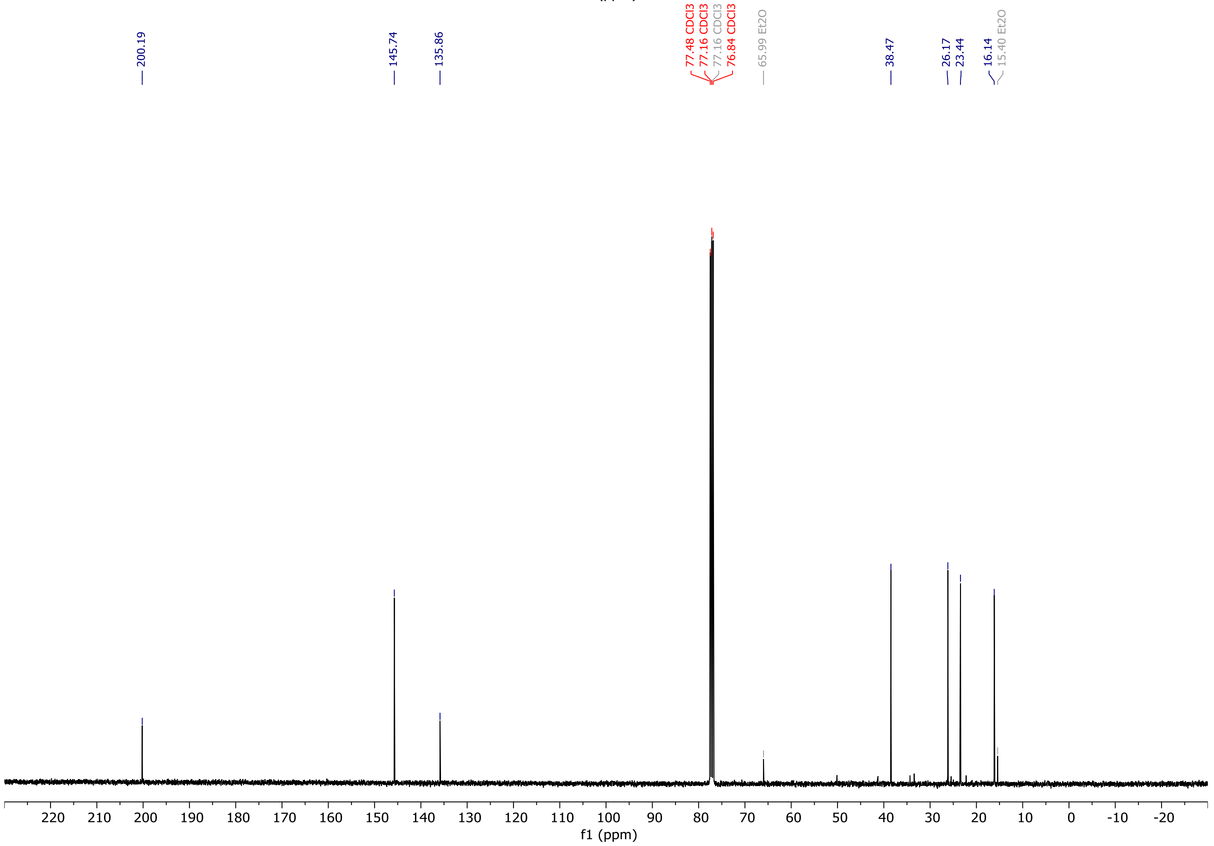

5.2.5 ^{1}H NMR (500 MHz, $CDCl_3$) and ^{13}C NMR (101 MHz, $CDCl_3$) of compound 15

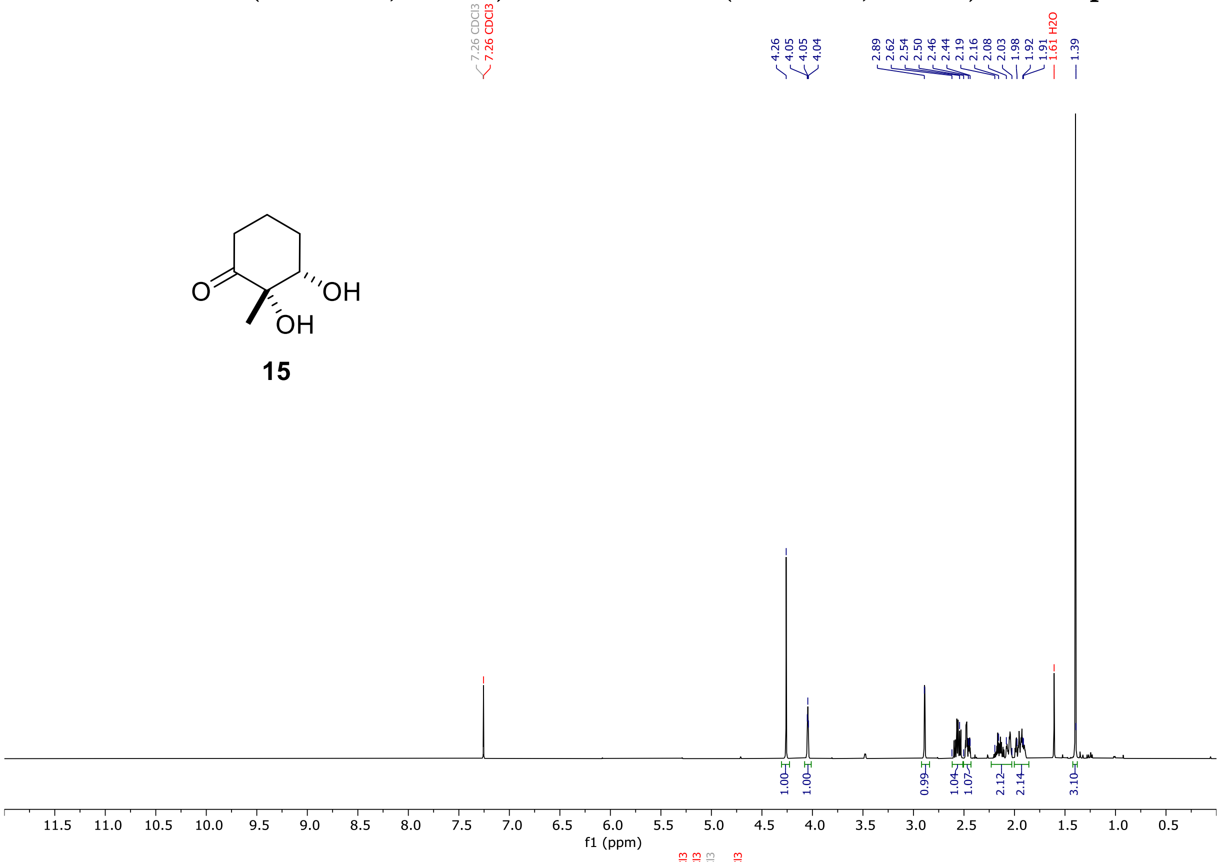

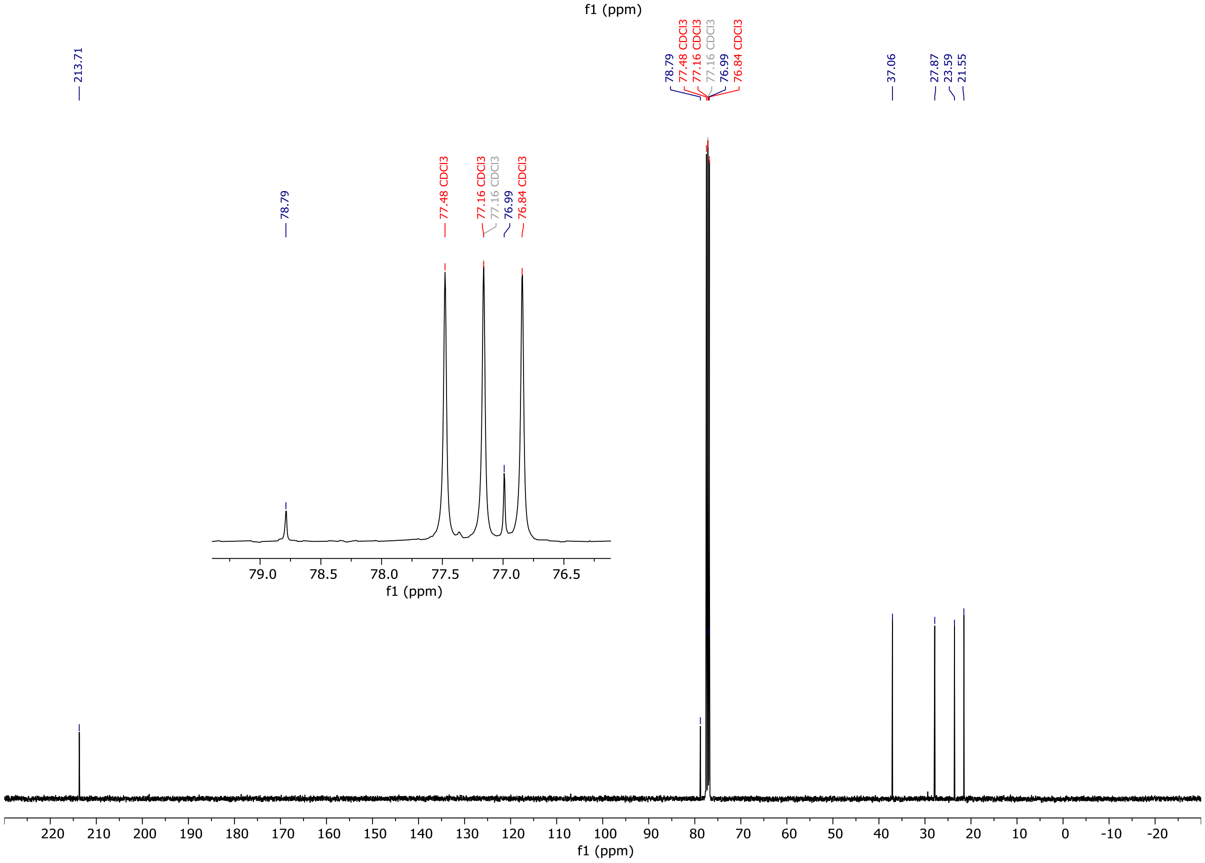

5.2.6 ^{1}H NMR (500 MHz, $CDCl_3$) and ^{13}C NMR (101 MHz, $CDCl_3$) of compound 9

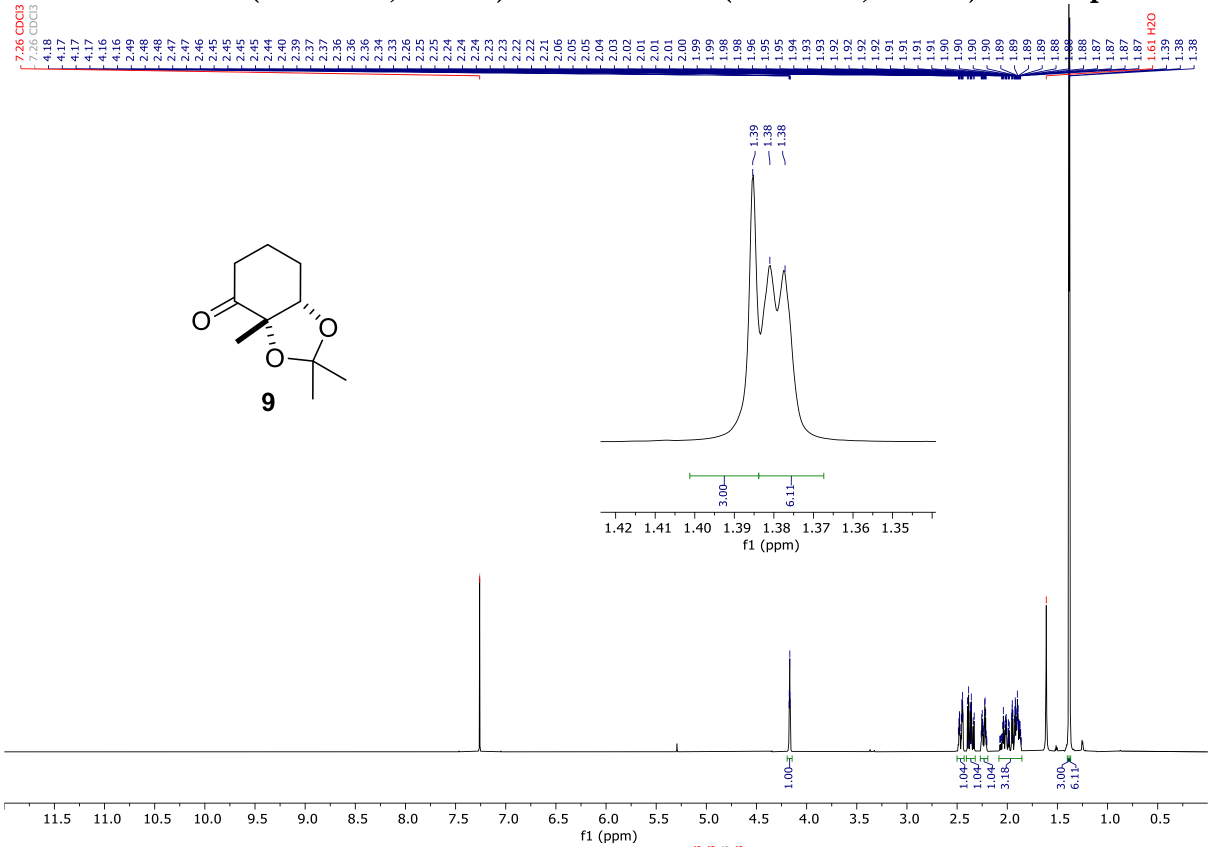

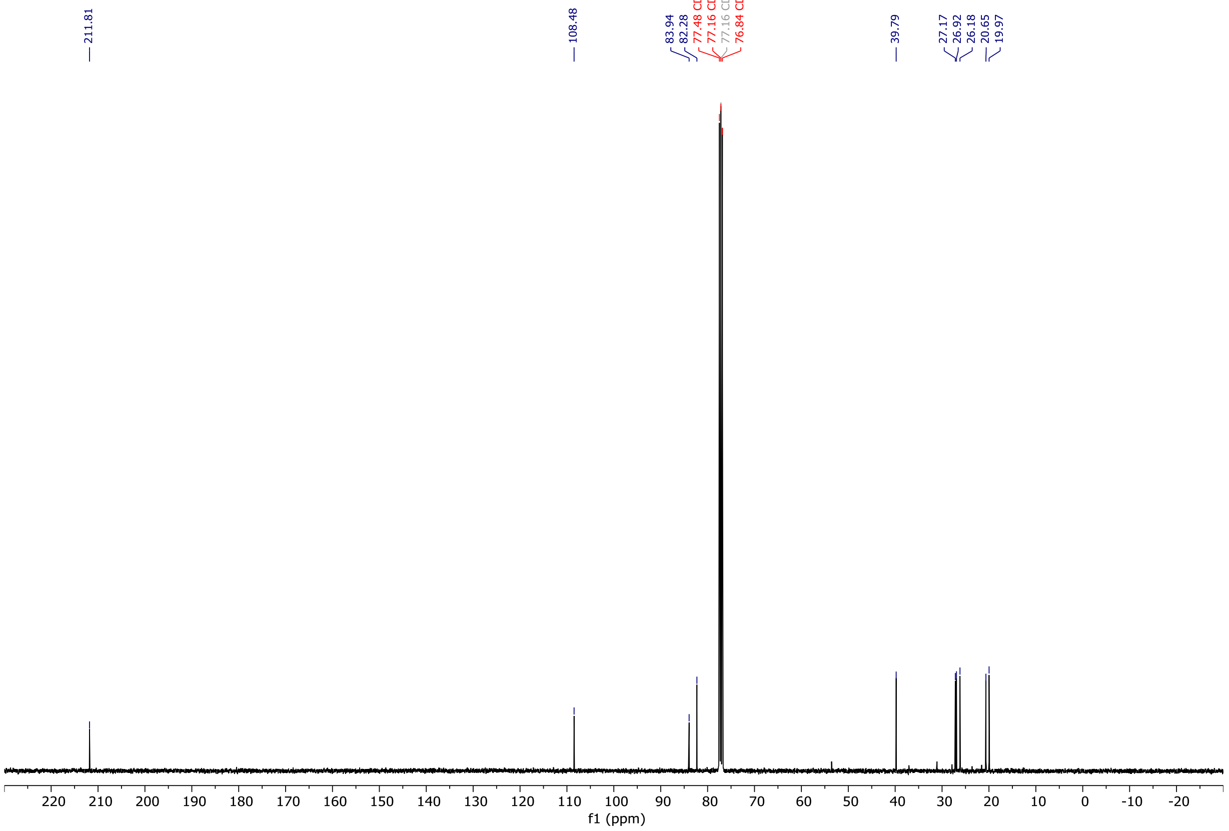

5.2.7 ^{1}H NMR (500 MHz, $CDCl_3$) and ^{13}C NMR (75 MHz, $CDCl_3$) of compound 16

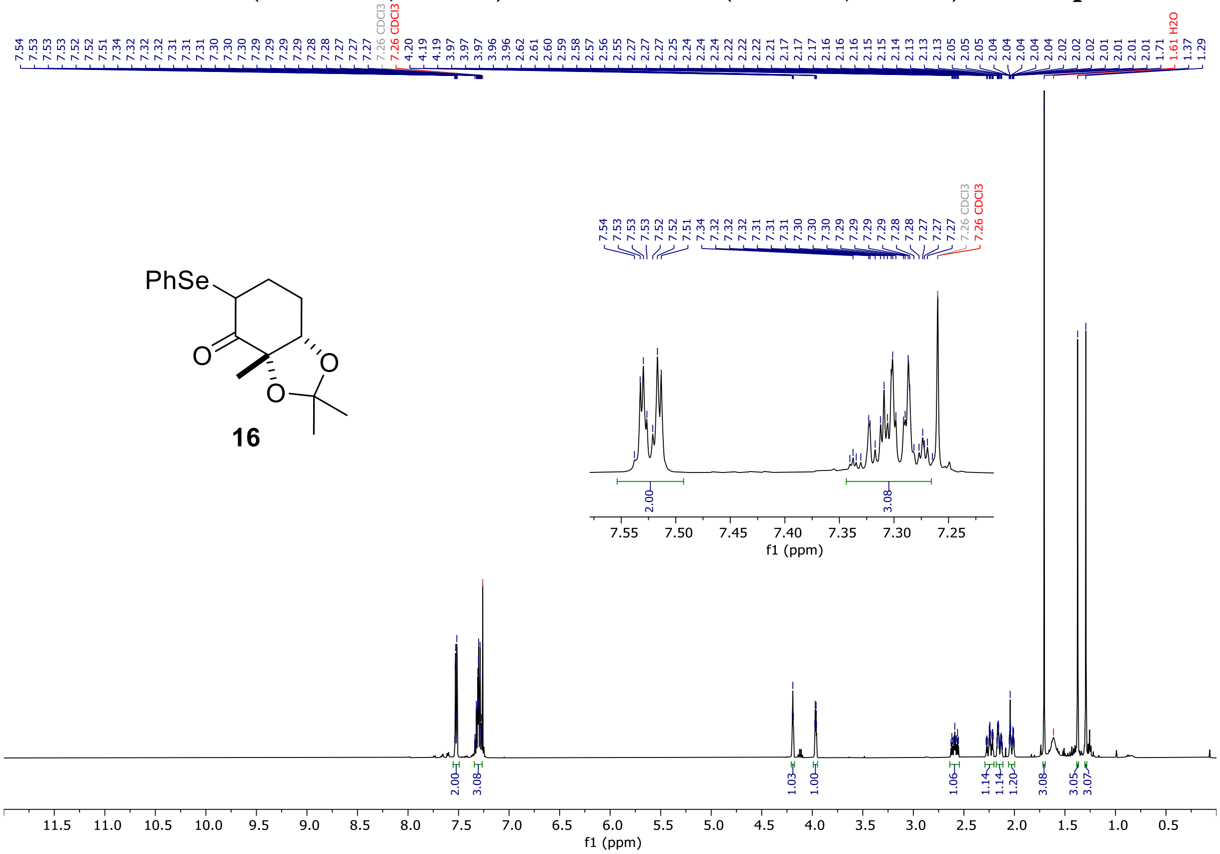

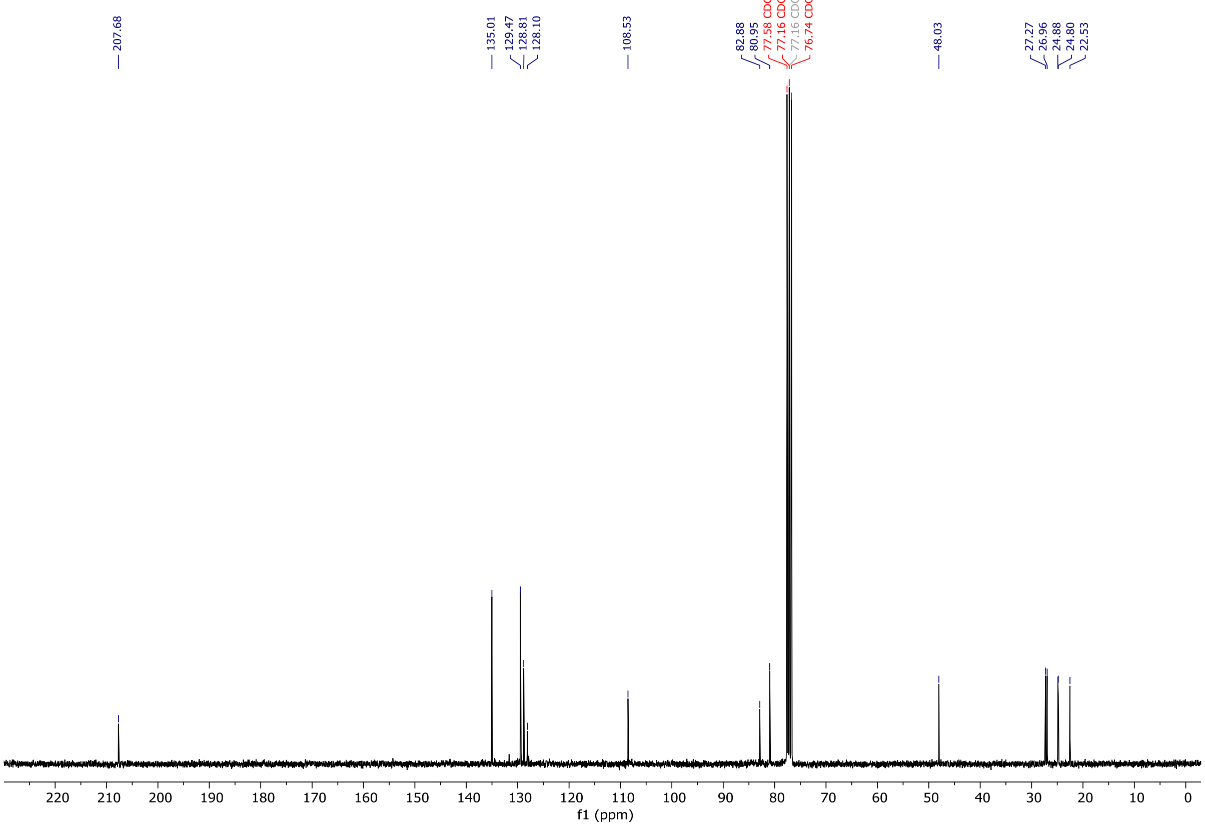

5.2.8 ^{1}H NMR (400 MHz, $CDCl_3$) and ^{13}C NMR (101 MHz, $CDCl_3$) of compound 17

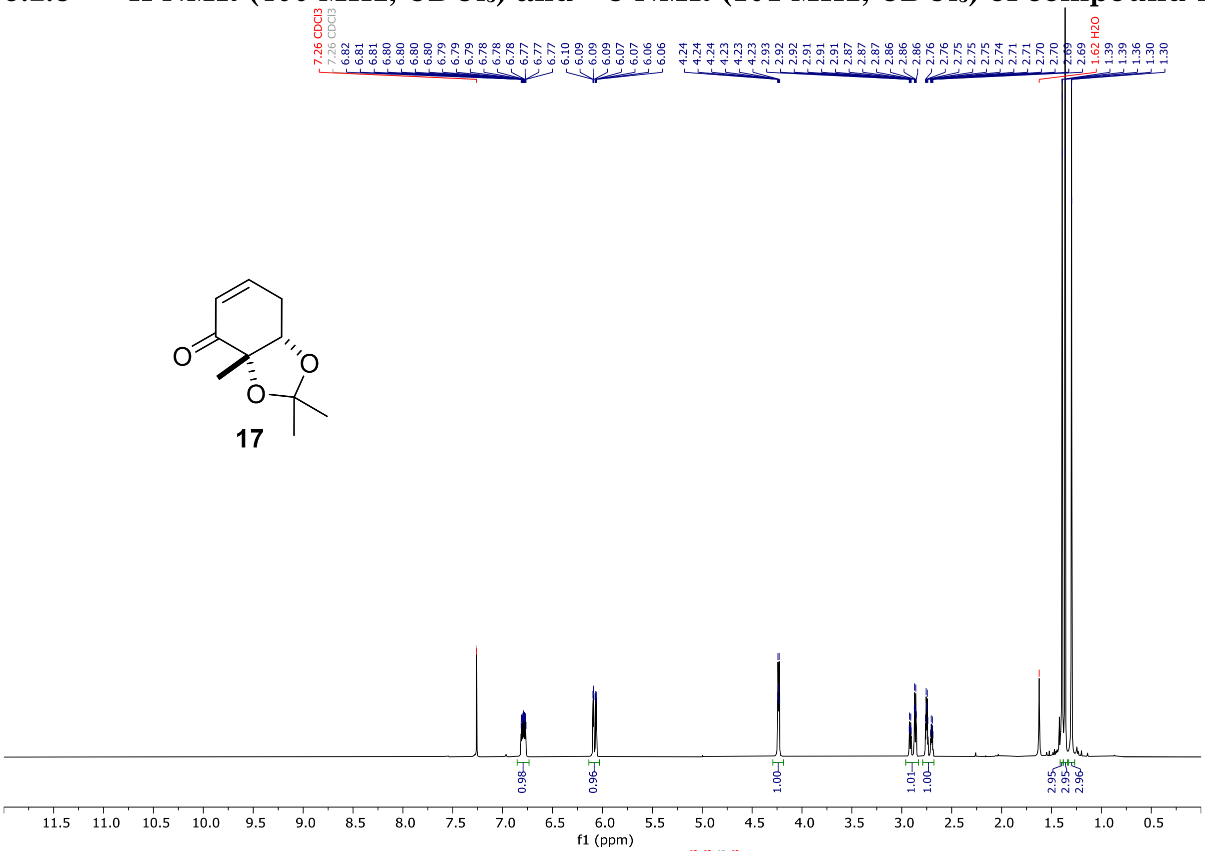

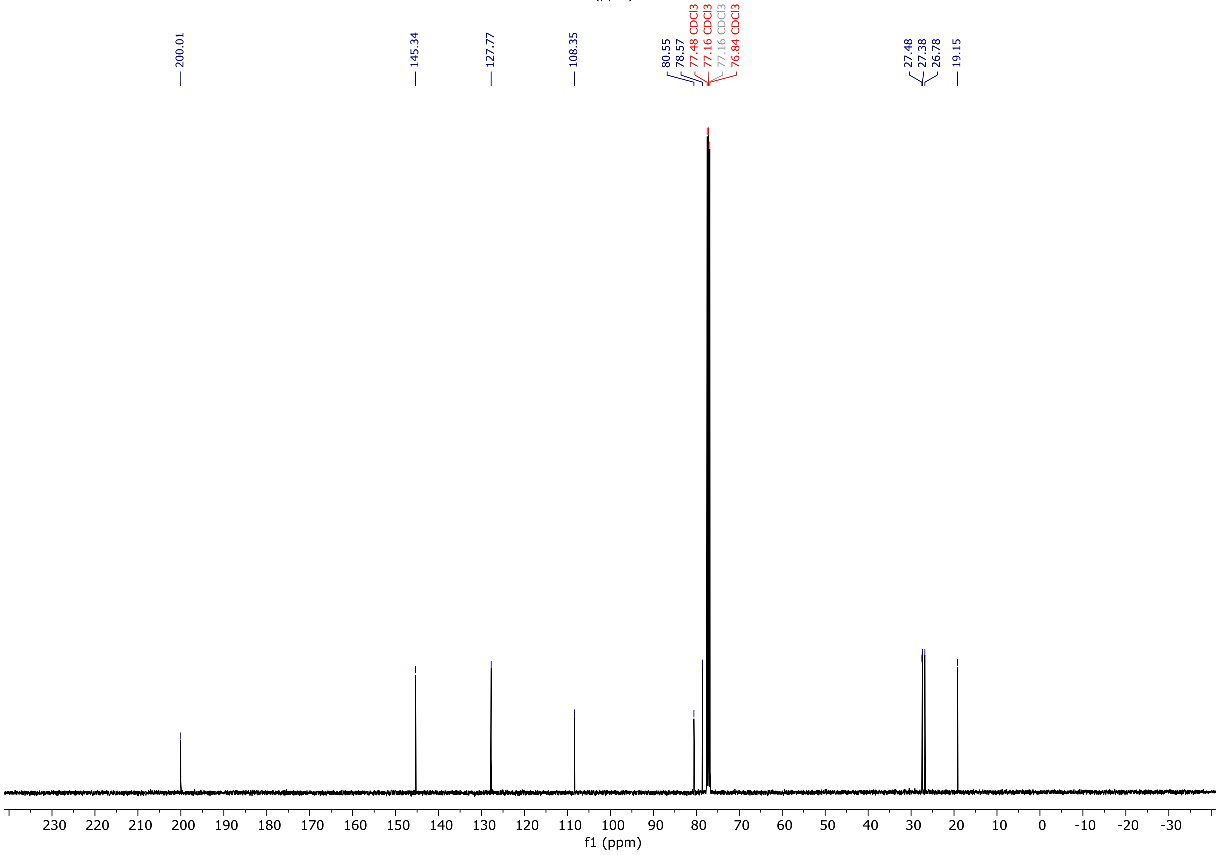

5.2.9 ^{1}H NMR (500 MHz, $CDCl_3$) and ^{13}C NMR (75 MHz, $CDCl_3$) of *syn*-8

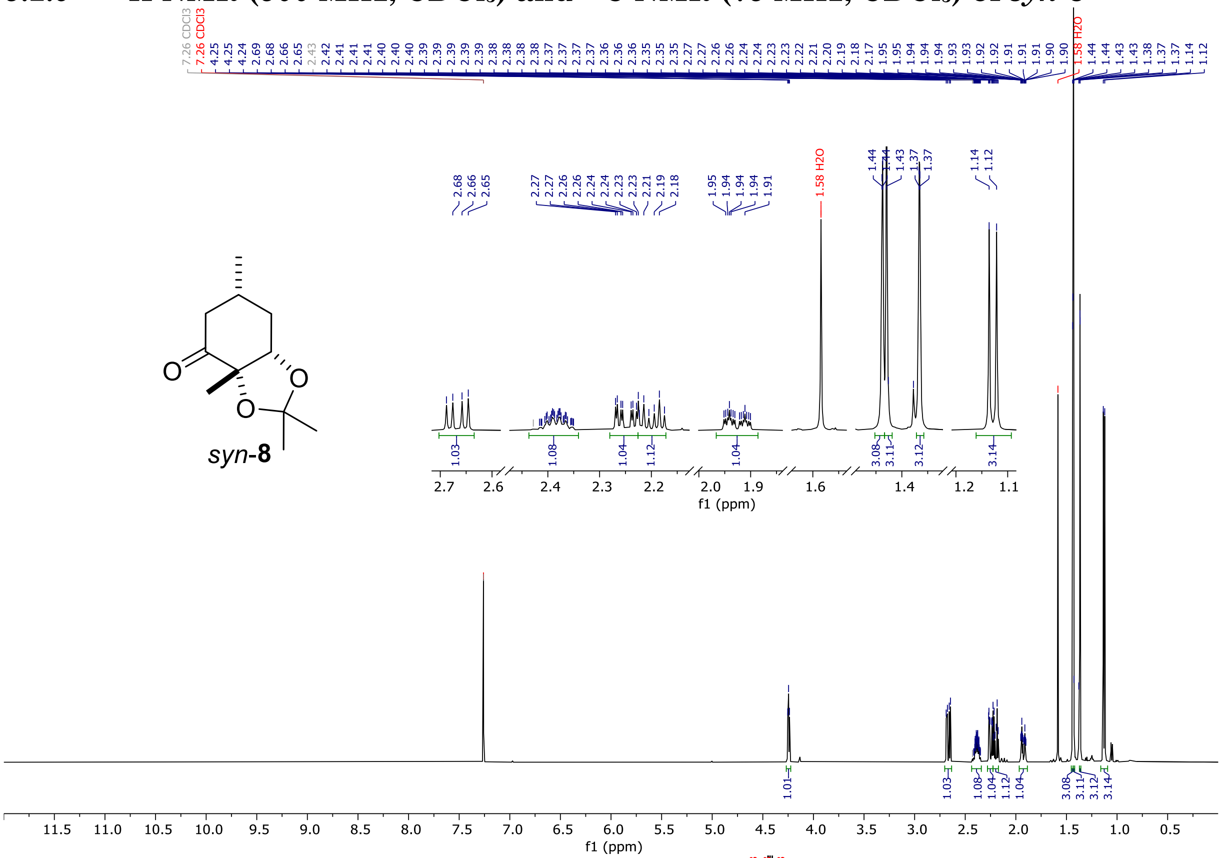

syn-**8**

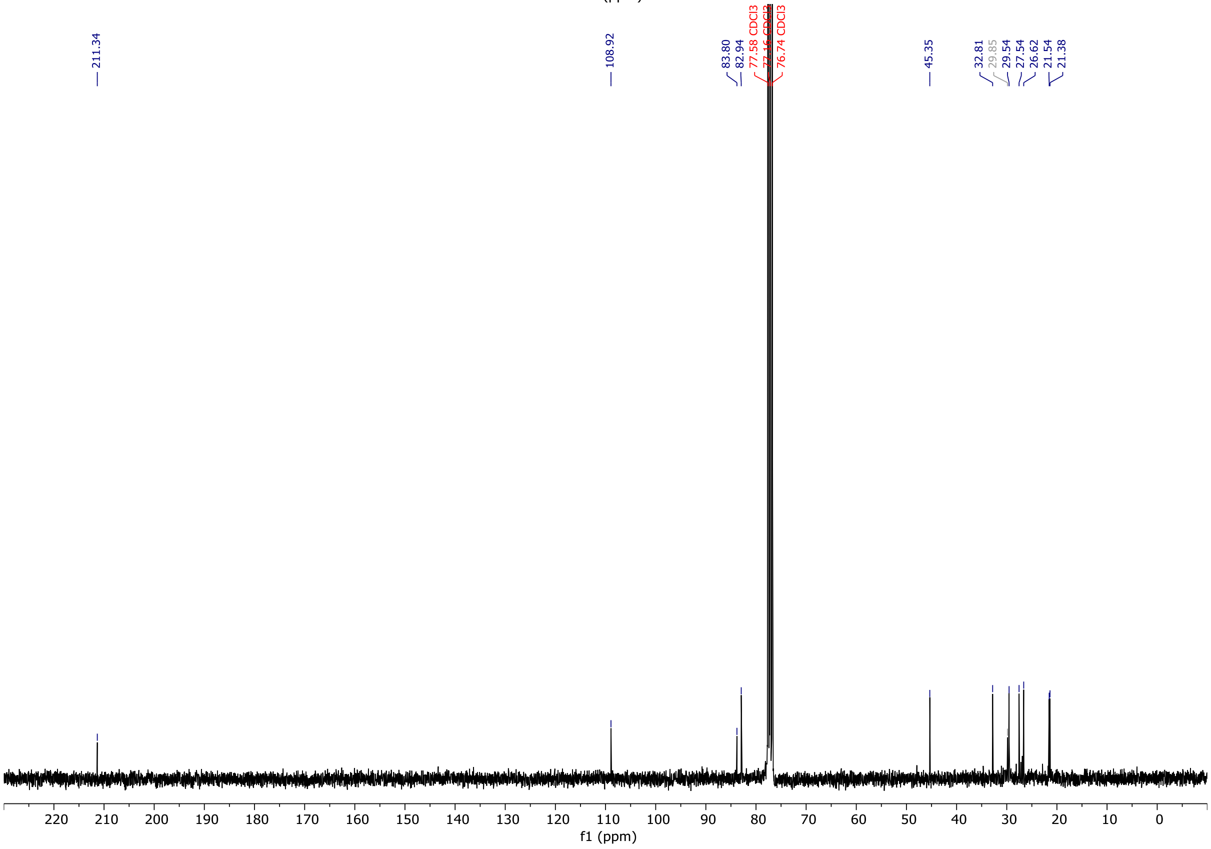

5.2.10 ^{1}H NMR (500 MHz, $CDCl_3$) and ^{13}C NMR (75 MHz, $CDCl_3$) of *anti*-8

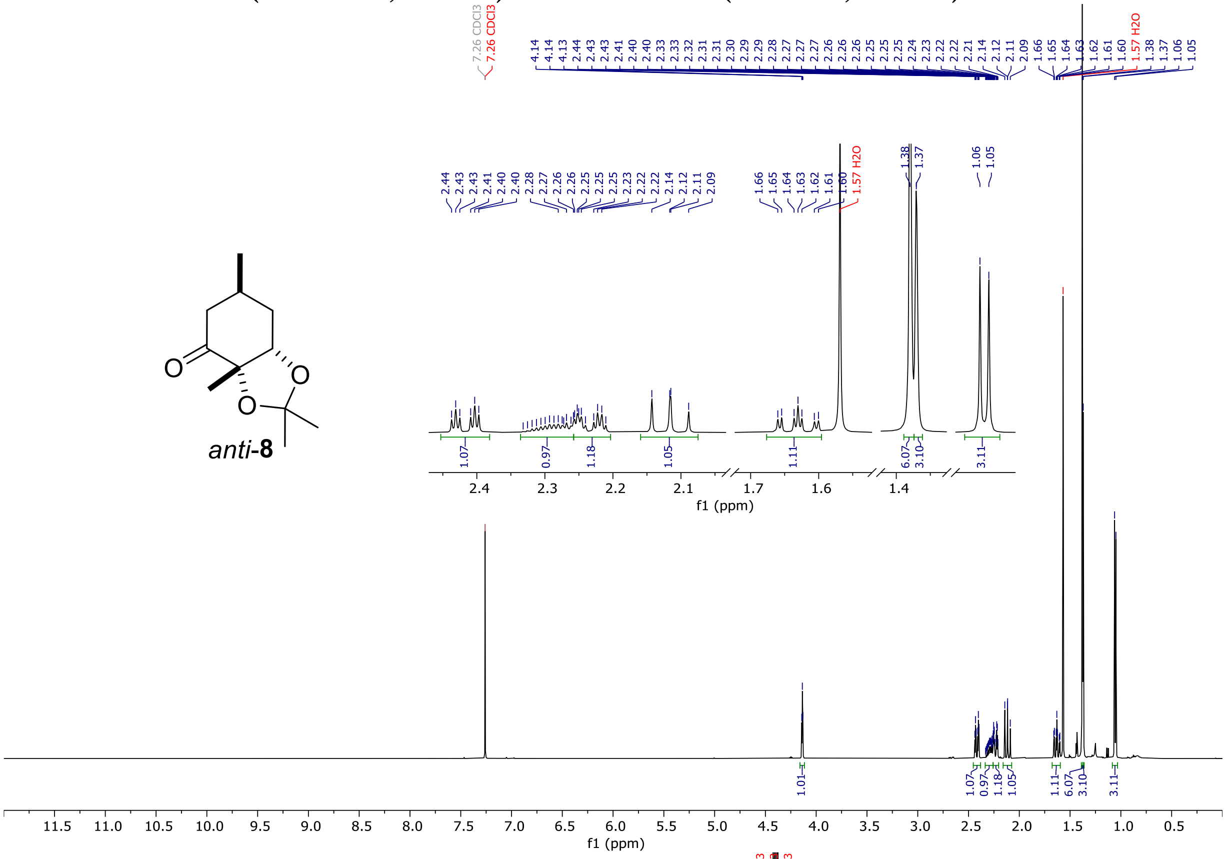

anti-**8**

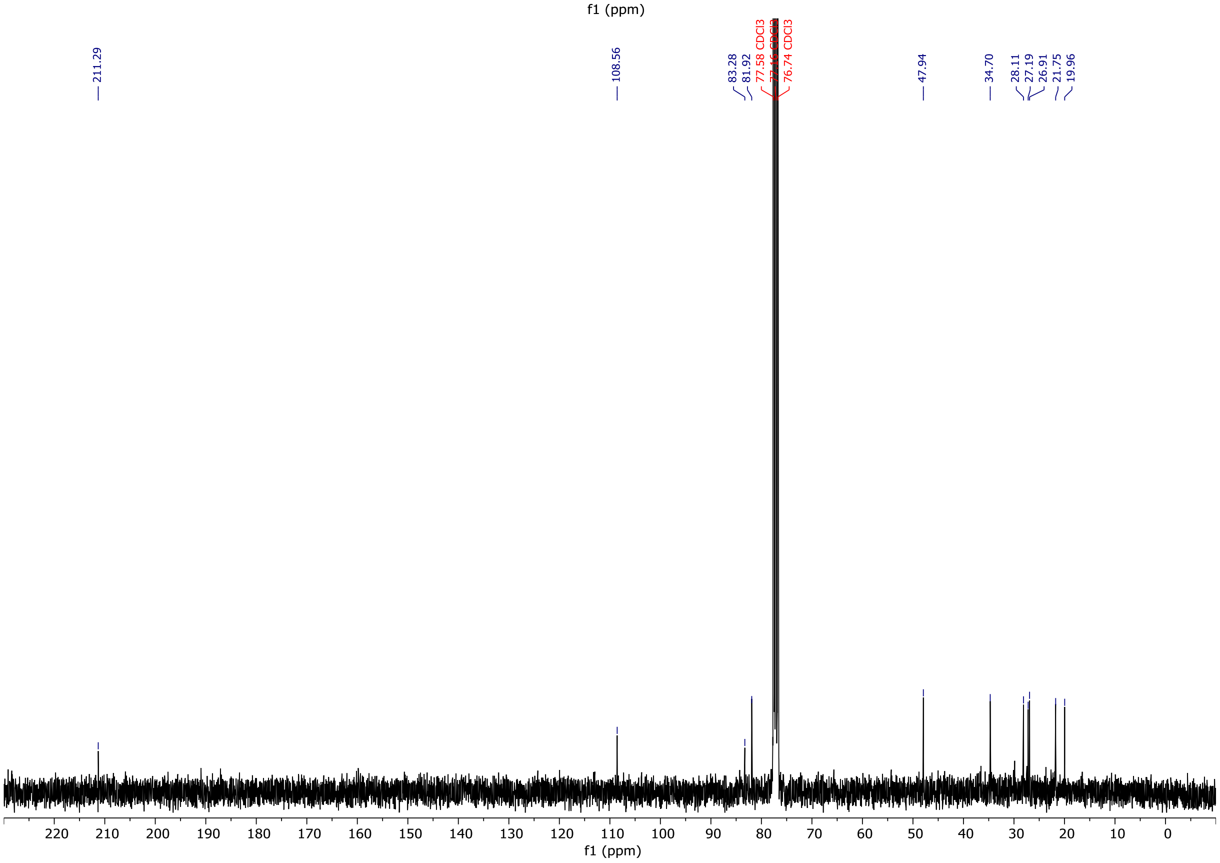

5.2.11 ^{1}H NMR (400 MHz, $CDCl_3$) and ^{13}C NMR (101 MHz, $CDCl_3$) of compound 7

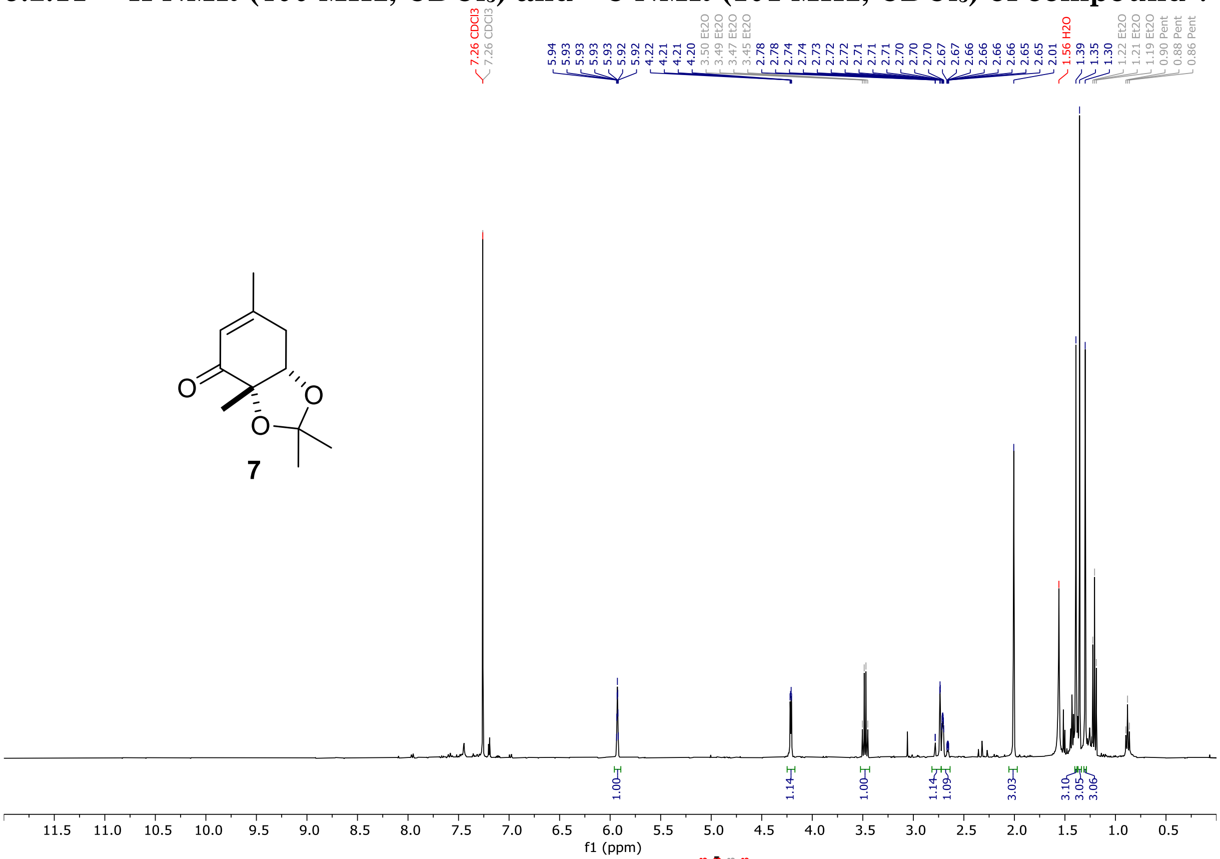

7

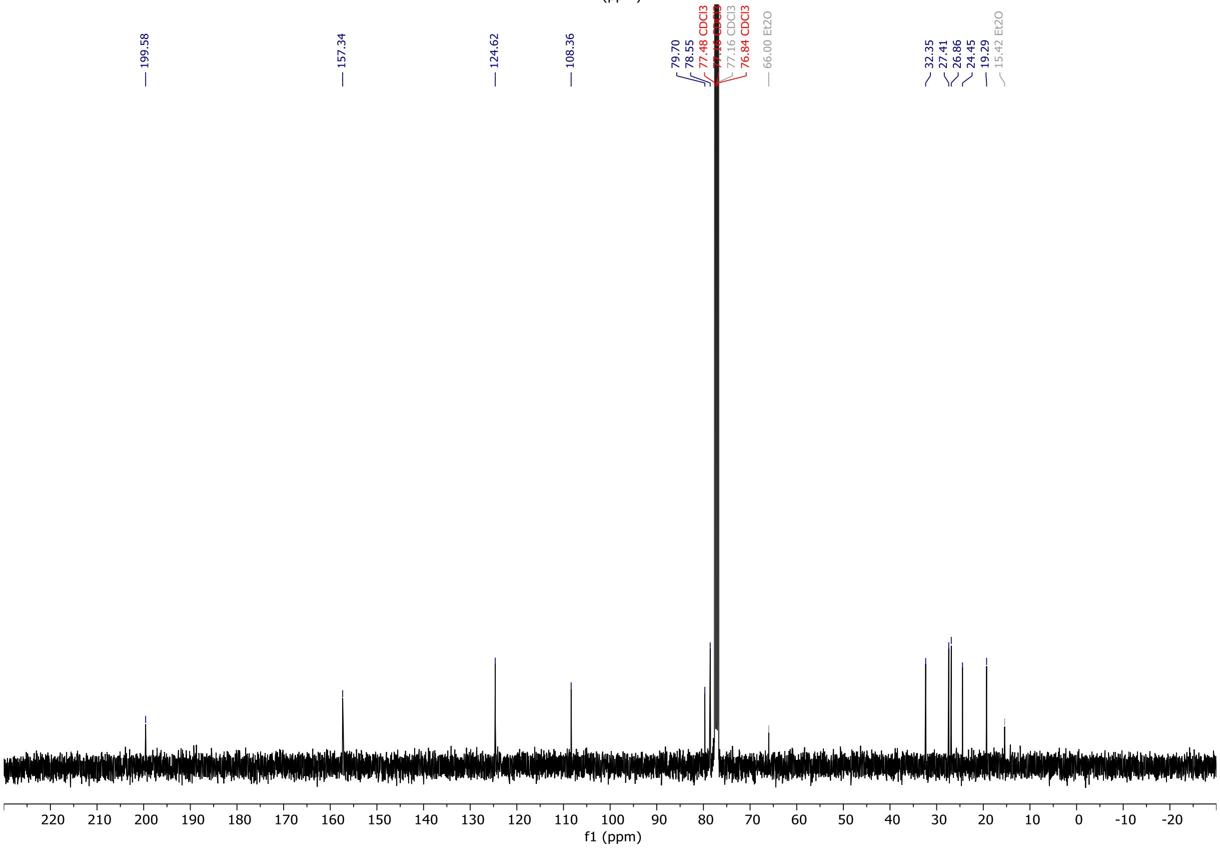

5.2.12 ^{1}H NMR (500 MHz, CD_3CN) of *rac*-leptosphaerone B (*rac*-4)

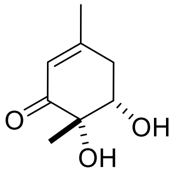

rac-**4**
rac-leptosphaerone B

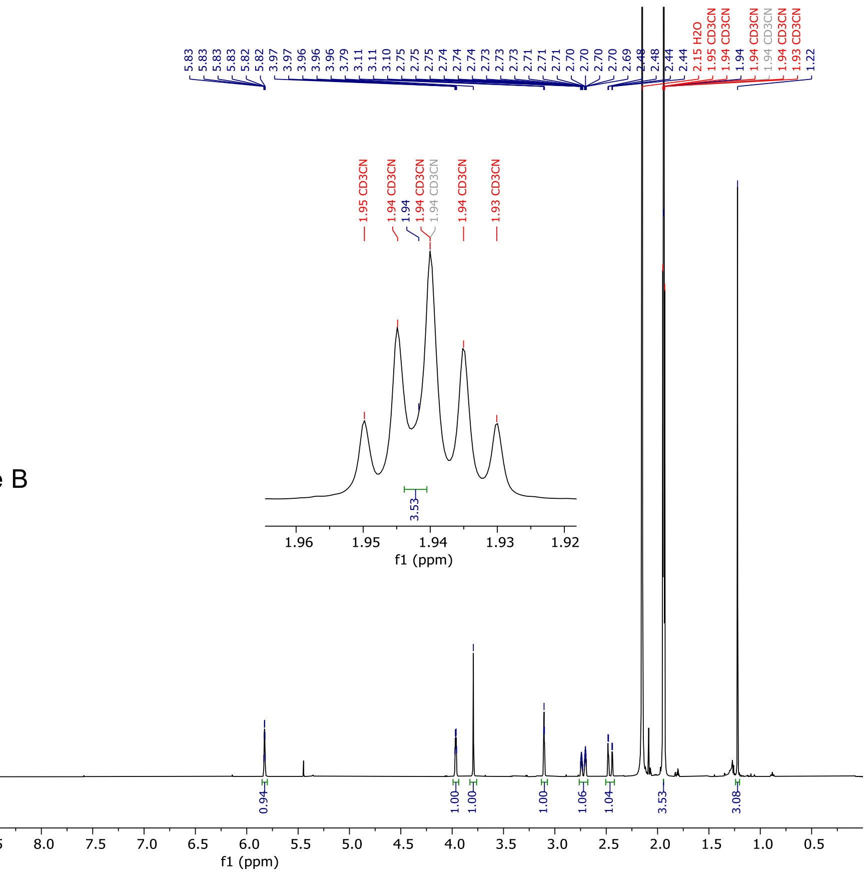

5.2.13 ^1H NMR (500 MHz, $CDCl_3$) and ^{13}C NMR (101 MHz, $CDCl_3$) of *syn*-35

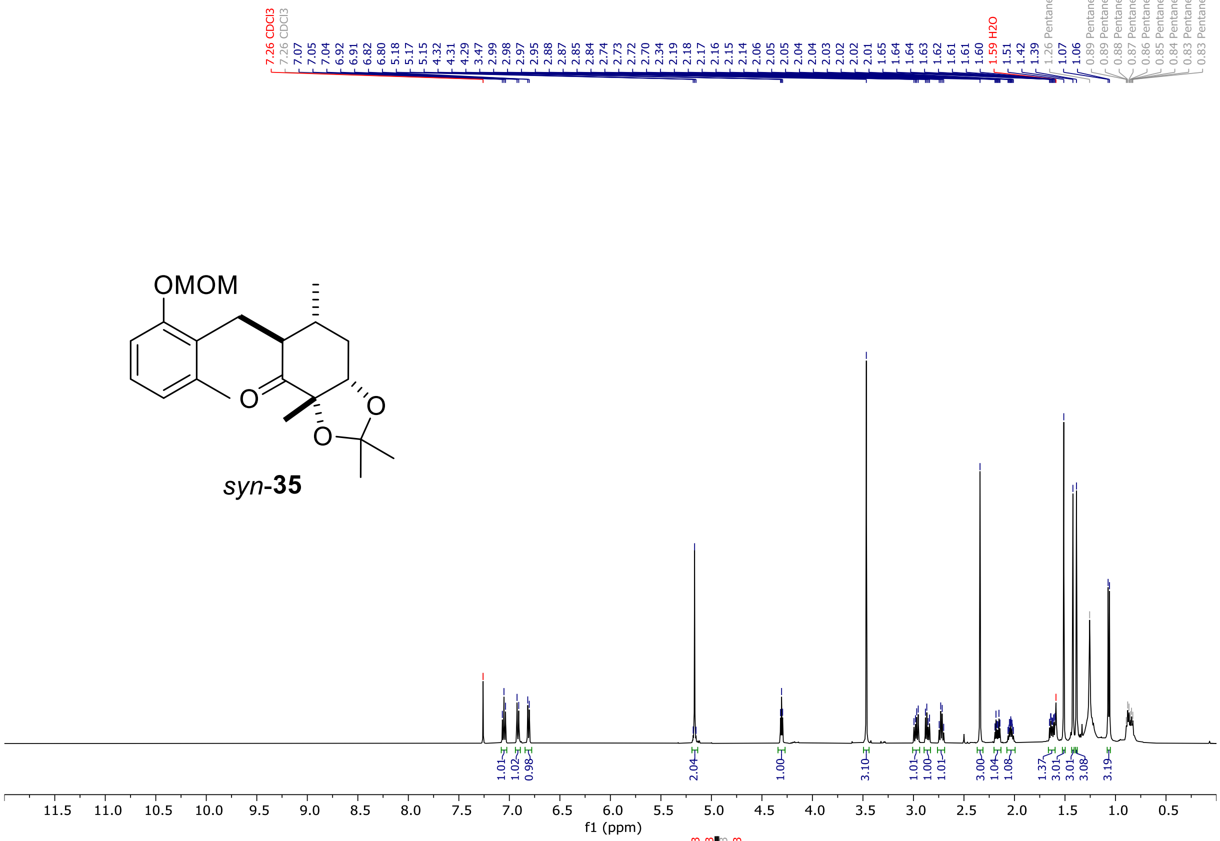

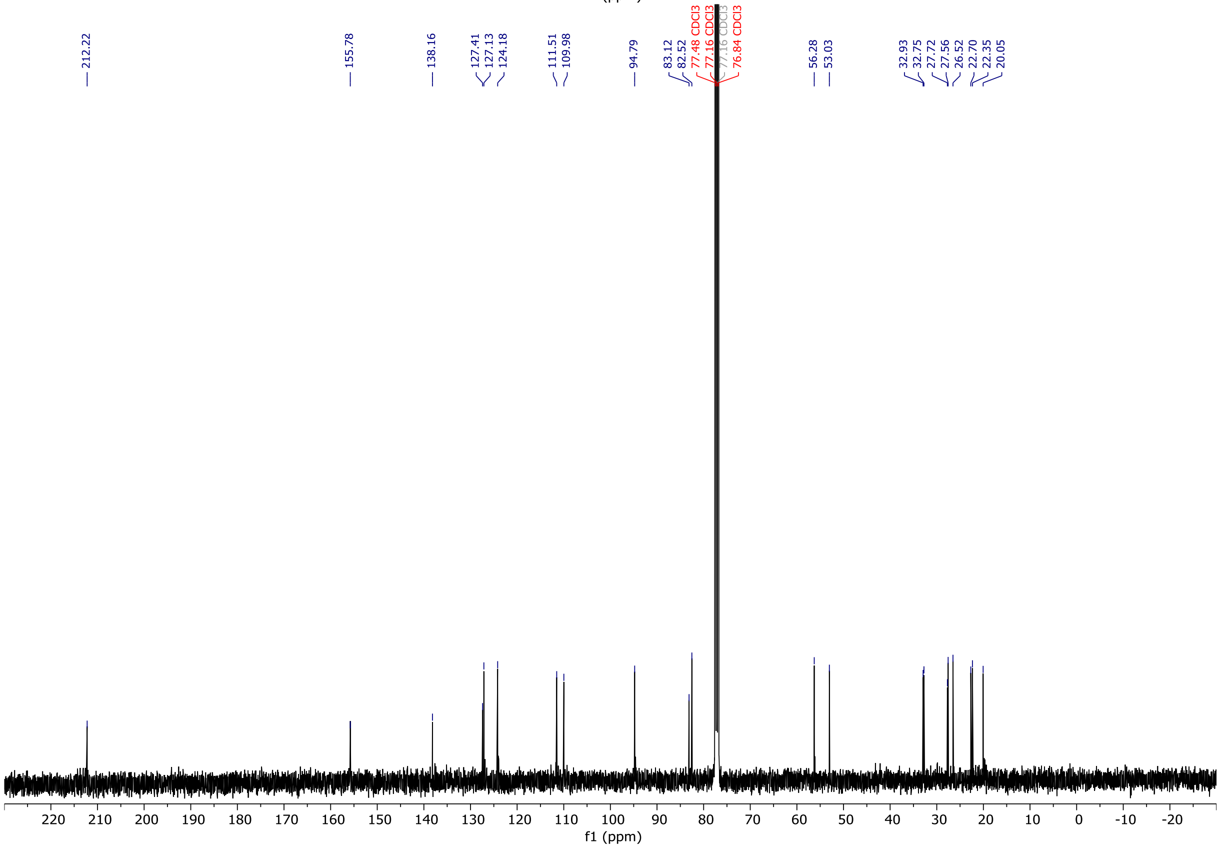

5.2.14 ^{1}H NOESY NMR (500 MHz, $CDCl_3$) of *syn*-35

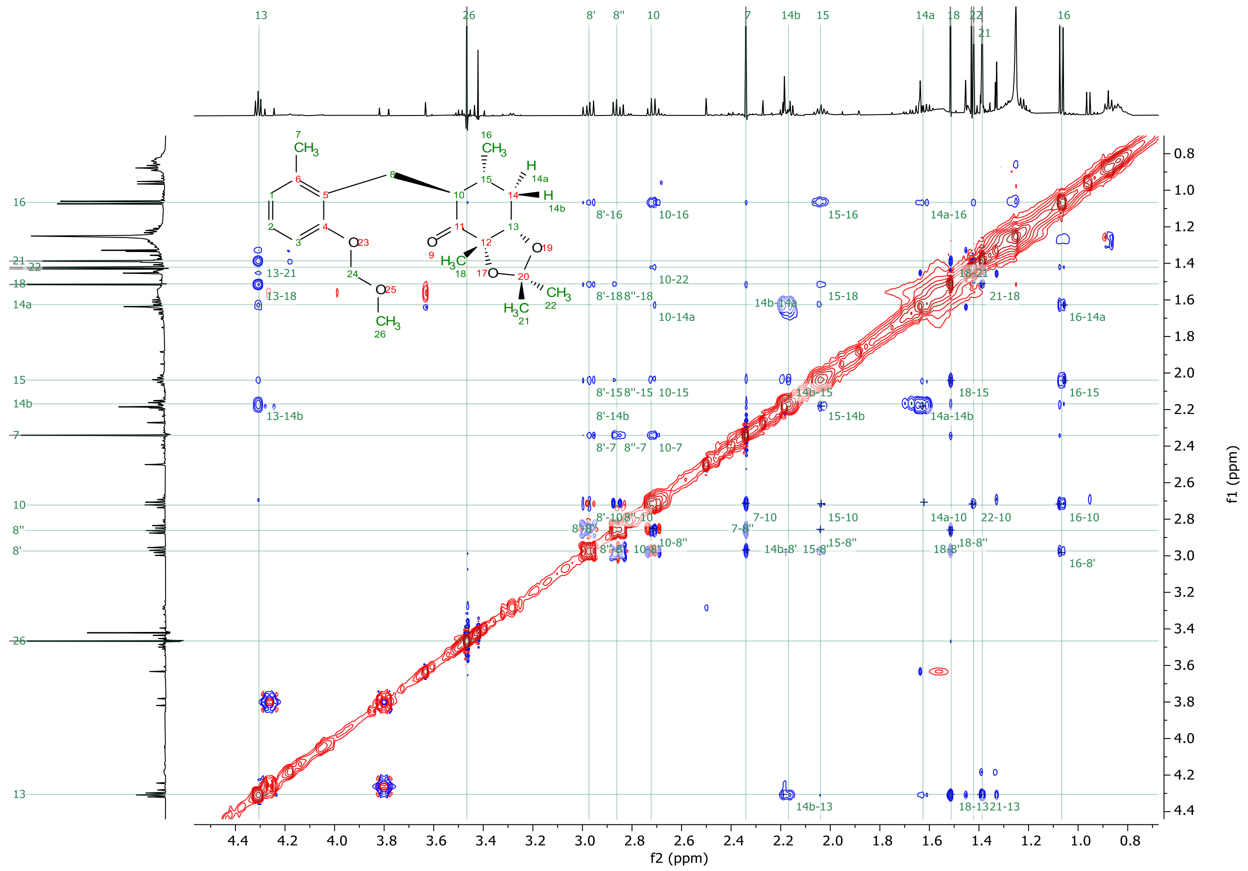

Relevant correlations for determination of the relative configuration at C-10 and C-15:

H-18 to H13, H-8, H15 and H-21

H-10 to H-14a, H-22 and H-16

5.2.15 ^{1}H NMR (400 MHz, $CDCl_3$) and ^{13}C NMR (101 MHz, $CDCl_3$) of *rac*-dihydro-MikA (38)

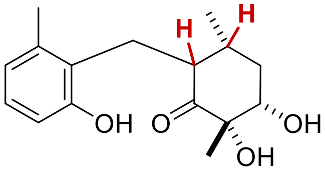

38
rac-dihydro-MikA

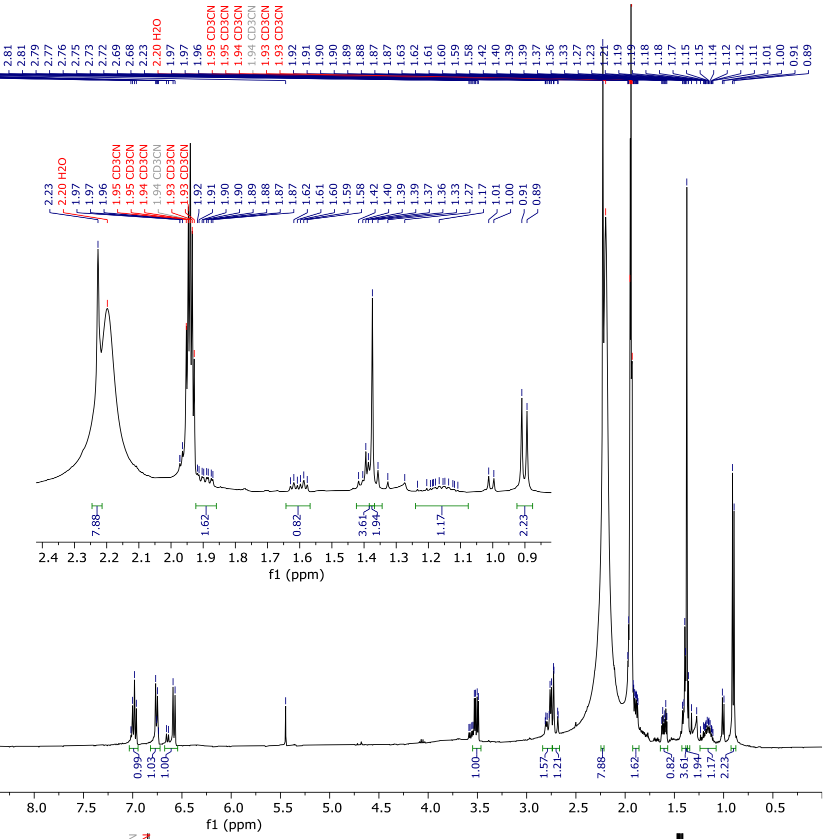

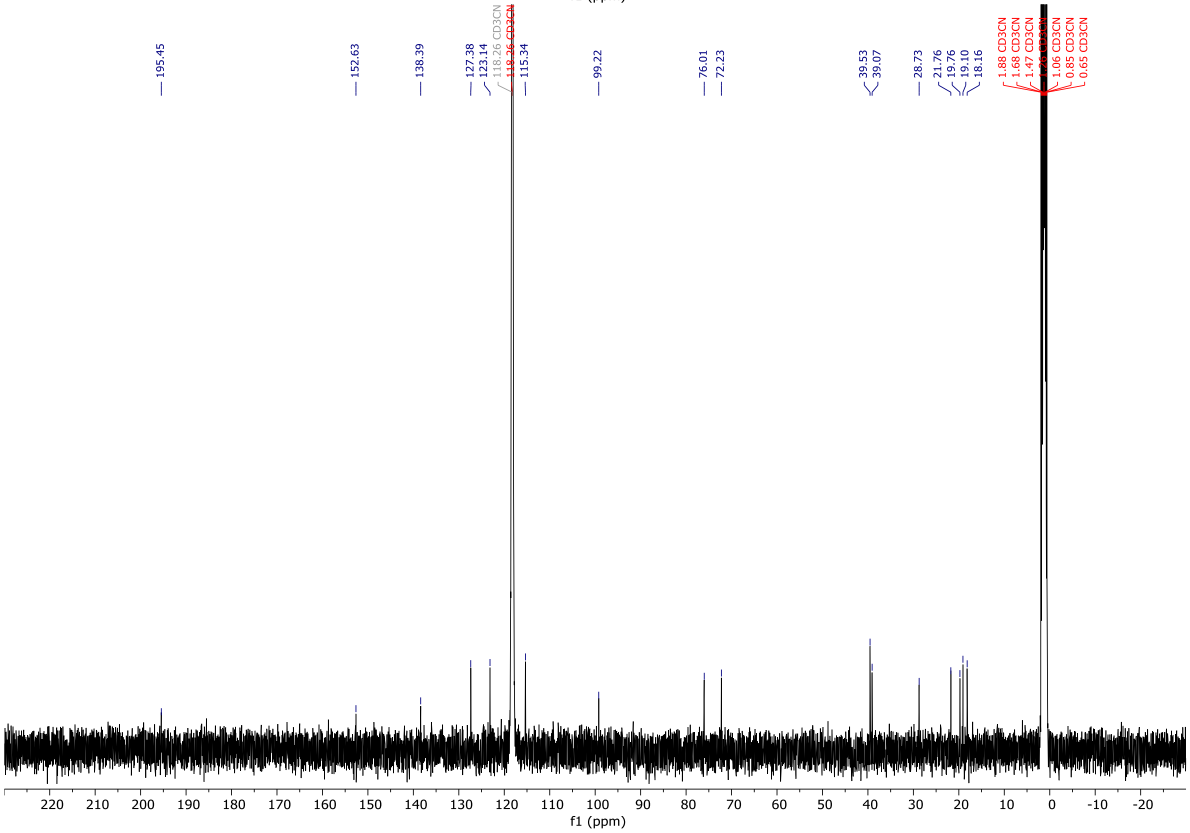

5.2.16 ¹H NMR (500 MHz, CDCl₃) and ¹³C NMR (101 MHz, CDCl₃) of compound 39

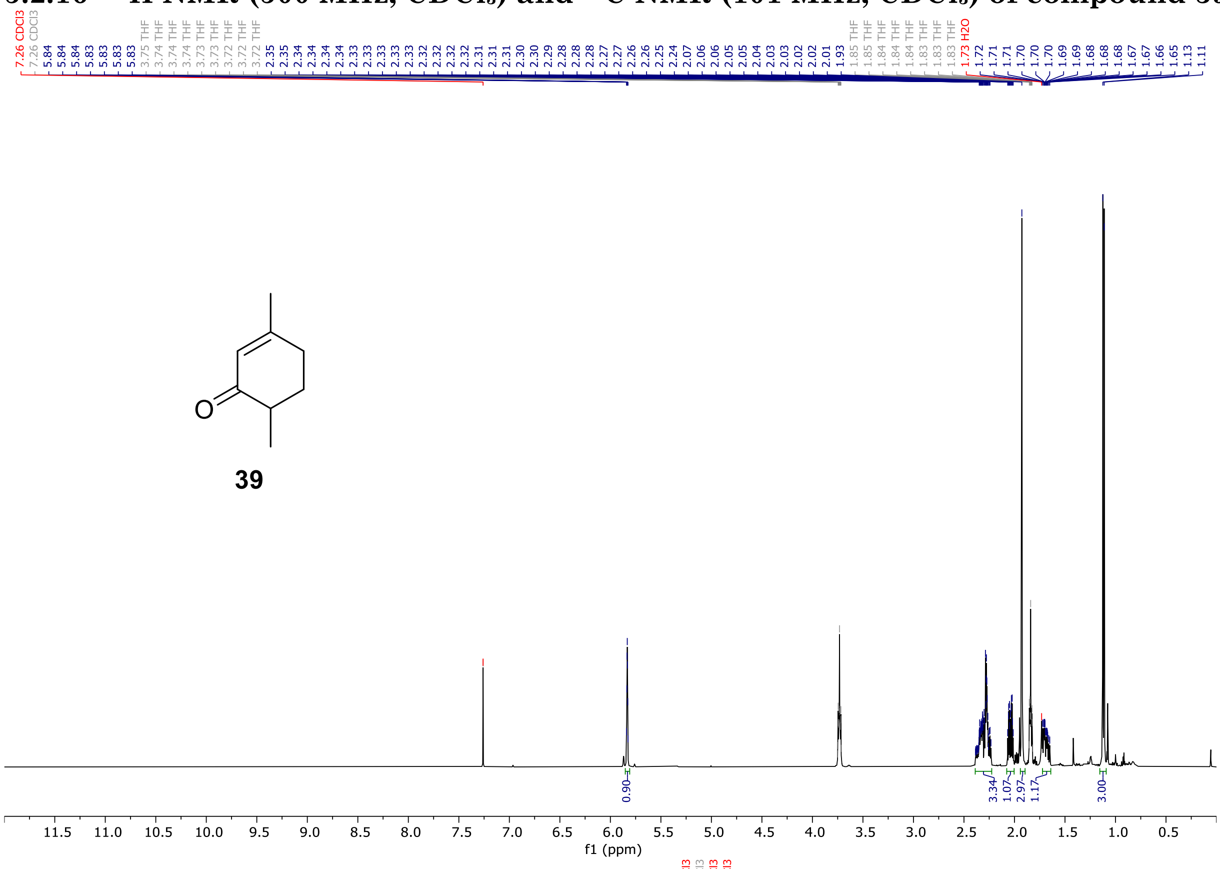

39

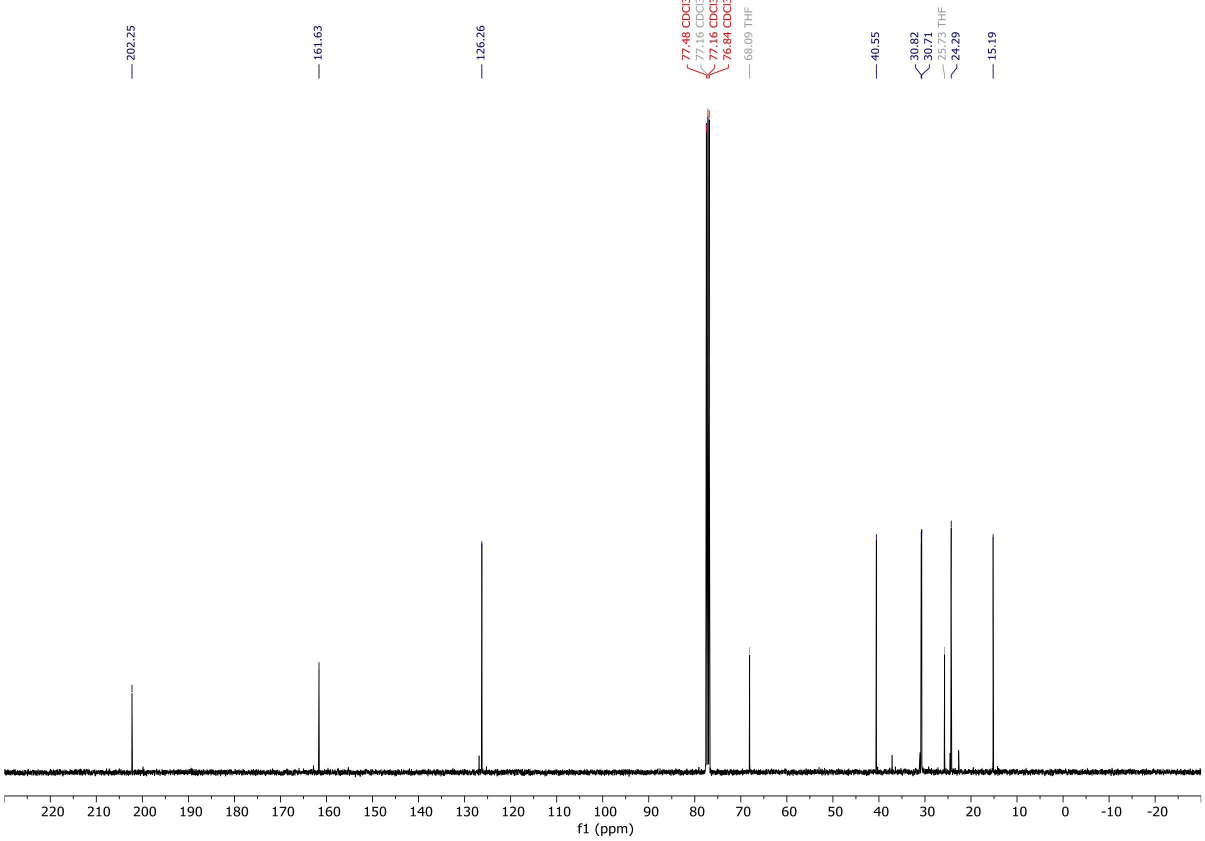

5.2.17 ^{1}H NMR (400 MHz, $CDCl_3$) and ^{13}C NMR (101 MHz, $CDCl_3$) of compound 42

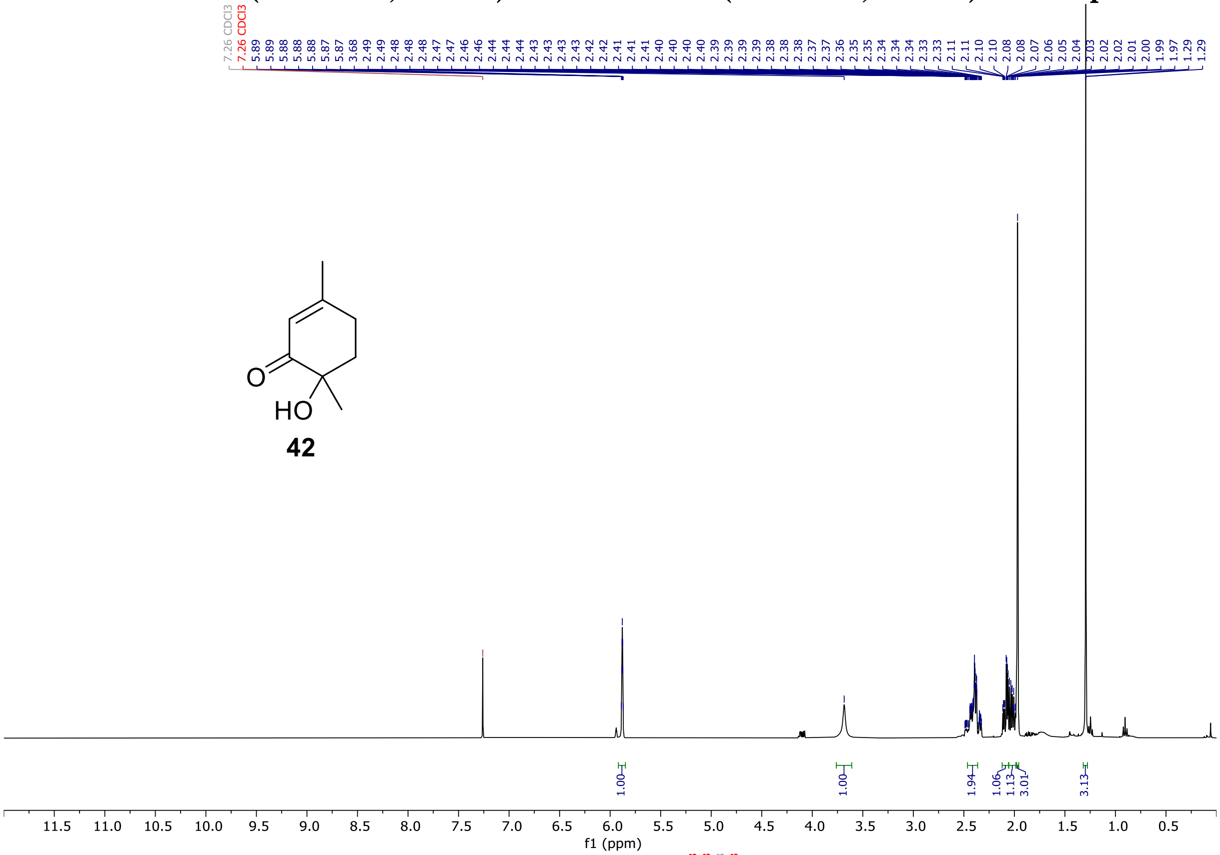

42

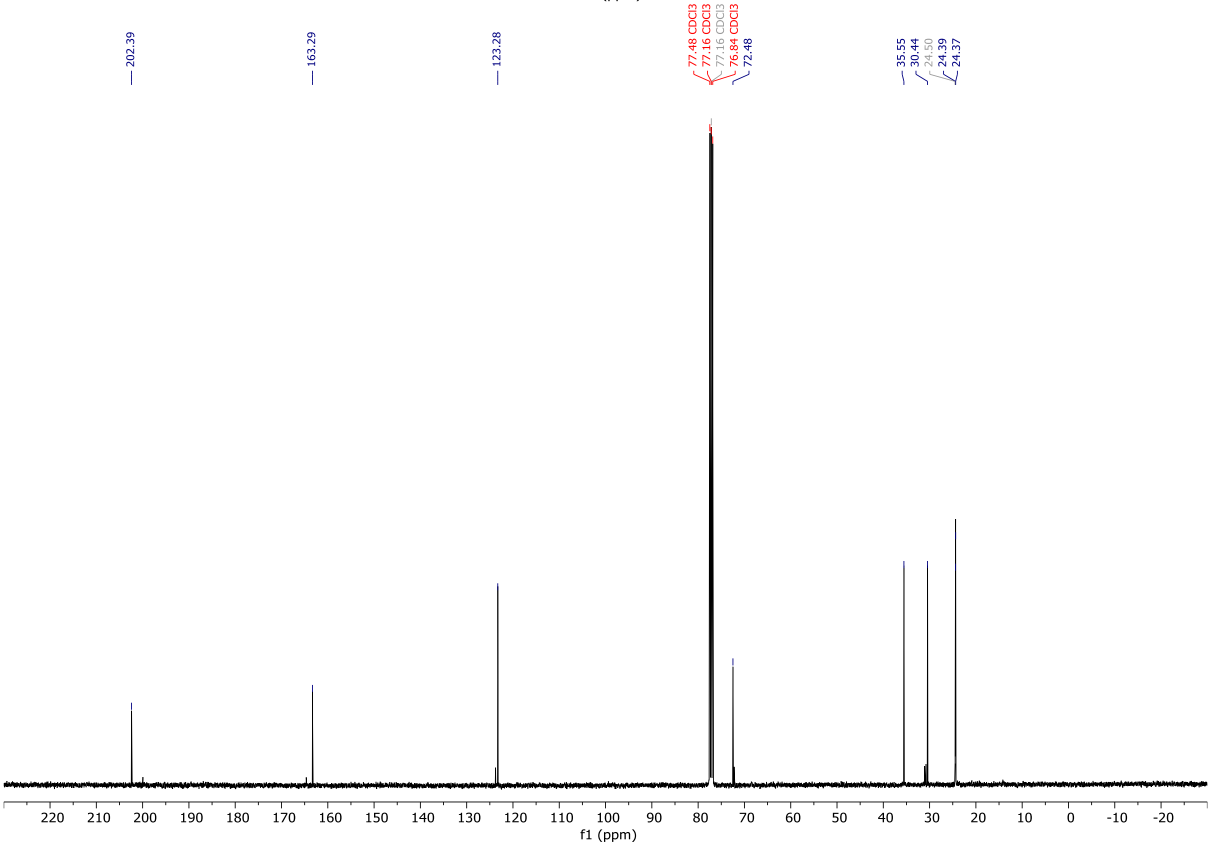

5.2.18 ^{1}H NMR (400 MHz, $CDCl_3$) and ^{13}C NMR (101 MHz, $CDCl_3$) of compound 43

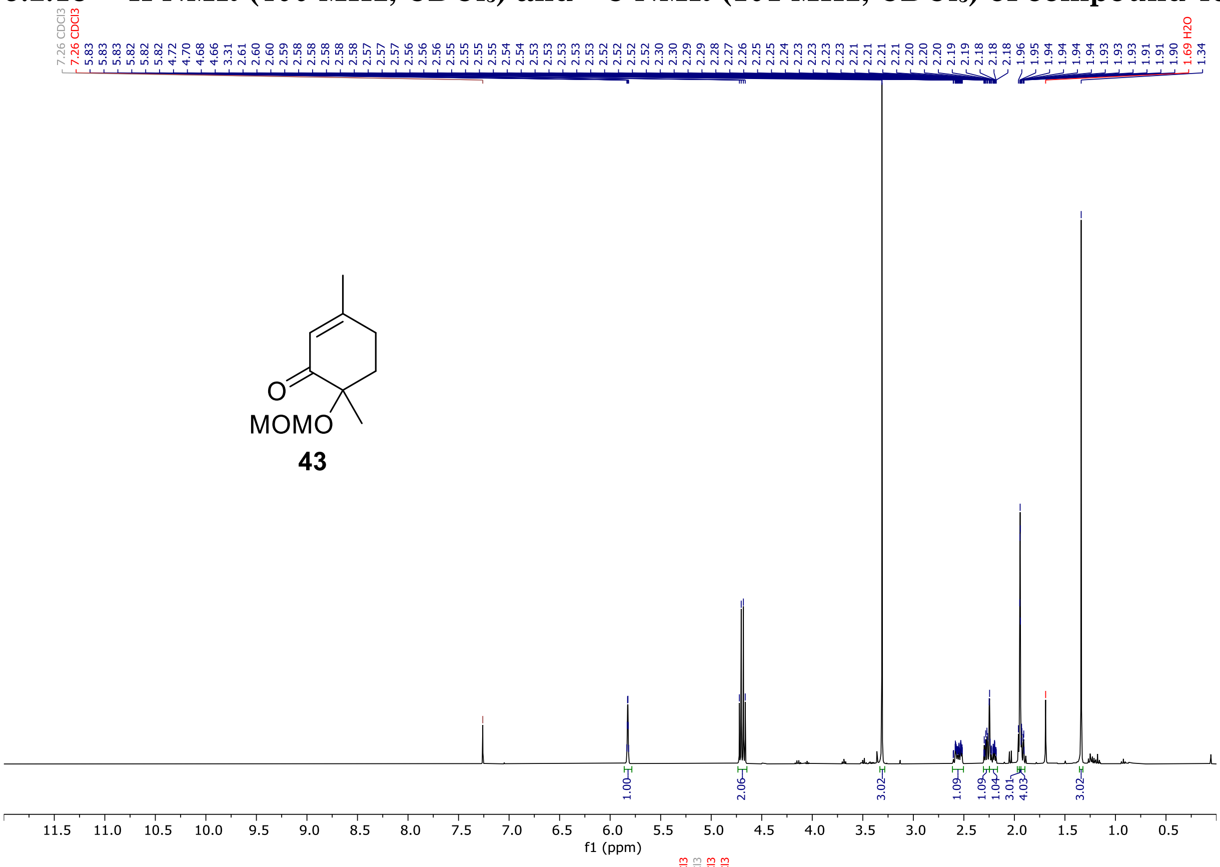

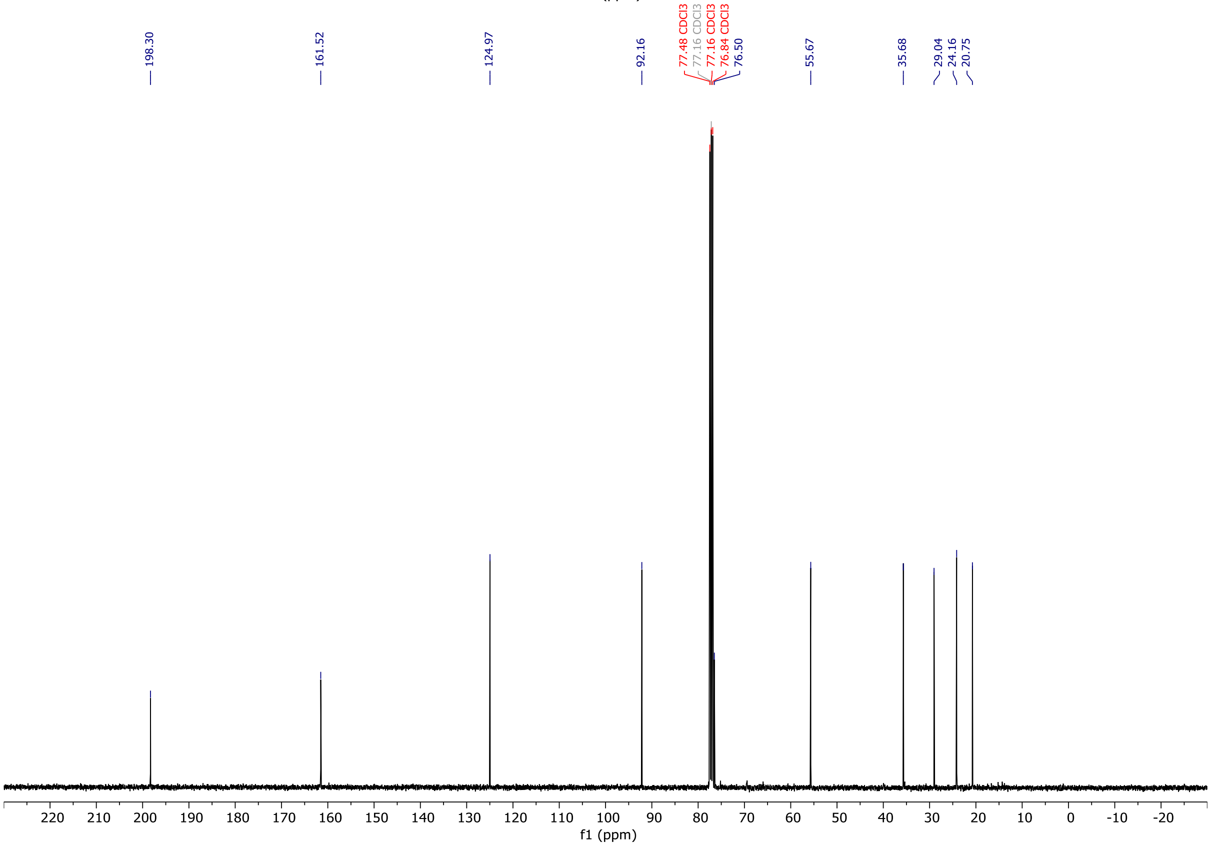

5.2.19 ^{1}H NMR (400 MHz, $CDCl_3$) and ^{13}C NMR (101 MHz, $CDCl_3$) of compound 20a

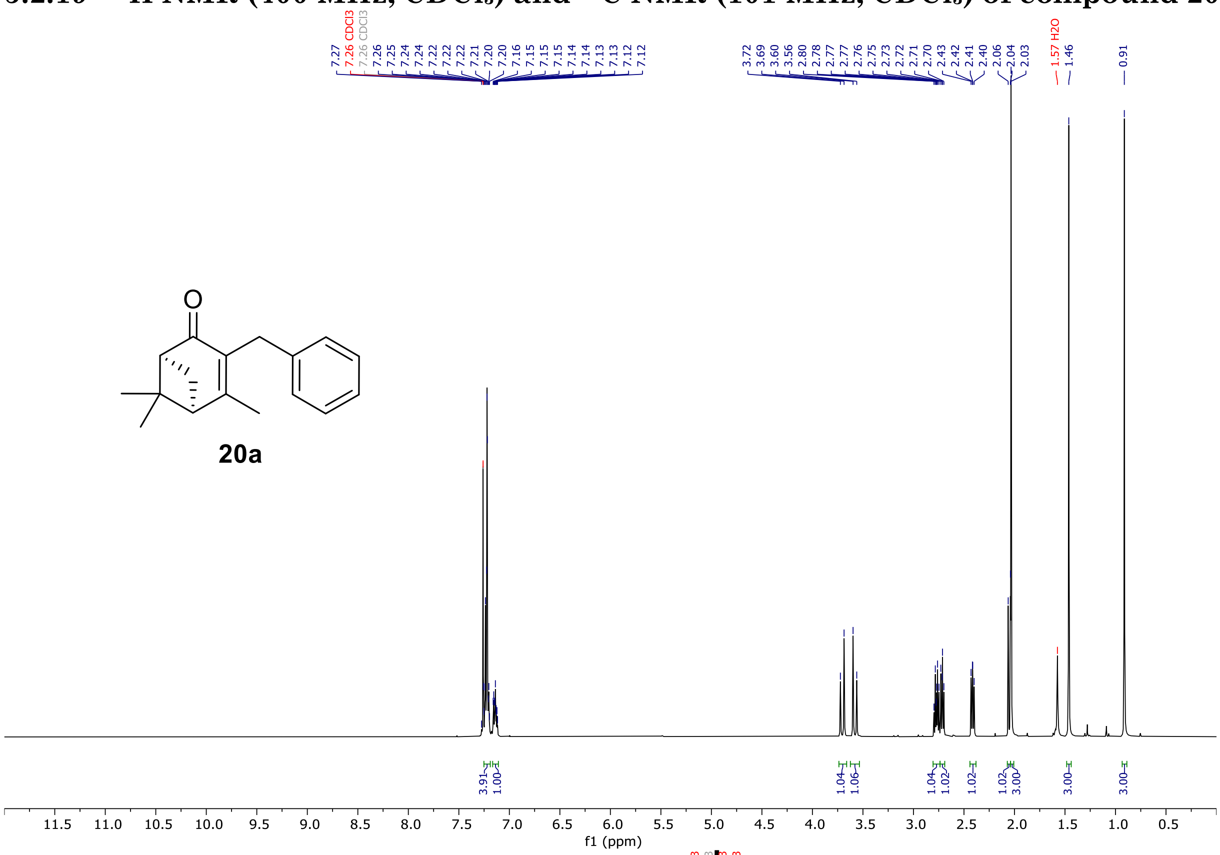

20a

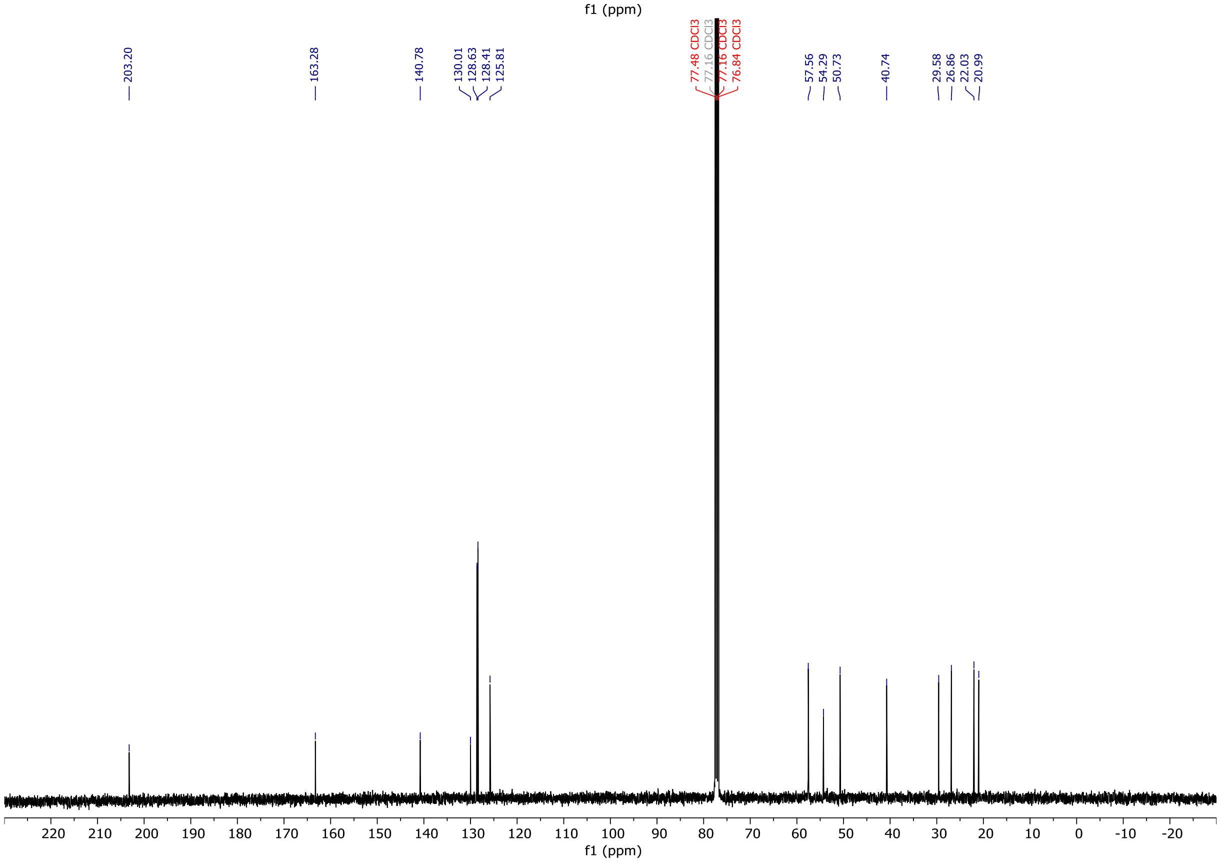

5.2.20 ^{1}H NMR (400 MHz, $CDCl_3$) and ^{13}C NMR (101 MHz, $CDCl_3$) of compound 20b

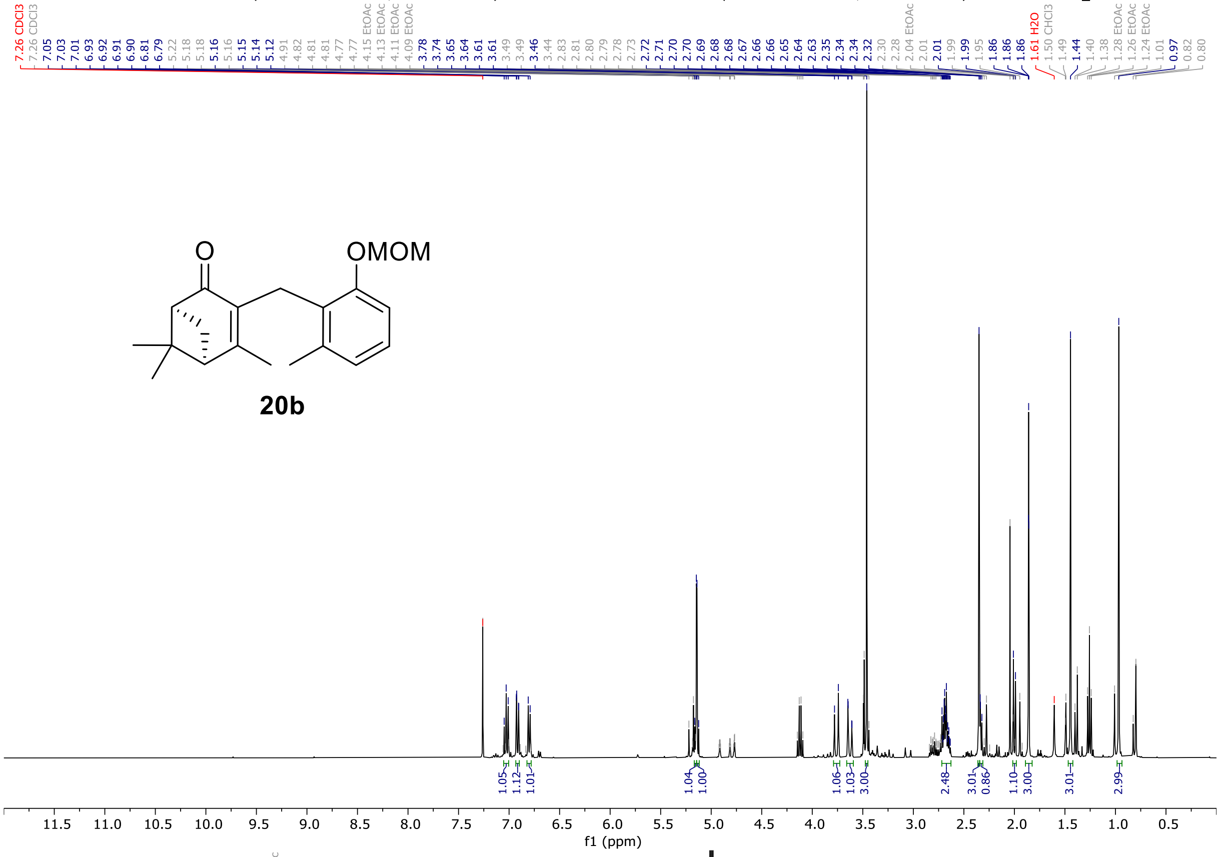

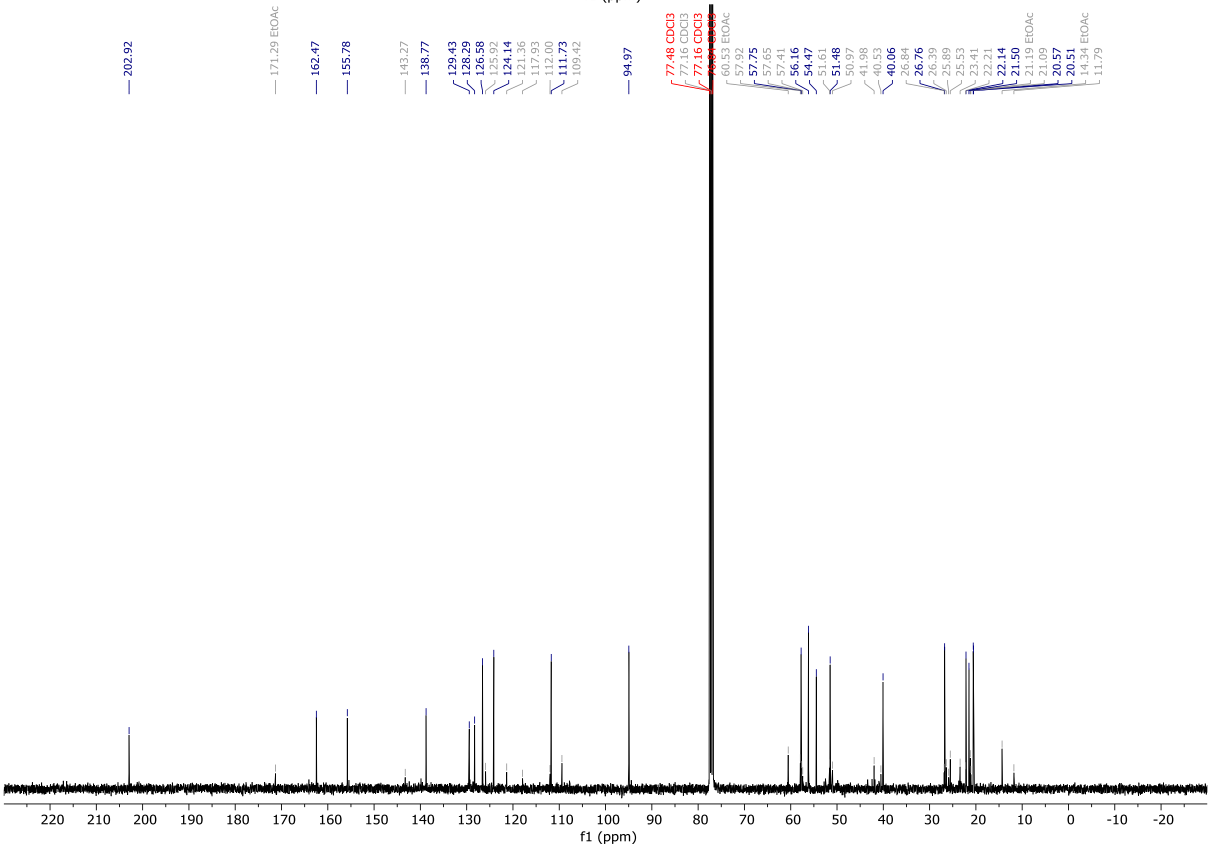

5.2.21 ^{1}H NMR (500 MHz, $CDCl_3$) and ^{13}C NMR (126 MHz, $CDCl_3$) of compound 44

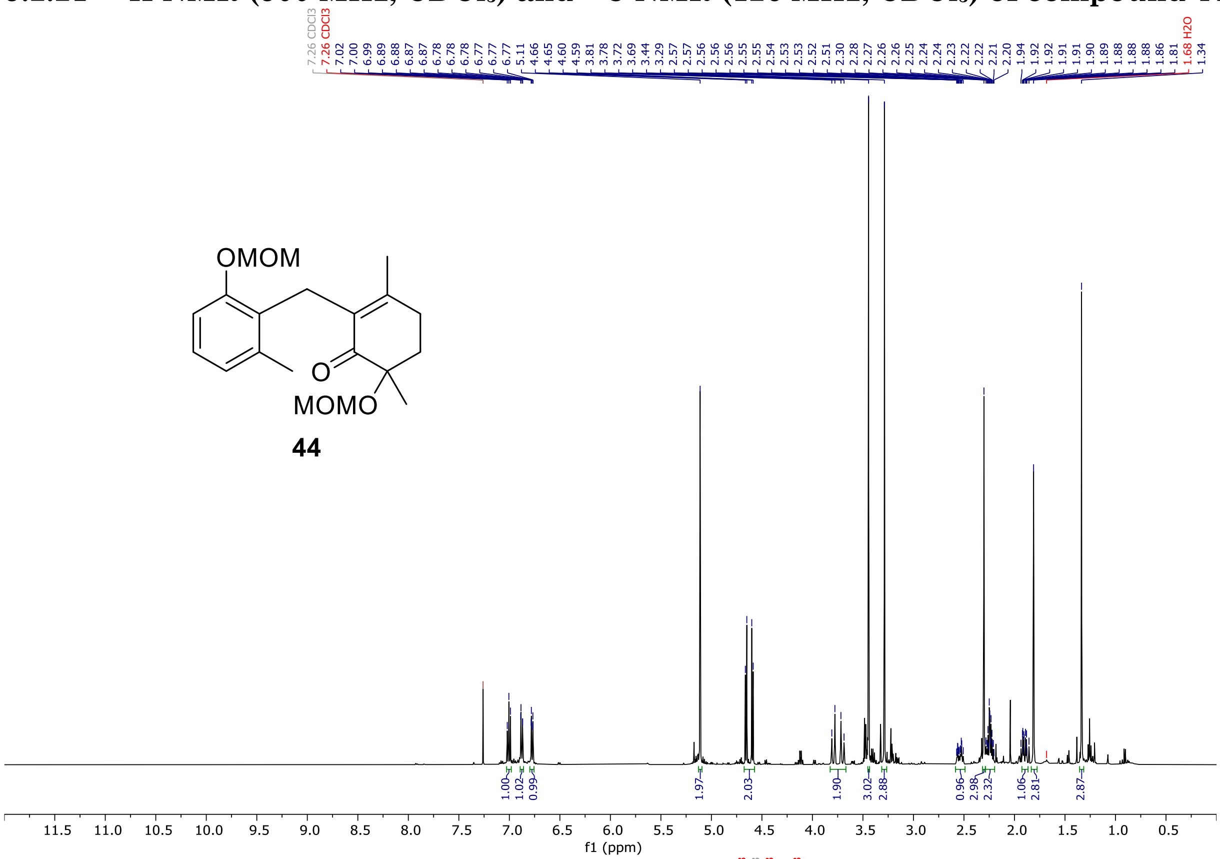

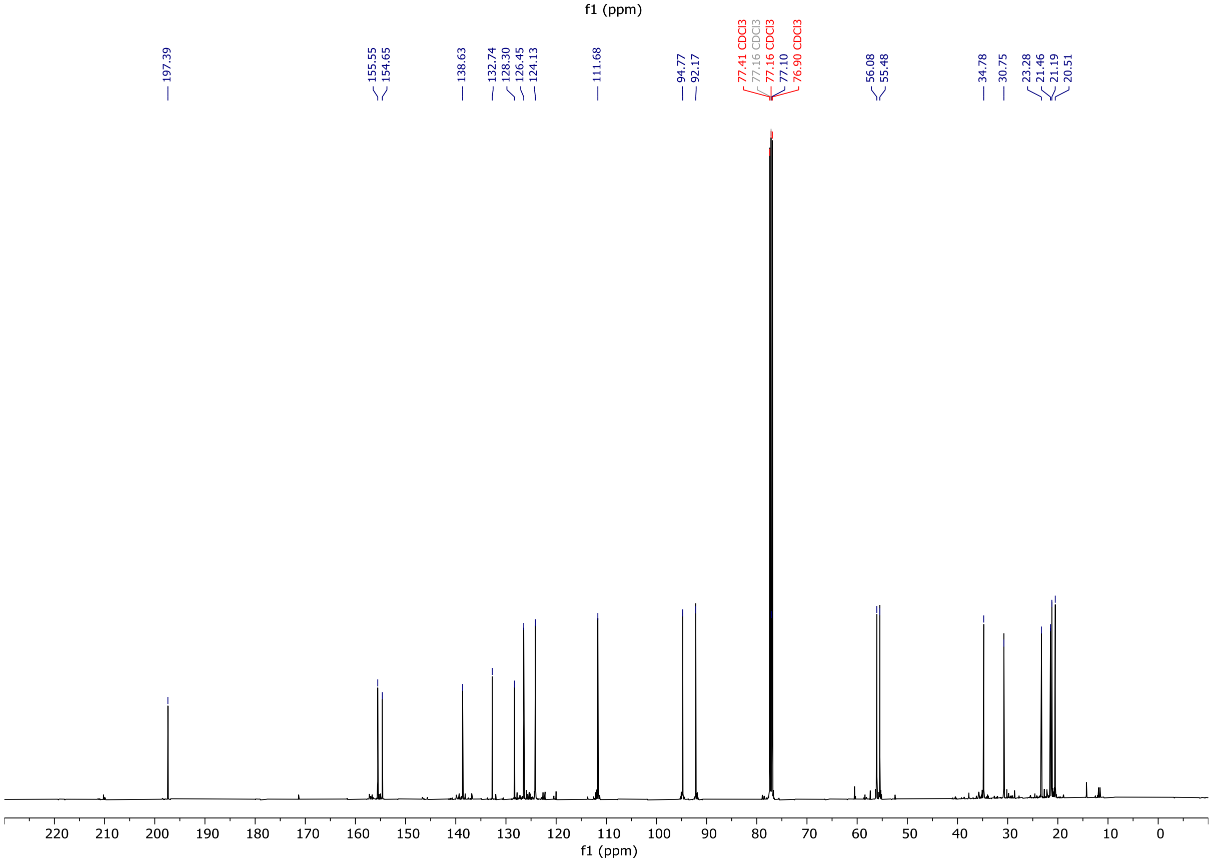

5.2.22 ^{1}H NMR (400 MHz, $CDCl_3$) and ^{13}C NMR (101 MHz, $CDCl_3$) of *rac*-11-deoxy-MikA (46)

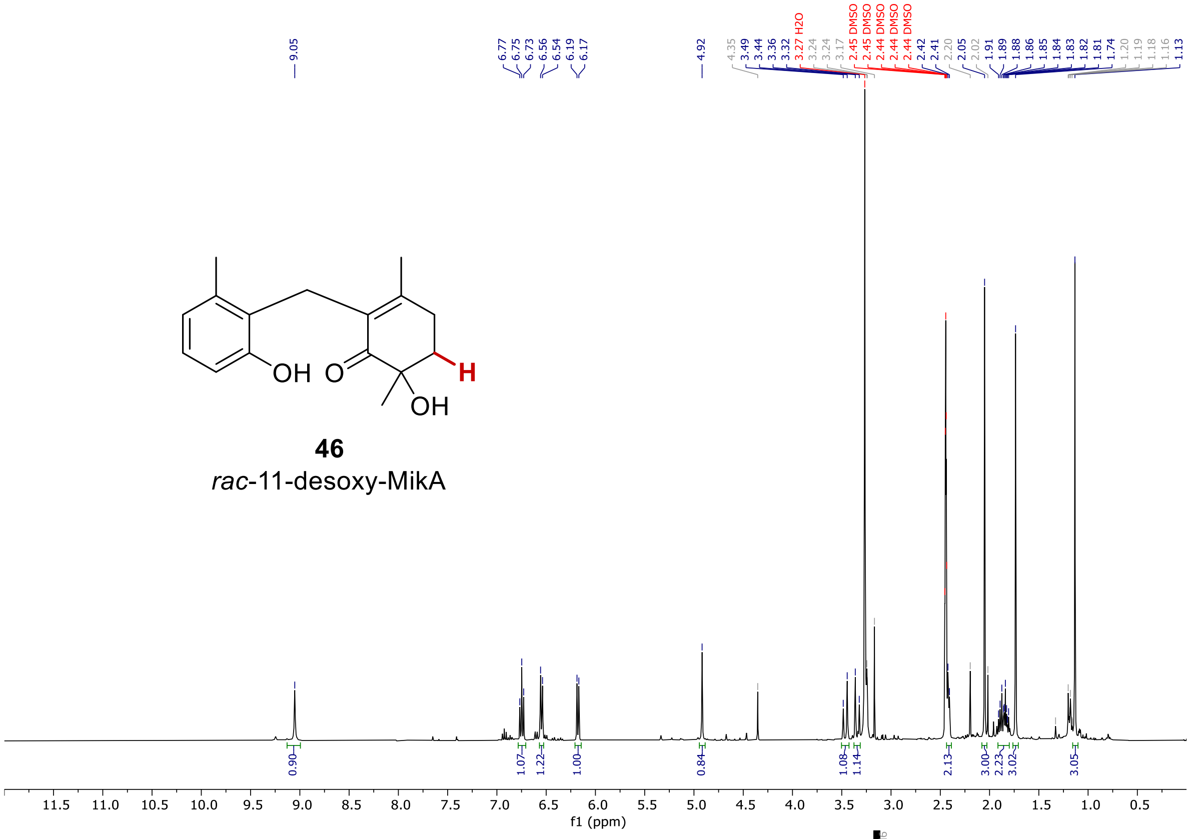

46
rac-11-desoxy-MikA

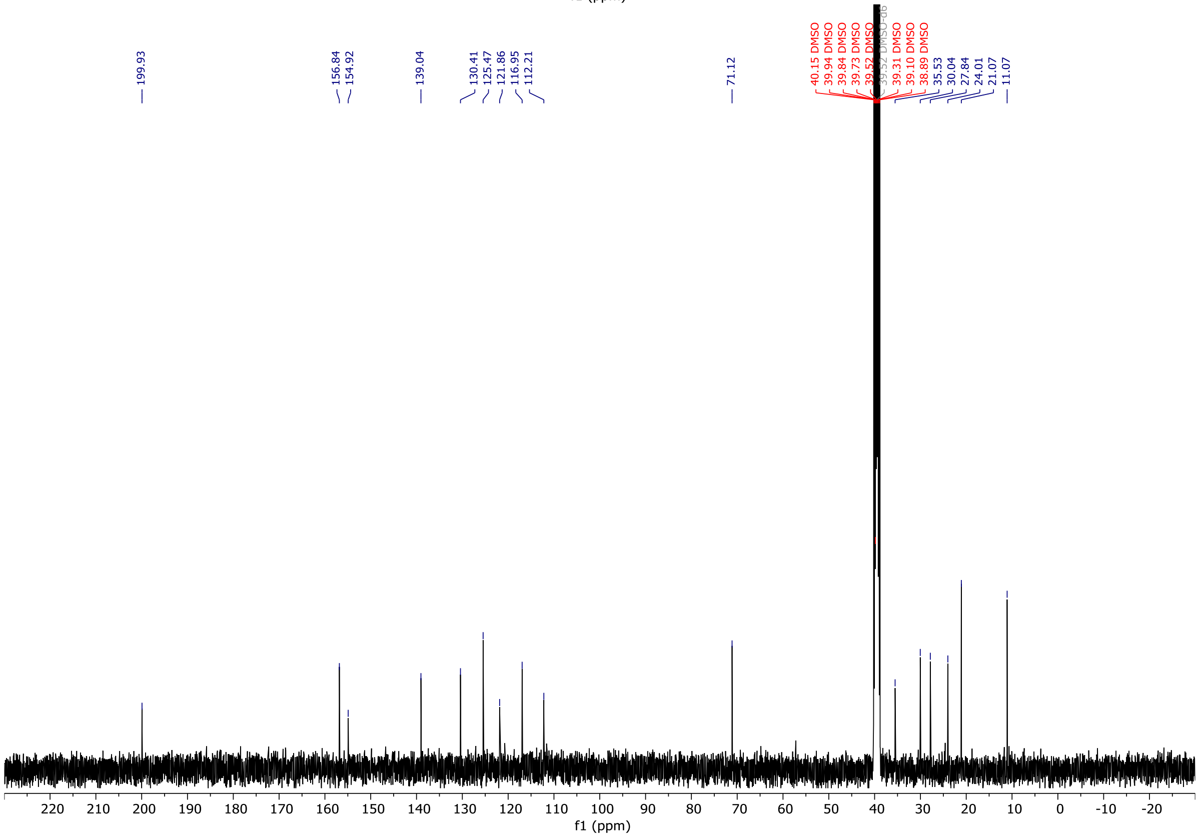

5.2.23 ^{1}H NMR (400 MHz, $CDCl_3$) and ^{13}C NMR (101 MHz, $CDCl_3$) of compound 24a

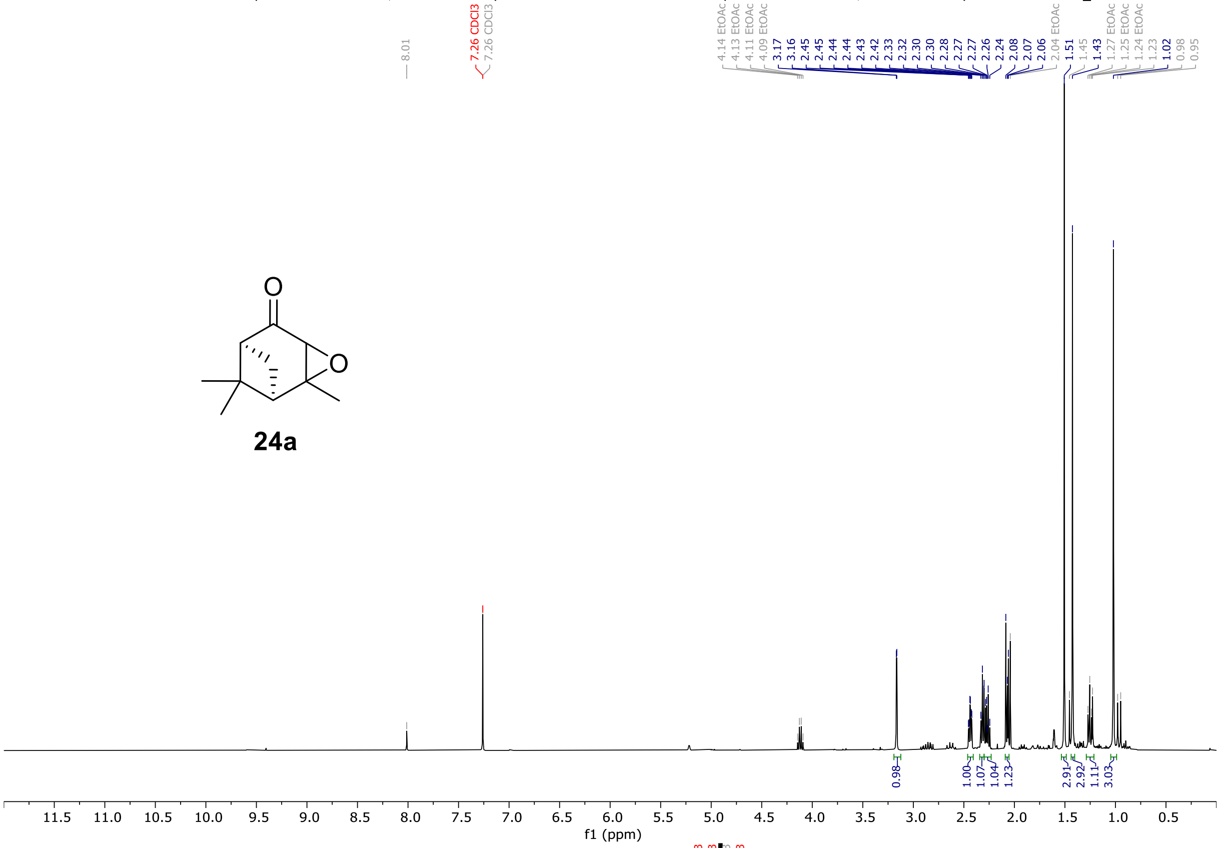

24a

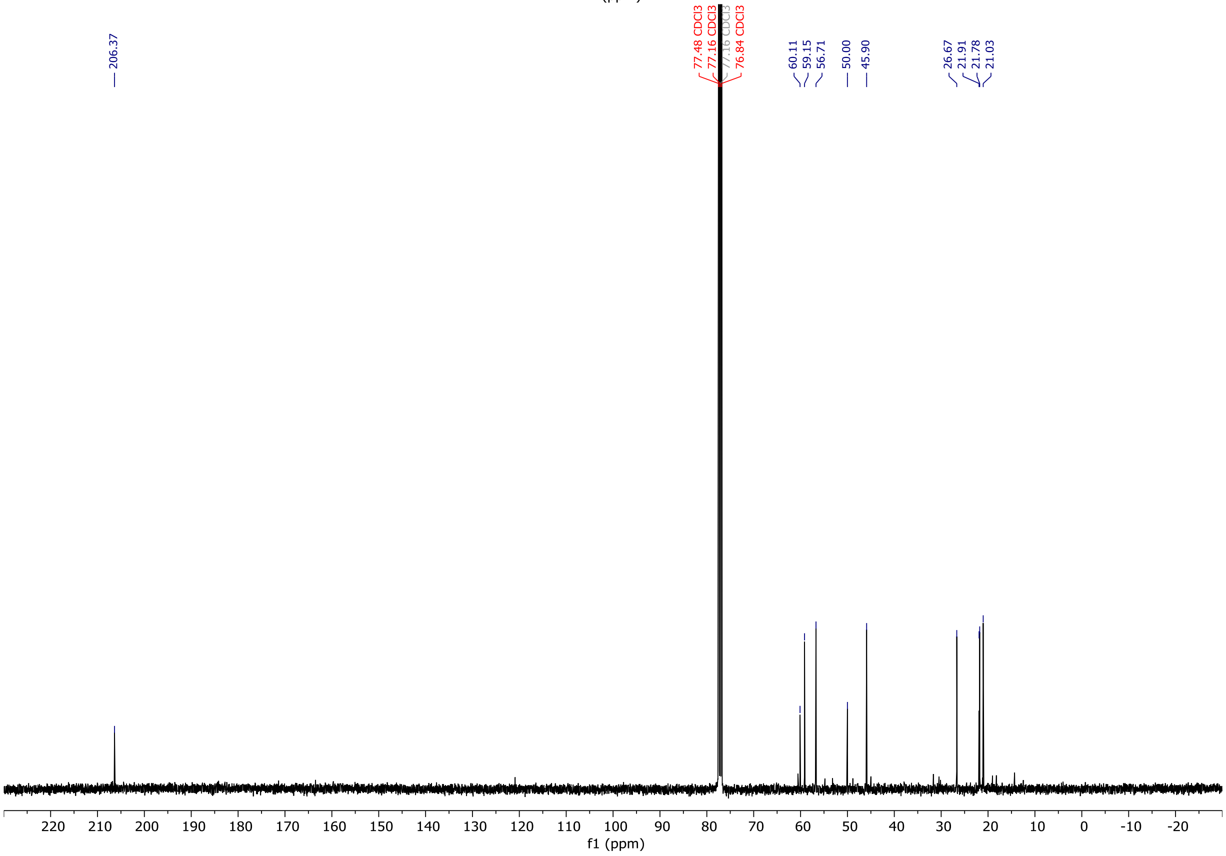

5.2.24 ^{1}H NMR (400 MHz, $CDCl_3$) and ^{13}C NMR (101 MHz, $CDCl_3$) of compound 24b

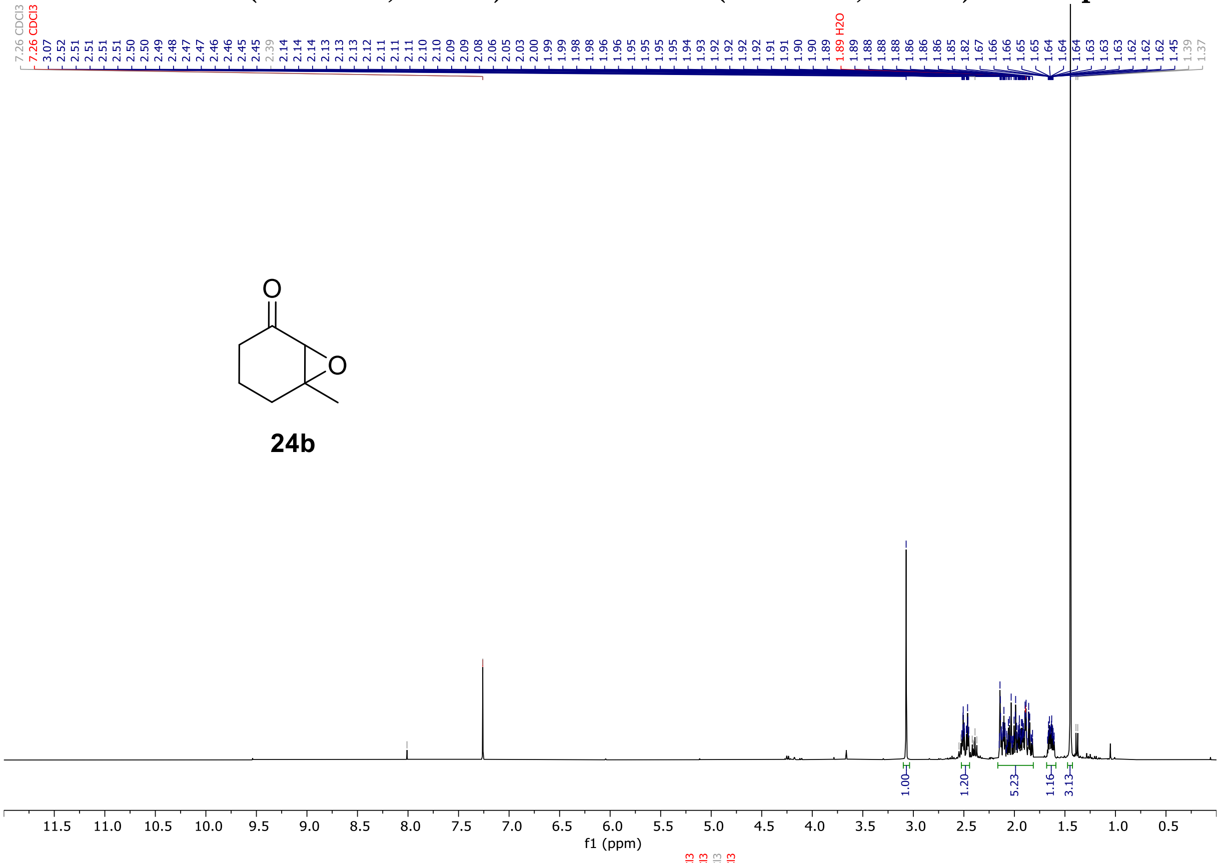

24b

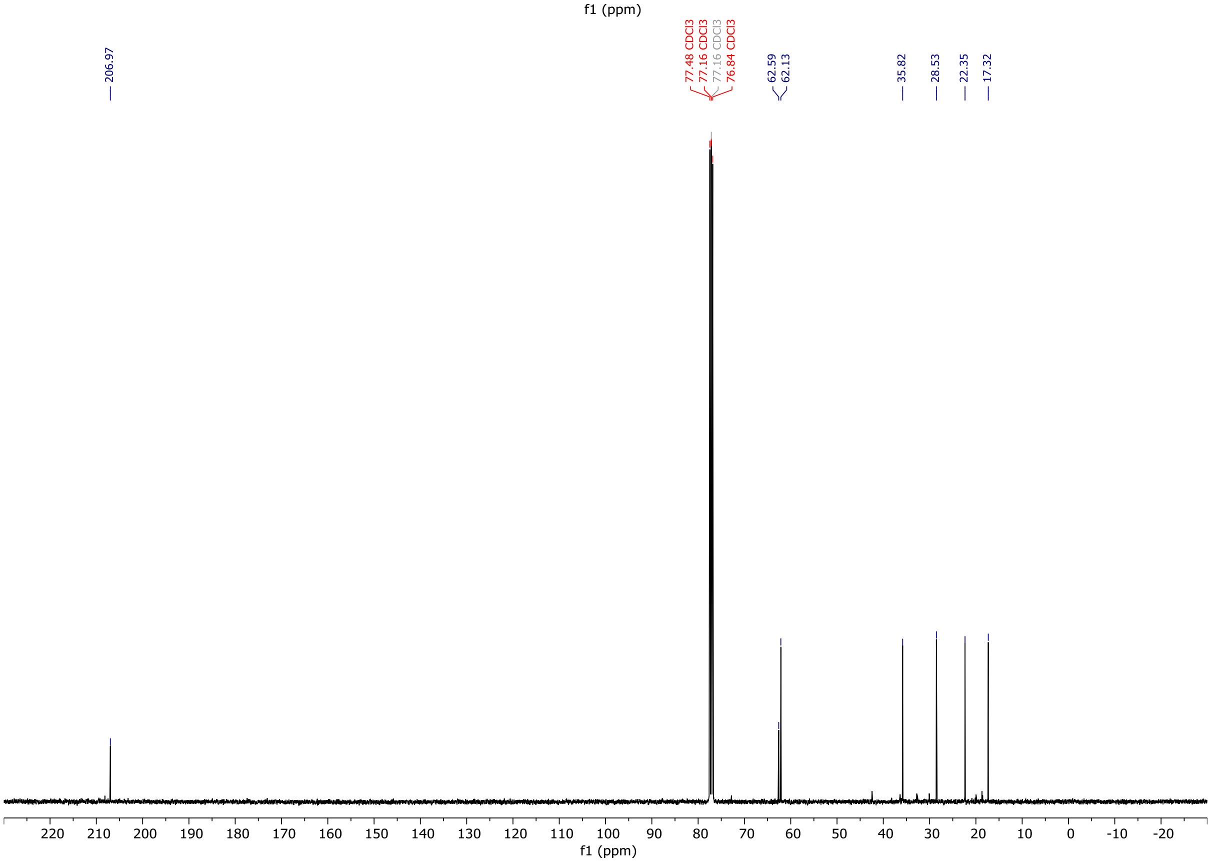

5.2.25 ^{1}H NMR (500 MHz, $CDCl_3$) of compound 26

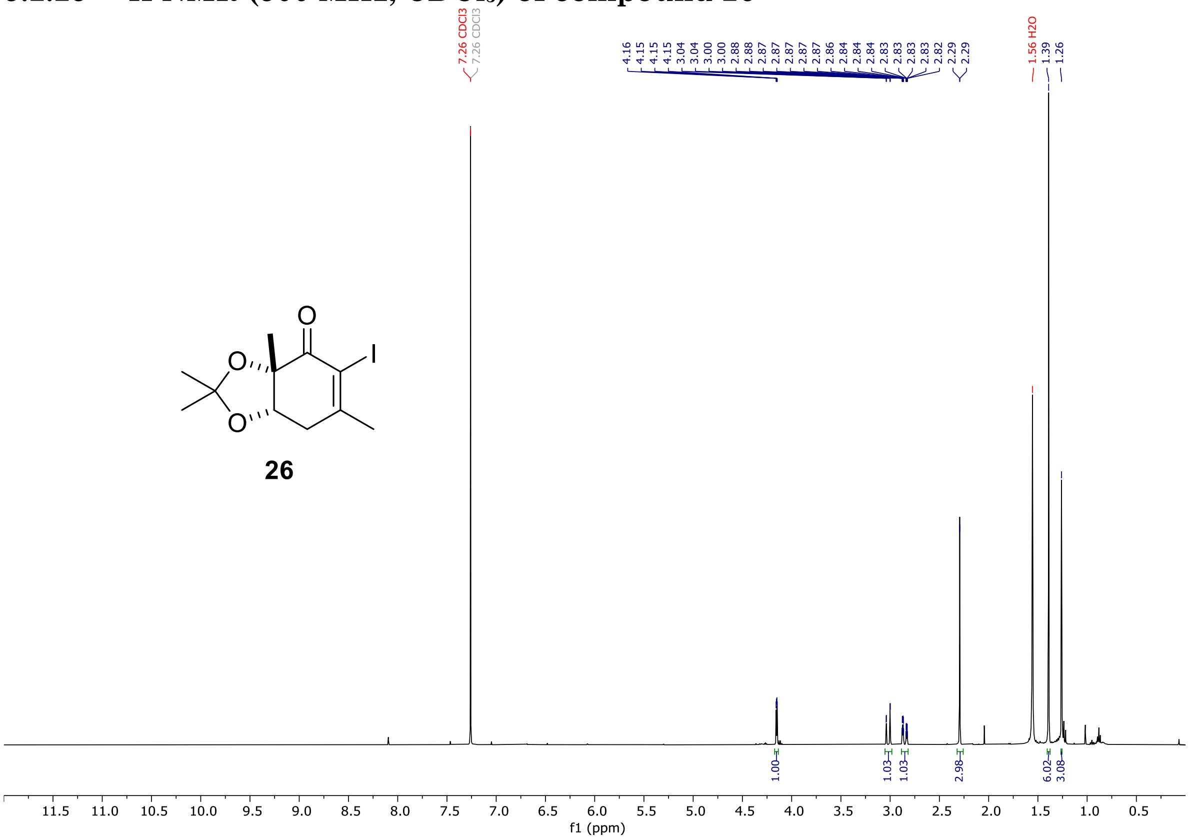

5.2.26 ^{1}H NMR (500 MHz, $CDCl_3$) of compound 32

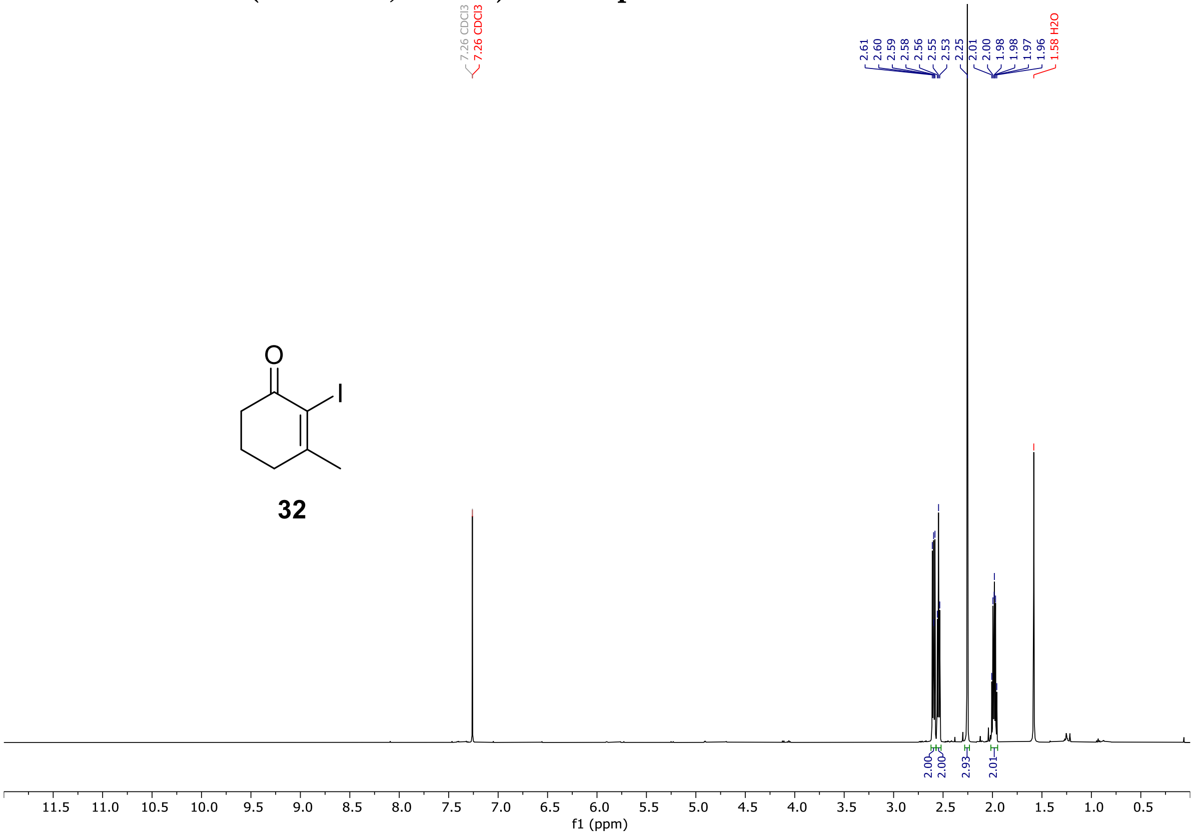

5.2.27 ^{1}H NMR (500 MHz, $CDCl_3$) and ^{13}C NMR (101 MHz, $CDCl_3$) of 2,3-dimethylphenyl acetate

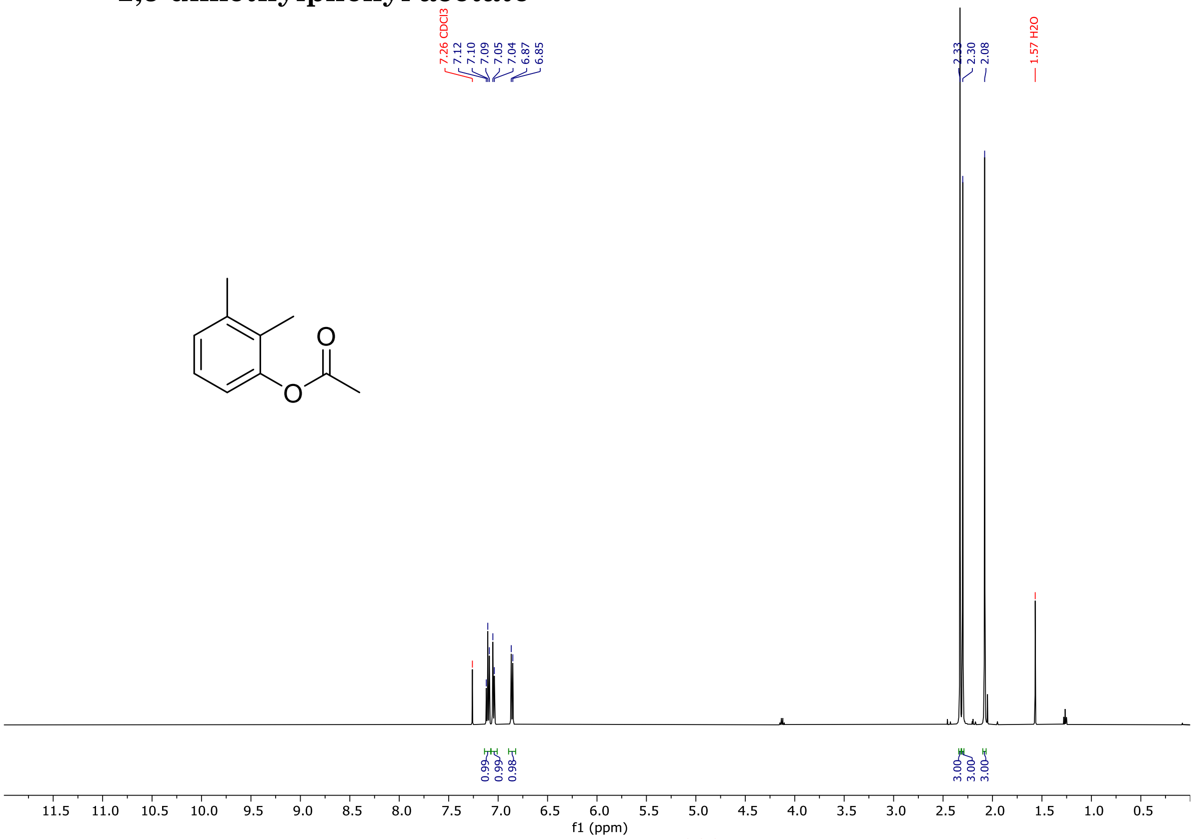

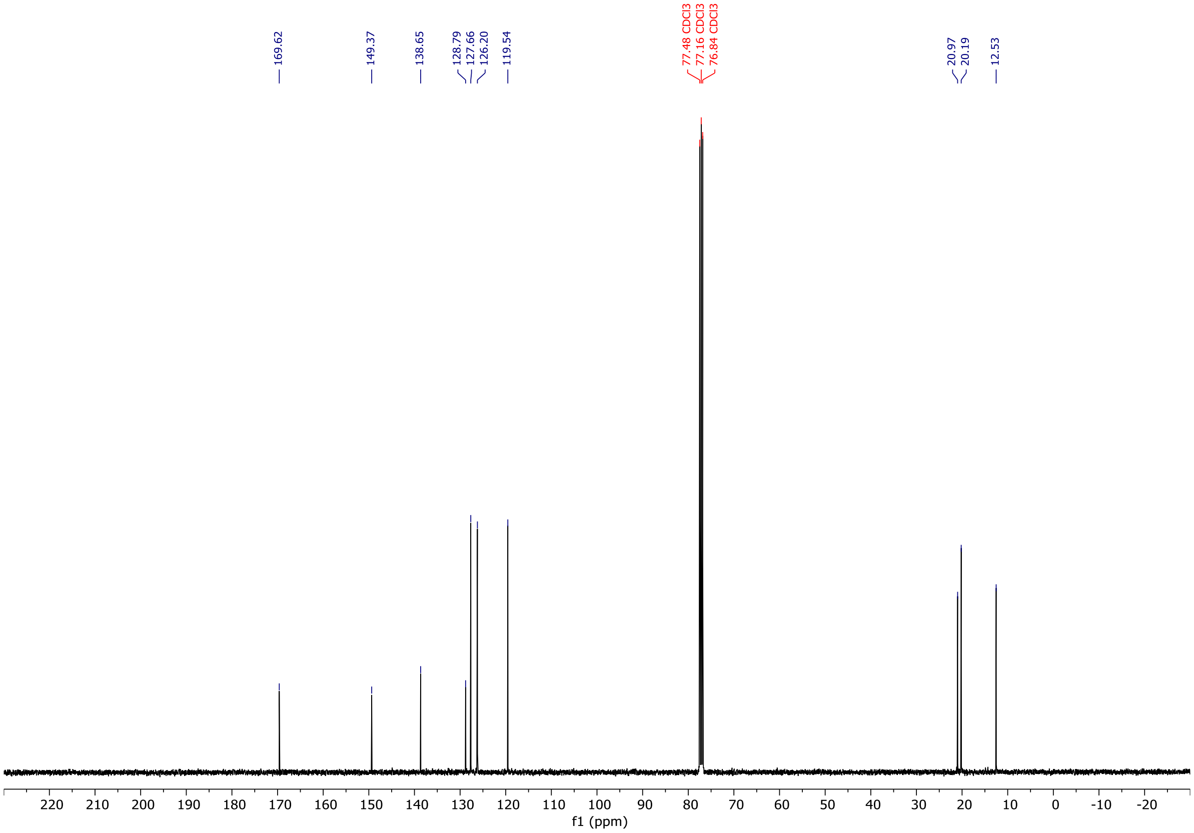

5.2.28 ^{1}H NMR (400 MHz, $CDCl_3$) and ^{13}C NMR (101 MHz, $CDCl_3$) of compound 29b

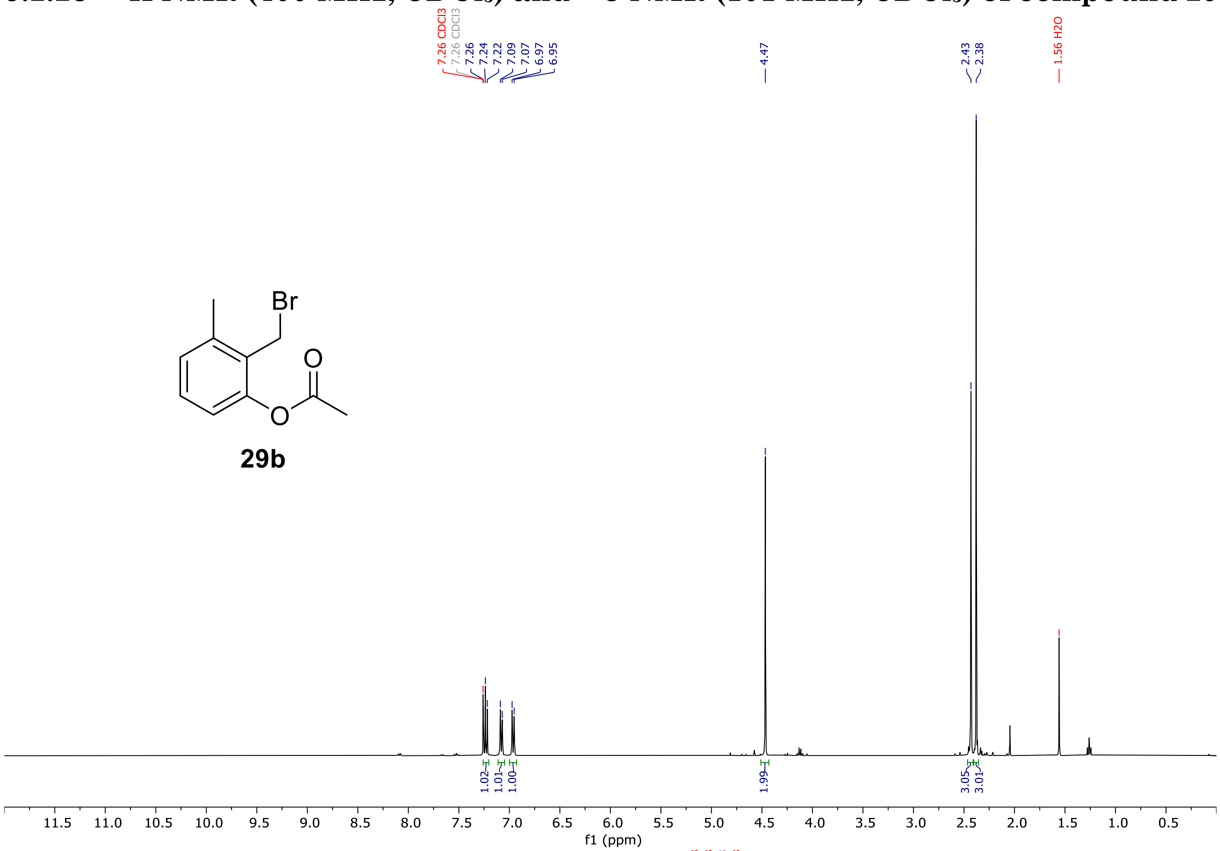

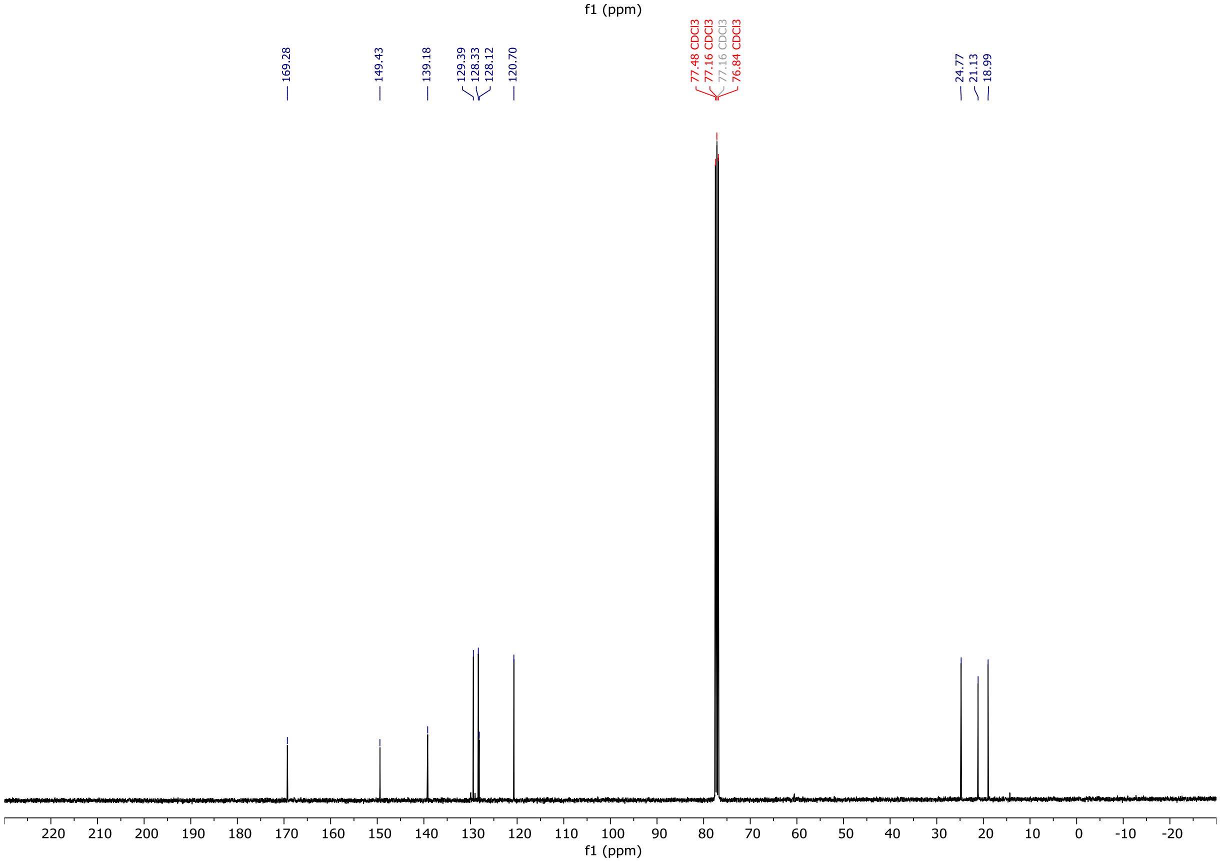

5.2.29 ^{1}H NMR (400 MHz, $CDCl_3$) of compound 31a

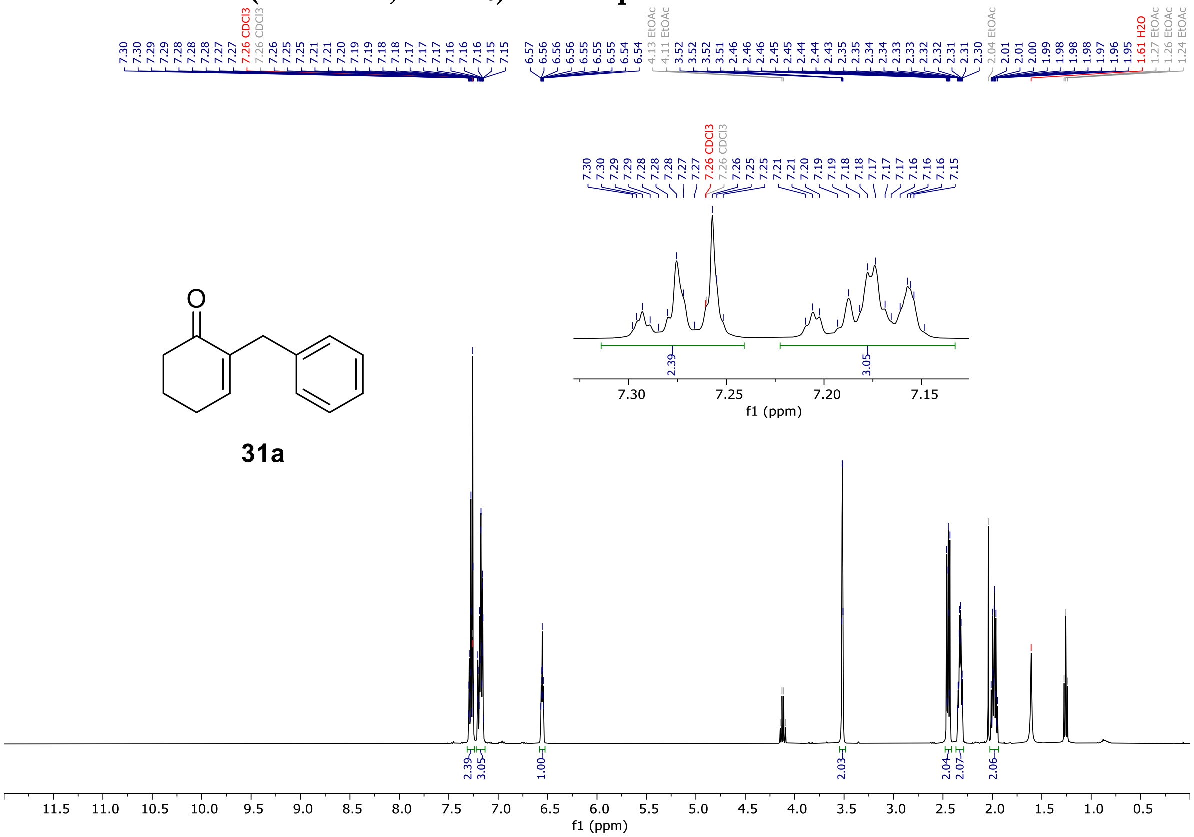

5.2.30 ^{1}H NMR (500 MHz, $CDCl_3$) of compound 33

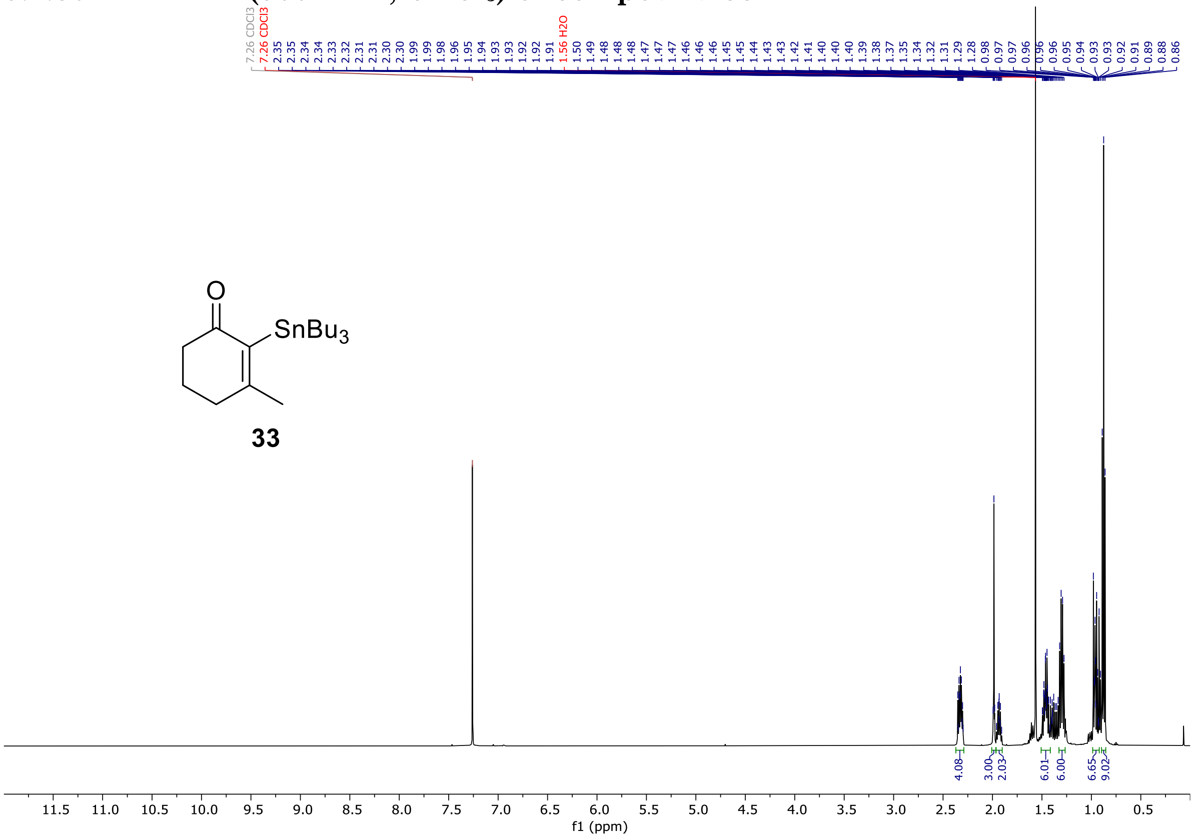

5.2.31 ^1H NMR (400 MHz, $CDCl_3$) of compound 34a

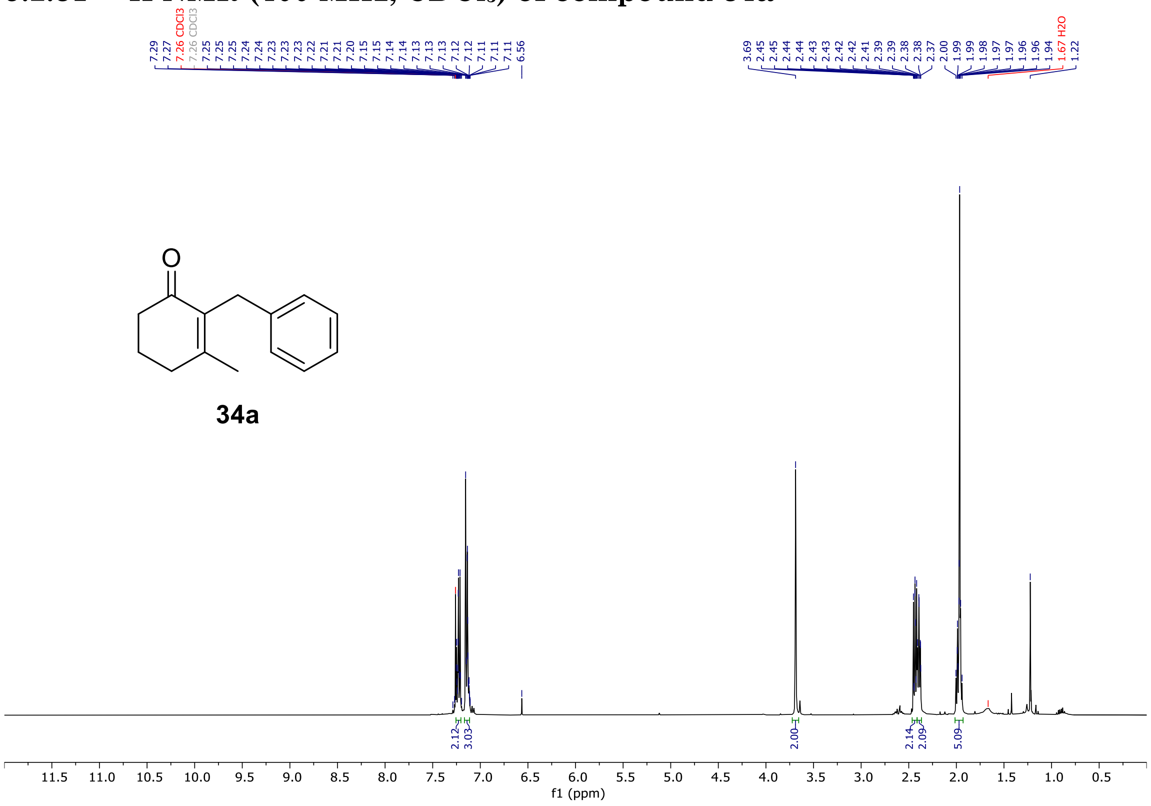

Supplement: Supplementary file 1 [file np5c01581_si_001.pdf]
